# Clinical Pharmacists, Medications, and Contingency Management for Targeting Smoking in HIV Clinics: A Randomized Clinical Trial

**DOI:** 10.1001/jamanetworkopen.2025.60593

**Published:** 2026-02-27

**Authors:** E. Jennifer Edelman, Yanhong Deng, James Dziura, Inbal Nahum-Shani, June-Marie Weiss, Lydia Aoun-Barakat, Krysten W. Bold, Dini Harsono, Colleen Mistler, Erika Payne, Sherry Aiudi, Keith M. Sigel, Jessica E. Yager, David M. Ledgerwood, Steven L. Bernstein

**Affiliations:** 1 Program in Addiction Medicine, Yale School of Medicine, New Haven, Connecticut; 2 Department of Internal Medicine, Yale School of Medicine, New Haven, Connecticut; 3 Center for Interdisciplinary Research on AIDS, Yale School of Public Health, New Haven, Connecticut; 4 Yale Center for Analytic Sciences, Yale School of Public Health, New Haven, Connecticut; 5 Department of Emergency Medicine, Yale School of Medicine, New Haven, Connecticut; 6 Department of Psychiatry, Medical School and Data-science for Dynamic Decision-making Center (d3c), Institute For Social Research, University of Michigan, Ann Arbor; 7 Department of Psychiatry, Yale School of Medicine, New Haven, Connecticut; 8 Yale New Haven Hospital, New Haven, Connecticut; 9 Department of Medicine, Icahn School of Medicine at Mount Sinai, New York, New York; 10 Department of Medicine, State University of New York Downstate Health Sciences University, Brooklyn; 11 Department of Psychiatry and Behavioral Neurosciences, Wayne State University School of Medicine, Detroit, Michigan; 12 Department of Medicine, Geisel School of Medicine at Dartmouth, Lebanon, New Hampshire

## Abstract

**Importance:**

There is a lack of robust strategies to reduce cigarette smoking among people with HIV.

**Objective:**

To identify optimal adaptive treatment strategies involving clinical pharmacist–delivered medications for tobacco use disorder and contingency management (CM) for smoking reduction among people with HIV.

**Design, Setting, and Participants:**

From July 27, 2020, through March 28, 2024, using a sequential multiple-assignment randomized clinical trial, people with HIV who smoked cigarettes were recruited and randomized 1:1 to nicotine replacement therapy (NRT) with or without CM (stage 1). After 12 weeks of treatment (stage 2), individuals with confirmed abstinence continued stage 1 treatment; individuals without confirmed abstinence were rerandomized to switch to oral medications for tobacco use disorder or to intensified CM. Interventions were delivered by clinical pharmacists in HIV clinics across 24 weeks.

**Interventions:**

Stage 1 included NRT with or without CM (rewards for confirmed abstinence). Stage 2 included either a switch to varenicline or bupropion or intensification to more rewards for abstinence. Each stage involved 5 clinical pharmacist visits.

**Main Outcomes and Measures:**

The primary outcome was cigarettes per day (CPD), and the secondary outcome was 7-day confirmed abstinence at 12 and 24 weeks with imputation. Analyses were conducted using intention-to-treat principles.

**Results:**

In total, 323 participants (181 [56.0%] male at birth; 317 [72.5%] Black or African American; mean [SD] age, 55.1 [10.7] years) smoked a mean (SD) of 12.8 (7.2) CPD at baseline. At 12 weeks, participants in the NRT plus CM (least-squares mean [LSM], 4.9 [97.5% CI, 3.5-6.2] CPD) and NRT (LSM, 5.2 [97.5% CI, 3.9-6.5] CPD) groups smoked similar numbers of CPD (adjusted LSM difference, −0.3 [97.5% CI, −1.9 to 1.3]; *P* = .66). Abstinence was greater at 12 weeks in the NRT plus CM group (36 of 160 [22.5%]) compared with the NRT group (16 of 163 [9.8%]) (adjusted odds ratio [AOR], 2.70 [99.0% CI, 1.19-6.14]; *P* = .002). Among participants without week 12 abstinence, the effect of intensifying vs switching on week 24 CPD varied by stage 1 treatment. Intensifying was better among individuals starting with NRT alone (adjusted LSM difference, −3.8 [97.5% CI, −6.0 to −1.5] CPD; *P* < .001) but not among individuals initially receiving NRT plus CM (adjusted LSM difference, 0.3 [97.5% CI, −2.1 to 2.6] CPD; *P* = .80). Abstinence at 24 weeks was similar among individuals in intensified vs switched groups regardless of stage 1 treatment (AOR, 1.5 [99% CI, 0.4-4.2]; *P* = .37; *P* = .85 for interaction). Overall, 24-week CPD was lowest with the adaptive treatment strategy involving NRT followed by NRT plus CM (eg, LSM, 2.6 [99% CI, 1.1-4.1] CPD vs 4.2 [99% CI, 2.6-5.9] CPD for NRT plus CM followed by NRT plus CM intensified), and abstinence was highest for the NRT plus CM followed by the intensified strategy (eg, LSM, 30.0% [99% CI, 16.2%-48.7%] vs 12.8% [99% CI, 5.2%-28.3%] for NRT followed by NRT plus CM intensified).

**Conclusions and Relevance:**

In this randomized clinical trial of people with HIV who smoked cigarettes, CM was an effective adjunct to clinical pharmacist–delivered NRT for improving tobacco-related outcomes. Optimal timing to add CM differed based on treatment goals.

**Trial Registration:**

ClinicalTrials.gov Identifier: NCT04490057

## Key Points

**Question:**

What are optimal clinical pharmacist–delivered treatment strategies for promoting cigarette smoking reduction among people with HIV?

**Findings:**

In this randomized clinical trial involving 323 participants, those receiving nicotine replacement therapy (NRT) with or without contingency management (CM) had similar reductions in cigarettes per day (CPD) at 12 weeks. Among participants who started with NRT alone and did not achieve week 12 abstinence, adding CM led to lower CPD than switching to oral medications. Participants who started with NRT and then added CM achieved lowest CPD at week 24.

**Meaning:**

Study findings, indicating that CM is an effective adjunct to clinical pharmacist–delivered NRT for improving tobacco-related outcomes, provide HIV clinics with guidance on strategies for addressing cigarette smoking reduction among people with HIV.

## Teaser

This randomized clinical trial assesses optimal adaptive treatment strategies involving clinical pharmacist–delivered medications for tobacco use disorder and contingency management for smoking reduction among people with HIV.

## Introduction

Cigarette smoking and tobacco use disorder (TUD) are prevalent and major threats to the health of people with HIV.^1^ Smoking causes more lost life-years than treated HIV, and people with HIV who smoke are less likely to quit than the general population.^2,3,4^ Smoking cessation improves quality of life and decreases morbidity and mortality.^4^ Smoking reduction may be more realistic, also improves health outcomes, and is achievable.^3,5,6^

Medications for TUD (MTUD) (ie, nicotine replacement therapy [NRT], varenicline, and bupropion) and behavioral interventions are recommended for smoking cessation.^7,8,9,10,11,12^ The optimal approach to improve smoking outcomes among people with HIV is not clear,^13^ with prior study limitations. First, only 1 incorporated contingency management ([CM], ie, tangible reinforcers to reward verifiable completion of goal attainment) with first-line MTUD despite the strong evidence of CM for abstinence^14,15^ and promising data supporting its integration in HIV clinics.^16,17,18^ Second, clinical pharmacists are increasingly involved in treatment of people with HIV,^19^ routinely prescribe MTUD,^20,21,22,23^ and provide a solution to HIV clinician–reported barriers to addressing TUD.^24,25,26^ Attention to the potential of clinical pharmacists to prescribe MTUD in HIV clinics has been limited.^27,28,29,30,31^ Additionally, to our knowledge, no studies have evaluated an adaptive treatment strategy (ATS)^32^ including first-line MTUD with CM to reduce smoking among people with HIV, even though treatment is not uniformly effective.^33^

We conducted a sequential multiple-assignment randomized trial (SMART),^34,35^ A SMART Approach to Treating Tobacco Use Disorder in Persons With HIV (SMARTTT), to (1) compare the efficacy of clinical pharmacist–delivered NRT plus CM to NRT alone on smoking reduction and abstinence at week 12; (2) compare the impact of switching to oral MTUD and intensifying CM on week 24 smoking behaviors among participants who did not respond to initial treatment, and (3) identify the optimal ATS to improve smoking outcomes at week 24. We hypothesized that (1) compared with NRT alone, NRT plus CM would lead to greater improvements in smoking outcomes; (2) switching to oral MTUD would lead to greater improvements in smoking outcomes compared with intensifying CM; and (3) the optimal ATS to promote greater improvements in smoking outcomes at week 24 would likely be to begin with NRT plus CM; if response, continue NRT plus CM; if nonresponse, provide oral MTUD plus CM.

## Methods

### Overview

**Figure 1.**
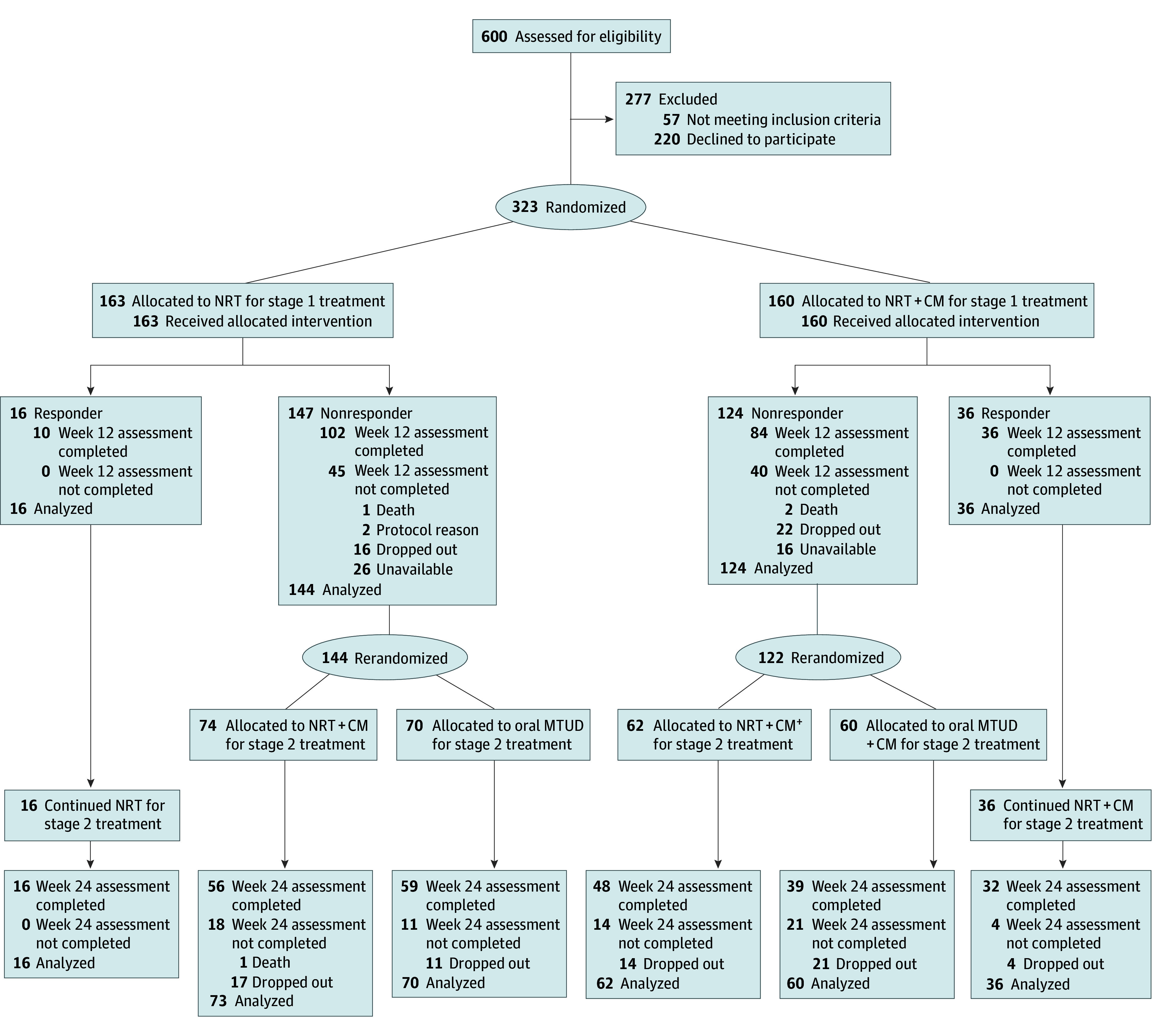
Flowchart of the Sequential Multiple-Assignment Randomized Trial Approach to Treating Tobacco Use Disorder in Persons With HIV The number of cigarettes per day was assessed at 12 weeks in 118 of 163 participants (72.4%) receiving nicotine replacement therapy (NRT) alone and 120 of 160 participants (75.0%) receiving NRT plus contingency management (CM). The number of cigarettes per day was assessed at 24 weeks in 98 of 130 participants (75.4%) among nonresponders who were randomized to switch to oral medications for tobacco use disorder (MTUD) and 104 of 136 participants (76.5%) among nonresponders who were randomized to CM intensified (CM^+^).

The SMARTTT randomized clinical trial involved 2 sequential randomizations.^36,37^ Participants were randomized to receive NRT alone or NRT plus CM for the first 12 weeks (stage 1). At week 12, those with confirmed smoking abstinence were classified as responders and continued their assigned initial treatment; those without confirmed smoking abstinence were classified as nonresponders and were rerandomized to either switch to oral MTUD or intensify CM (CM intensified) for the next 12 weeks (stage 2) (Figure 1). Assessments occurred at week 12 (end of stage 1 treatment) and week 24 (end of stage 2 treatment). This study was registered at ClinicalTrials.gov. Institutional review boards at Mount Sinai Icahn School of Medicine, Yale School of Medicine, SUNY Downstate, and Wayne State University approved the study (Supplement 1). Participants provided written informed consent to participate. This report follows the Consolidated Standards of Reporting Trials (CONSORT) guideline,^38,39^ with COVID-19–^40^ and varenicline recall-related modifications.^41^ The trial protocol is provided in Supplement 1.

### Study Context and Participants

The coordinating center was at Yale.^42^ HIV clinics within Yale New Haven Hospital Health System, State University of New York’s University Hospital at Downstate, and Mount Sinai Health System participated. Study entry criteria were pragmatic (eTable 1 in Supplement 2). Participants were reimbursed $50 for completion of each assessment (total $150).

### Interventions

All intervention components were designed to be delivered by residency-trained clinical pharmacists in HIV clinics during 10 visits across 24 weeks (eAppendix in Supplement 2). Medications were prescribed per guidelines^11^ and obtained from retail pharmacies. Participants were invited for follow-up visits to assess medication adherence, adverse effects, and progress toward abstinence.

#### Medications for Tobacco Use Disorder

##### NRT

During stage 1, all participants were counseled on and offered dual (long- and short-acting) NRT with brief advice. Those who responded to NRT at the end of week 12 continued NRT with dose adjustment as indicated.^43^

##### Oral MTUD

Participants who did not respond to stage 1 treatment may have been randomized to oral MTUD at stage 2. Originally designed to include only varenicline, this treatment was expanded to include bupropion given the varenicline recall.^41^ When both medications were available, clinical pharmacists were guided to offer participants both options, prioritizing varenicline given existing evidence.^44^

#### CM and CM Intensified

In this CM program, participants were rewarded for reporting past 7-day smoking abstinence, confirmed with an exhaled carbon monoxide (eCO) level of 6 ppm or less or verified by next closest informant.^45^ Rewards involved fishbowl draws and gift cards to local stores or reloadable debit cards with maximum earnings of $350 during stage 1 and $850 during stage 2. During CM, participants earned 1 draw the first time they achieved this goal, with escalation by 1 draw for continued abstinence to a maximum of 5 draws per visit. During CM intensified, potential draws started at 5 with a maximum of 8 draws per visit on continued abstinence. For participants who were randomized to NRT alone in stage 1 and then rerandomized to CM intensified in stage 2, they received CM; for participants who were randomized to NRT plus CM in stage 1 and then rerandomized to CM intensified in stage 2, they received CM intensified.

#### Determining Treatment Response: Week 12 Tailoring Variable

Based on self-report with confirmation, stage 1 treatment response was determined at week 12. Participants lacking confirmed abstinence were classified as nonresponders.

#### Clinical Pharmacist Training

Before study launch, clinical pharmacists were trained to promote protocol fidelity. We tracked visit completion and duration across 24 weeks.

### Data Collection

Follow-up assessments occurred at weeks 12 and 24 after randomization. Assessments included self-reported measures and eCO and electronic medical record data. Self-reported race and ethnicity using predefined categories guided by the National Institutes of Health reporting requirements were collected at baseline to characterize the sample and were not analyzed otherwise. Categories included American Indian or Alaska Native, Asian, Black or African American, Hispanic or Latina/o, Native Hawaiian or Other Pacific Islander, White, and multiracial.

### Outcomes

Smoking-related measures included responses to “Have you smoked even a puff in the last 7 days?” confirmed with eCO levels of 6 ppm or lower or by next closest informant.^45^ Among participants who reported smoking, we asked, “How many cigarettes a day do you smoke?” to assess mean cigarettes per day (CPD). With each visit, we tracked medications prescribed. Adverse events were classified by study relatedness and severity.

### Randomization and Blinding

Participants were randomized 1:1 in stage 1 to NRT vs NRT plus CM stratified by site and Heaviness of Smoking Index (HSI). Nonresponders at week 12 were rerandomized 1:1 to switch to oral MTUD or intensify CM (ie, CM or CM intensified); rerandomization was stratified by site and first-stage treatment. Randomization procedures were implemented using REDCap.^46^ Blinding of participants, clinical pharmacists, research coordinators, and investigators to assignment was not possible because of the nature of the intervention and staffing logistics. However, during stage 1, participants, clinical pharmacists, research coordinators, and investigators were blinded to the stage 2 randomized assignment for participants with nonresponse at week 12.

### Sample Size Determination and Statistical Plan

The COVID-19 pandemic impacted the study. Originally, the primary end point at weeks 12 and 24 was biochemically verified abstinence with a target of 632 participants. Due to the onset of the pandemic in 2020, study initiation was delayed and activities were intermittently interrupted with subsequent COVID-19 peaks.^47^ Because of the impact of the pandemic on recruitment and because smoking reduction is clinically meaningful,^3^ we changed the primary outcome to CPD for stage 1 (12 weeks) and stage 2 (24 weeks) comparisons to assure adequate power per discussions with the Data and Safety Monitoring Board and funder.^48^ Based on our recruitment experience, we projected a sample size of 320. Given our observed unavailability of participants for follow-up of 20% and a stage 1 nonresponse rate of 88.5%, we expected we would have 80% power to detect a difference of 2.7 CPD for comparison of stage 1 interventions and 2.9 CPD for comparison of stage 2 interventions at the .025 2-sided (ie, Bonferroni-corrected) statistical significance level.

### Statistical Analysis

Analyses were conducted using intention-to-treat principles. Per the strategy by Fitzmaurice et al,^49^ we used a repeated measures linear mixed model that conditions on baseline CPD by including both baseline and 12-week CPD as outcomes. Given that the groups were randomized, we constrained the mean CPD to be equal between treatment groups at baseline. This was accomplished by recoding treatment (NRT, NRT plus CM) and time (baseline, 12 weeks) into a single 3-level variable (ie, 0 at baseline, 1 = NRT at 12 weeks, 2 = NRT plus CM at 12 weeks). This was similarly done for the 24-week comparison in a separate model. We adjusted for site, age, HSI, and sex. A random intercept for participant was included to account for within-subject correlation. Comparison of NRT to NRT plus CM at 12 weeks included all participants randomized in stage 1, while comparison of switching vs intensifying in terms of week 24 CPD included participants who did not respond and were therefore rerandomized at week 12. Both primary outcome comparisons were made at the Bonferroni-corrected .025 2-sided significance level. Multivariable logistic regression with adjustment for the same covariates as in primary analyses was used to evaluate abstinence at 12 and 24 weeks. Estimated proportions and odds ratios with appropriate CIs are presented.

For both CPD and abstinence outcomes at 24 weeks, we also evaluated the overall best strategy. There were 4 embedded ATS in this SMART: (1) NRT initially followed by switching to oral MTUD for nonresponders at week 12 and continuing NRT for responders; (2) NRT initially followed by adding CM (NRT plus CM) for nonresponders at week 12 and continuing NRT for responders; (3) NRT plus CM initially followed by switching to oral MTUD (MTUD plus CM) for nonresponders at week 12 and continuing NRT plus CM for responders; and (4) NRT plus CM initially followed by intensifying CM (NRT plus CM intensified) for nonresponders at week 12 and continuing NRT plus CM for responders. To compare outcomes across adaptive interventions, we used the replication and weighting method to ensure that both responders and nonresponders were appropriately represented.^50^ To accommodate the sequential 2-stage design of SMART, we employed a piecewise linear specification, allowing for outcome trajectories to vary across intervention stages with a transition point at the second randomization.^51^ We then applied generalized estimating equations to model outcomes over time, including covariates for treatment assignment at stage 1 and stage 2, time since stage 1 and stage 2 randomization, their interaction, site, age, HSI, and sex. For the continuous outcome of CPD, we specified a normal distribution with an identity link; for binary abstinence, we specified a binomial distribution with a logit link. Robust standard errors were used to provide valid inference. Estimated mean CPD and estimated probabilities of abstinence, with associated 95% CIs, were calculated for each of the strategies at week 12 and week 24.

For all aforementioned analyses, missing data were addressed using multiple imputation with 10 imputed datasets, generated under fully conditional specification methods for SMART designs.^52^ Results were combined across imputations using the Rubin rules. We used descriptive statistics to characterize visit completion, prescription for MTUD, CM earnings, and adverse events. All analyses were conducted in SAS, version 9.4 (SAS Institute Inc).

## Results

### Baseline Characteristics and Participant Flow

From July 27, 2020, through March 28, 2024, 323 participants (142 [44.0%] female and 181 [56.0%] male at birth; mean (SD) age, 55.0 [10.7] years) who smoked a mean (SD) of 12.8 (7.2) CPD were randomized in stage 1 to NRT alone (n = 163) or NRT plus CM (n = 160). In total, 8 of 317 participants (2.5%) self-reported being American Indian or Alaska Native, 230 of 317 (72.6%) being Black or African American, 74 of 322 (23.0%) being Hispanic or Latina/o, 62 of 317 (19.6%) being White, and 15 of 317 (4.7%) being mixed race. On completion of stage 1, 266 participants (82.4%) were rerandomized in stage 2 (130 of 323 [40.2%] were rerandomized to oral MTUD and 136 of 323 participants [42.1%] were rerandomized to intensify); 52 participants with confirmed abstinence continued their treatment (Figure 1). Baseline characteristics of participants did not differ between groups (Table 1). eTable 1 in Supplement 2 provides baseline characteristics by stage 2 treatment.

**Table 1.** Baseline Characteristics of SMARTTT Study Participants^a^

Characteristic	Participants	
	NRT alone (n = 163)	NRT + CM (n = 160)
Sociodemographic		
Age, mean (SD), y	55.7 (10.8)	54.4 (10.6)
Sex at birth		
Female	71/163 (43.6)	71/160 (44.4)
Male	92/163 (56.4)	89/160 (55.6)
Sex at baseline		
Female	69/163 (42.3)	71/159 (44.7)
Male	92/163 (56.4)	87/159 (54.7)
Transgender	2/163 (1.23)	1/159 (0.6)
Race and ethnicity, No./total No. (%)		
American Indian or Alaska Native	5/159 (3.1)	3/158 (1.9)
Asian	NR^b^	NR^b^
Black or African American	116/159 (73.0)	114/158 (72.2)
Hispanic or Latina/o	42/160 (26.3)	32/162 (19.8)
Native Hawaiian or Other Pacific Islander	NR^b^	NR^b^
White	29/159 (18.2)	33/158 (20.9)
Multiracial	9/159 (5.7)	6/158 (3.8)
Sexuality, No./total No. (%)		
Lesbian or gay	31/162 (19.1)	28/158 (17.7)
Straight	112/162 (69.1)	110/158 (69.6)
Bisexual	13/152 (8.0)	14/158 (8.9)
Something else	6/162 (3.7)	6/158 (3.8)
Marital status, No./total No. (%)		
Married or in a civil union or domestic partnership	31/163 (19.0)	22/160 (13.8)
Divorced	20/163 (12.3)	27/160 (16.9)
Separated	12/163 (7.4)	14/160 (8.8)
Widowed	11/163 (6.8)	10/160 (6.3)
Never married	89/163 (54.6)	87/160 (54.4)
Housing insecurity, past 12 mo, No./total No. (%)	24/163 (14.7)	25/160 (15.6)
Food insecurity, past 12 mo, No./total No. (%)	29/163 (18.0)	29/160 (18.1)
Medication insecurity, past 12 mo, No./total No. (%)	11/163 (7.2)	9/160 (5.9)
Currently employed, No./total No. (%)	24/159 (15.1)	33/155 (21.3)
Highest level of education, No./total No. (%)		
Elementary school	8/163 (4.9)	4/159 (2.5)
Some high school	32/163 (19.6)	41/159 (25.8)
High school graduate	53/163 (32.5)	57/159 (35.9)
Some college or technical school	51/163 (31.3)	39/159 (24.5)
College graduate	19/163 (11.7)	18/159 (11.3)
Have any health insurance, No./total No. (%)	162/162 (100)	159/159 (100)
Public (Medicaid, Medicare, other public), No./total No. (%)^c^		
Medicaid	130/161 (80.8)	134/159 (84.3)
Medicare	60/159 (37.7)	52/159 (32.9)
Other public insurance	29/161 (18.0)	38/158 (24.1)
Private	23/161 (14.3)	13/157 (8.3)
Ryan White HIV/AIDS program or AIDS Drug Assistance Program, No./total No. (%)	35/160 (21.9)	33/157 (21.0)
Mode of usual transportation, No./total No. (%)		
Drive self	23/154 (14.9)	21/153 (13.7)
Someone else drives	21/154 (13.6)	14/153 (9.2)
Clinic van	26/154 (16.9)	14/153 (9.2)
Bus	38/154 (24.7)	32/153 (20.9)
Subway	24/154 (15.6)	32/153 (20.9)
Walk	12/154 (7.8)	25/153 (16.3)
Other (eg, medical car service, taxi, bike, scooter)	10/154 (6.5)	15/153 (9.8)
Smoking characteristics, No./total No. (%)		
Smoking pattern		
Every day	163/163 (100)	158/160 (98.8)
Some days	0/163	2/160 (1.2)
eCO level, median (IQR), ppm	10.0 (6.0-16.0)	10.0 (6.0-61.0)
Cigarettes per day, median (IQR)	10.0 (8.0-20.0)	10.0 (7.0-17.5)
HSI, median (IQR), total score^d^	3.0 (2.0-4.0)	3.0 (2.0-4.0)
Readiness to quit score, median (IQR)^e^	9.0 (7.0-10.0)	8.5 (7.0-10.0)
Relapse risk score^f^	7.0 (5.0-8.0)	7.0 (5.0-8.0)
Type of cigarettes usually smoked		
Menthol	138/163 (84.7)	133 (83.1)
Nonmenthol	20/163 (12.3)	23 (14.4)
No usual type	5/163 (3.1)	4 (2.5)
E-cigarette or other electronic vaping products use, No./total No. (%)		
Every day	4/162 (2.5)	5/159 (3.1)
Some days	10/162 (16.1)	8/159 (5.0)
Prior use, not at all currently	29/162 (17.9)	21/159 (13.2)
Never used	119/162 (73.5)	125/159 (78.6)
Nonmedical substance use, past 3 mo, No./total No. (%)^g^		
Alcohol use		
Low risk	142/163 (87.1)	142/160 (88.8)
Medium risk	14 /163(8.6)	13/160 (8.1)
High risk	7/163 (4.3)	5/160 (3.1)
Cannabis use		
Low risk	113/163 (69.3)	116/160 (72.5)
Medium risk	43/163 (26.4)	40/160 (25.0)
High risk	7/163 (4.3)	4/160 (2.5)
Stimulant use		
Low risk	148/163 (90.8)	143/160 (89.4)
Medium risk	5/163 (3.1)	11/160 (6.9)
High risk	10/163 (6.1)	6/160 (3.8)
Sedative or sleeping medication use		
Low risk	157/163 (96.3)	155/160 (96.9)
Medium risk	6/163 (3.7)	4/160 (2.5)
High risk	0/163 (0)	1/160 (0.6)
Opioid use		
Low risk	162/162 (99.4)	158/160 (98.8)
Medium risk	1/162 (0.6)	2/160 (1.3)
High risk	0/162 (0)	0/160 (0)
Depressive symptoms, moderate to severe, No./total No. (%)^h^	32/161 (19.9)	30/155 (19.4)
HIV-related measures, No./total No. (%)		
Prescribed antiretroviral therapy	160/163 (98.2)	154/160 (96.3)
HIV viral load, detectable	19/161 (11.8)	17/158 (10.8)
CD4 cell count, median (IQR), cells/mm^3^	600.0 (328.0-875.0)	596.5 (356.0-855.5)
VACS Index 2.0 score^i^	45.0 (31.0-58.0)	42.0 (33.0-53.0)
Location, No./total No. (%)		
Brooklyn, New York	43/163 (26.4)	41/160 (25.6)
Manhattan, New York	70/163 (42.9)	70/160 (43.8)
New Haven, Connecticut	46/163 (28.2)	45/10 (28.2)
Bridgeport, Connecticut	4/163 (2.5)	4 (2.5)

Abbreviations: CM, contingency management; eCO, exhaled carbon monoxide; HSI, Heaviness of Smoking Index; NR, not reported; NRT, nicotine replacement therapy; SMARTTT, SMART [sequential multiple-assignment randomized trial] Approach to Treating Tobacco Use Disorder in Persons With HIV; VACS, Veterans Aging Cohort Study.

^a^ Additional demographic information by stage 2 randomization groups can be found in eTable 1 in Supplement 2.

^b^ Not reported to avoid potential identification of participants as there were only a few participants in this category.

^c^ Not mutually exclusive categories.

^d^ Based on Heaviness of Smoking Index (scores range from 0-6, with 0-2 indicating low addiction; 3-4, moderate addiction; and 5-6, high addiction).

^e^ Based on a scale from 1 to 10, where 1 indicates not ready and 10 indicates ready.

^f^ Based on the Wisconsin Predicting Patient’s Relapse score; items are summed to create a total score, ranging from 1 to 13, with higher scores indicating a higher likelihood of smoking relapse.

^g^ Based on the Alcohol, Smoking and Substance Involvement Screening Test-Lite score, ranges from 0 to 4 for alcohol and 0 to 3 for other substances, with higher numbers associated with higher risk.

^h^ Based on the 8-item Personal Health Questionnaire score higher than 9; scores range from 0 to 28, with higher than 9 consistent with at least moderate depressive symptoms.

^i^ VACS Index scores typically range from 0 to 129, with higher numbers indicating higher 5-year mortality risk.

### Outcomes and Estimations

#### Smoking Outcomes at End of Stage 1

After stage 1 (week 12), both groups decreased CPD from baseline, but there was no difference between the NRT plus CM (least-squares mean [LSM], 4.9 [97.5% CI, 3.5-6.2] CPD) and NRT only (LSM, 5.2 [97.5% CI, 3.9-6.5] CPD) groups in CPD (adjusted LSM difference, −0.3 [97.5% CI, −1.9 to 1.3] CPD; *P* = .66) (Table 2). Confirmed abstinence was greater at 12 weeks in the NRT plus CM group (36 of 160 [22.5%]) compared with the NRT only group (16 of 163 [9.8%]) (adjusted odds ratio [AOR], 1.52 [99.0% CI, 0.44-4.24]; *P* = .002).

**Table 2.** Stage 1, Week 12 Treatment Outcomes

Outcomes	NRT (n = 163)	NRT+CM (n = 160)	Adjusted treatment effect	*P* value
Cigarettes per day, LSM (97.5% CI)^a^	5.2 (3.9-6.5)	4.9 (3.5-6.2)	Difference = −0.3 (−1.9 to 1.3)	.66
Confirmed abstinence, % (99% CI)				
Multiple imputation^b^	12.9 (5.6-27.1)	28.6 (14.9-47.9)	Odds ratio = 2.7 (1.2 to 6.1)	.002
Imputation as nonabstinent^c^	8.1 (3.3-18.7)	20.1 (9.8-36.8)	Odds ratio = 2.8 (1.2 to 6.7)	.002

Abbreviations: CM, contingency management; LSM, least-squares mean; NRT, nicotine replacement therapy.

^a^ Results from multivariable mixed model with multiple imputation with adjustment for site, age, Heaviness of Smoking Index, and sex; statistical significance evaluated at the *P* < .025 level.

^b^ Results from multivariable logistic model with adjustment for site, age, Heaviness of Smoking Index, and sex with multiple imputation; statistical significance evaluated at the *P* < .01 level.

^c^ Results from multivariable logistic model with adjustment for site, age, Heaviness of Smoking Index, and sex and assuming nonabstinence for those with missing data; statistical significance evaluated at the *P* < .01 level.

#### Smoking Outcomes at End of Stage 2 Among Participants Without Stage 1 Response

Among participants who did not respond to stage 1 treatment, CPD at week 24 was lower among those with intensified CM compared with those who switched medications (eg, LSM, 3.0 [97.5% CI, 1.3-4.8] CPD for intensifying vs stage 1 initial NRT switching medication (LSM, 6.8 [97.5% CI, 5.1-8.5] CPD) (Table 3). However, the benefit of intensifying was dependent on first-stage treatment and only significant among those initially receiving NRT alone (adjusted LSM difference, −3.8 [97.5% CI, −6.0 to −1.5] CPD; *P* < .001 vs 0.3 [97.5% CI, −2.1 to 2.6] CPD for initially receiving NRT plus CM; *P* = .80; *P* = .005 for interaction by stage 1). Confirmed abstinence was not statistically different in the switch to oral MTUD group vs intensified CM group regardless of stage 1 treatment (AOR, 1.52 [99% CI, 0.44-4.24]; *P* = .37; *P* = .85 for interaction).

**Table 3.** Stage 2, Week 24 Treatment Outcomes Among Participants Without Week 12 Confirmed Abstinence^a^

Outcomes	Stage 1 treatment	Stage 2 switch (n = 130)	Stage 2 intensified (n = 136)	Adjusted treatment effect	*P* value	*P* value for interaction by stage 1
Cigarettes per day, LSM (97.5% CI)^b^	Total	5.8 (4.4 to 7.2)	4.0 (2.7 to 5.4)	−1.7 (−3.4 to −0.1)	.02	.005
	Stage 1 NRT	6.8 (5.1 to 8.5)	3.0 (1.3 to 4.8)	−3.8 (−6.0 to −1.5)	<.001	
	Stage 1 NRT + CM	4.8 (2.9 to 6.7)	5.1 (3.2 to 6.9)	0.3 (−2.1 to 2.6)	.80	
Confirmed abstinence, % (99% CI)^c^	Total	11.0 (2.3 to 38.6)	15.7 (5.2 to 38.9)	1.5 (0.4 to 5.2)	.37	.85
	Stage 1 NRT	10.1 (2.1 to 36.9)	13.7 (4.2 to 36.6)	1.4 (0.3 to 7.3)	.58	
	Stage 1 NRT + CM	11.7 (1.6 to 51.8)	18.0 (4.1 to 52.7)	1.7 (0.3 to 8.8)	.43	

Abbreviations: CM, contingency management; LSM, least-squares mean; NRT, nicotine replacement therapy.

^a^ Stage 1 NRT plus stage 2 switch, n = 70; stage 1 NRT plus stage 2 intensified, n = 74; stage 1 NRT plus CM plus stage 2 switch, n = 60; stage 1 NRT plus CM plus stage 2 intensified, n = 62.

^b^ Results from multivariable mixed model with multiple imputation with adjustment for site, age, Heaviness of Smoking Index, and sex; statistical significance evaluated at the *P* < .025 level.

^c^ Results from multivariable logistic model with multiple imputation with adjustment for site, age, Heaviness of Smoking Index, and sex; statistical significance evaluated at the *P* < .01 level.

#### Smoking Outcomes Based on Overall Treatment Strategies

**Figure 2.**
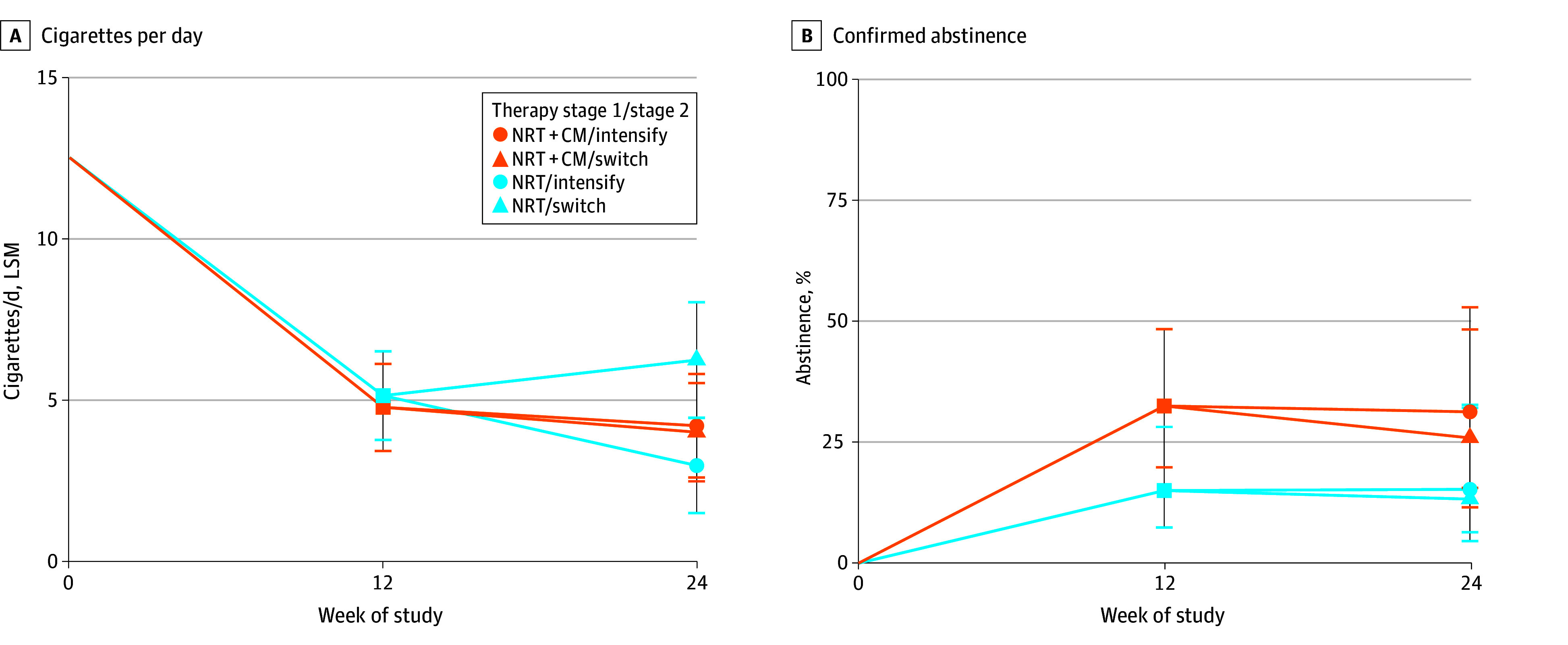
Cigarettes Per Day and Confirmed Abstinence by Adaptative Strategy A, Cigarettes per day based on multivariable models with multiple imputation (n = 323). B, Confirmed abstinence based on multivariable models with multiple imputation adjusted for site, age, Heaviness of Smoking Index, and sex (n = 323). CM indicates contingency management; LSM, least-squares mean; and NRT, nicotine replacement therapy.

Overall, 24-week CPD was lowest for the adaptive intervention of NRT alone followed by NRT plus CM (eg, LSM, 3.0 [99% CI, 1.5-4.4] CPD vs 4.2 [99% CI, 2.6-5.8] CPD for NRT plus CM followed by NRT plus CM intensified) (Figure 2A; eTable 2 in Supplement 2). Week 24 abstinence was greatest in the NRT plus CM followed by NRT plus CM intensified group (eg, LSM, 30.0% [99% CI, 16.2%-48.7%] vs 12.8% [99% CI, 5.2%-28.3%] for NRT followed by NRT plus CM intensified), although this did not significantly differ from other strategies (Figure 2B).

### Visit Completion, Prescription for MTUD, CM Earnings, and Adverse Events

Visit completion rates varied by stage and weeks in study (eFigure 1 in Supplement 2). Participants most consistently received short-acting NRT, with less consistent receipt of oral MTUD (eFigure 2 in Supplement 2). Participants randomized to CM for stage 1 treatment earned a mean (SD) of $34.9 ($76.2). Participants with response at week 12 additionally earned a mean (SD) of $374.2 ($228.9) during stage 2; participants without response at week 12 and rerandomized to intensify or switch treatment additionally earned a mean (SD) of $57.1 ($152.1) during stage 2. Visits involving CM lasted a mean of 14-18 (range, 5-60) minutes. No serious adverse events were considered possibly or probably protocol-related (eTable 3 in Supplement 2).

## Discussion

In this randomized clinical trial of people with HIV who smoked, we generated several new insights to inform TUD treatment pathways for this population. First, our results indicated that adding CM to NRT initially did not yield significantly lower CPD but promoted abstinence at 12 weeks. Second, among people with HIV who did not confirm abstinence after 12 weeks of treatment, the addition of CM promoted lower CPD, particularly among those who started with NRT alone. Third, we found that the optimal ATS differed depending on the goal (ie, harm reduction by reducing CPD or abstinence). To achieve the lowest CPD, our findings supported (1) beginning treatment with NRT alone; (2) for individuals who responded, continuing NRT alone; and (3) for individuals who did not respond to NRT, adding CM. Across all groups, we observed notable and clinically meaningful reductions in CPD from baseline. On the other hand, when the goal was abstinence, our findings supported (1) beginning treatment with NRT plus CM; (2) for individuals who responded, continuing NRT plus CM; and (3) for individuals who did not respond to NRT plus CM, intensifying CM (ie, CM intensified). Given the overall low levels of CPD and high rates of abstinence achieved during the intervention period, our findings yielded support for clinical pharmacist–delivered TUD treatment involving CM integrated into HIV clinics.

Our findings are notable, given limitations of existing interventions to address TUD among people with HIV.^13^ We do recognize that CM is not yet widely available. However, the Centers for Medicare and Medicaid Services (CMS) recently endorsed CM use for TUD and other substance use disorders,^53^ and CMS has issued Section 1115 Demonstration Waivers for CM programs to treat individuals receiving Medicaid with opioid and stimulant use disorders. In the future, CM may be a routinely reimbursable TUD treatment with models (eg, length, rewards) aligned with ours if programs can demonstrate budget-neutral effects to the federal government and health benefits.^54^

A finding of particular interest is that tobacco abstinence was improved in the first stage by the CM intervention, while CPD was improved in the second stage. This finding may simply be a stochastic phenomenon, or a consequence of the reduced power to detect abstinence caused by the reduced sample size. A third explanation is that individuals highly motivated to quit smoking were able to do so during the first stage of the intervention, resulting in the finding of enhanced abstinence in the NRT plus CM group. A fourth explanation is that our findings may relate to the design of our CM program, which was specifically designed to target abstinence. Lastly, there was inconsistent oral MTUD prescribing. Clinical pharmacists received training on MTUD with ongoing supervision and fidelity monitoring to promote consistent prescribing per protocol. However, they were instructed to not prescribe the medication if the participant declined it^37^ and to continue the discussion during subsequent visits. In addition, the varenicline medication recall disrupted access during the study and may have heightened concerns about risks. Study of factors driving MTUD underprescribing are needed. Future studies should focus on tailoring tobacco treatment interventions in HIV settings by considering adverse social determinants of health, identifying treatment goals (eg, abstinence vs reduction), providing more flexible reward programs, including other verifiable targets to address TUD, and facilitating enhanced patient choice regarding MTUD to engage people with HIV quickly and consistently.

### Limitations

This study has limitations. First, the pandemic affected the study considerably. Enrollment was delayed for nearly 1 year. Hence, we reduced the sample size as noted, with a newer focus on the harm reduction end point of change in daily cigarette consumption. Although we still found favorable and significant results, we did not power the study to detect improved abstinence in stage 2 treatment, and comparison of the adaptive interventions was exploratory and not planned to detect differences. Second, participants were provided medication prescriptions, not the actual medication, and we observed access issues.^55^ In addition, prescription of oral MTUD was lower than expected, particularly among participants initially randomized to receive NRT alone. Our findings reflected experiences with intention to treat by offering MTUD and our pragmatic design,^56^ enhanced clinical practice relevance, and highlighted a need for more research to understand the multilevel factors that drove these findings. Third, we did not assess durability of effects after treatment ended. Fourth, in circumstances in which it was not possible to collect an eCO level, smoking abstinence was confirmed by next closest informant, which is subject to bias, particularly in a CM study. Fifth, our study was conducted in urban, northeastern US–based clinics, potentially limiting generalizability. In addition, clinical pharmacists are not uniformly embedded in HIV clinics; models including nonclinical pharmacist prescribers with support of other team members may offer an alternative structure as clinical pharmacists’ role in HIV care expands.^57^

## Conclusions

An ATS consisting of clinical pharmacist–delivered MTUD and CM showed effectiveness for addressing TUD among people with HIV. CM shows promise as an adjunct TUD treatment among people with HIV. Future research should focus on optimizing CM targets and schedules and medication flexibility, exploring moderators of treatment response (eg, MTUD adherence), conducting detailed implementation fidelity evaluation, and identifying more sustainable team-based treatment models.

## Notes

Supplement 1.
Trial Protocol

Supplement 2. **eTable 1.** Baseline characteristics of participants by stage 2 treatment **eTable 2.** Effects of adaptive strategies on cigarettes per day and abstinence at week 12 and week 24 (strategies n=323) **eTable 3.** Number of participants experiencing adverse events in stage 1 and stage 2 treatment by group **eFigure 1.** Clinical pharmacist visit completion by stage and treatment group eFigure 2. MTUD prescription at first visit by stage, treatment group, and medication **eAppendix.** Medications with contingency management for treating tobacco use disorder in persons with HIV—Trial intervention training manual

Supplement 3.
Data Sharing Statement
