# Supplementary material for: Clinical Pharmacists, Medications, and Contingency Management for Targeting Smoking in HIV Clinics: A Randomized Clinical Trial
Source: JAMA Netw Open. 2026 Feb 27;9(2):e2560593. doi: 10.1001/jamanetworkopen.2025.60593 (PMC12949440; doi:10.1001/jamanetworkopen.2025.60593)
Supplement: Supplement 1. — Trial Protocol [file jamanetwopen-e2560593-s001.pdf]

Mount Sinai	Protocol Name:	A SMART Approach to Treating Tobacco Use Disorder in Persons with HIV (SMARTTT)
	Principal Investigator:	Keith Sigel
	Primary Contact Name/Contact Info:	Nadia Zubair/nadia.zubair@mountsinai.org/212-824-7972
	Date Revised:	5/7/2023
	Study Number:	19-01121

HRP-503 Application (Protocol Supplement)

- *This application can only be used in conjunction with a protocol. If this project does not have a protocol from the sponsor or is already included in a grant application then a comprehensive protocol should be developed. A comprehensive template and online wizard is located at: NIH Wizard.*
- *Note that, depending on the nature of your research, certain questions, directions, or entire sections below may not be applicable, or may have been fully covered in the protocol. Provide information if and when applicable. If the answer is found in the protocol please provide a page reference. If the question is not applicable to the study, mark the section "N/A". Do not delete any sections.*
- *Be sure to complete any supplement questions from one or another ancillary office that you receive during the RUTH application process. Please make certain that the protocol, this 503 application and responses to ancillary offices do not contradict each other and the information is incorporated in all documents where appropriate. Be sure to save the Ancillary office responses you provided within RedCap and upload them to Ruth.*
- *Throughout this application are references to checklists. These tools are used by the IRB to make specific regulatory findings. To allow us to do that it is the applicant's responsibility to ensure that your protocol has sufficiently addressed these additional regulatory criteria for approval, and that the applicant identifies those protocol specific findings required by the checklist. how will they do that, here or a separate form?*
- *Keep an electronic copy of this version of the document. You will need to modify this copy when making changes.*

1. Setting of the Human Research:

- *Where within the Mount Sinai health system or its affiliates will research activities take place including subject recruitment?*

Potentially eligible patients may be recruited via multiple pathways:

1. Direct referral by clinic provider or staff;
2. Self-referral based on recruitment flyer, peer, or otherwise;
3. Proactive, opt-out recruitment based on electronic medical record review, followed by mailed letter and, if the patient does not opt out of being called, research team outreach; and/or
4. Messaging through the secure medical record with invitation to participate as available at a given site and per site policies.

Individuals who meet inclusion and exclusion criteria and provide written consent to participate will have baseline assessments performed, and then will be randomized 1:1 to either NRT or NRT+CM.

For aim 3, all clinicians, staff, clinical leadership, social workers, psychologists, and care coordinators employed at the participating site will be invited to complete the brief surveys pre- and post- trial intervention. The pre-trial survey will occur within the first 3 months of the RCT launch; the post trial survey will occur within the last three months of the RCT completion.

- *If there are any differences in the recruitment or study procedures between the sites please highlight them here.*

For the Yale New Haven Hospital site only (Aims 1 and 2):

To assist in recruitment, the Yale Clinical Center for Investigation (YCCI) will help to identify potential eligible participants. The YCCI team will mail recruitment letters and post social media advertisements. In addition, they will send announcements through the Help Us Discover database, which contains several thousand individuals who volunteer for clinical research. Additionally, they will send messages through the secure messaging system through the medical record (MyChart via Epic) for those patients who have opted in to being contacted for clinical research studies.

2. Resources Available to Conduct the Human Research: (the aim here is to assess if the research is likely to be successful and thus justify the efforts and risks taken by the subjects):

- *Explain the feasibility of meeting the recruitment goals of this project, and demonstrate (e.g., based on retrospective data) a potential for recruiting the required number of suitable subjects within the agreed recruitment period. (For example, how many potential subjects do you have access to? What percentage of those potential subjects do you need to recruit? If this has been reviewed by a committee for recruitment feasibility [e.g. PR&MC], please indicate so.)*
- *For research involving considerable data extraction or mining describe who will be providing those services.*
- *For research conducted outside MSSM and its research affiliates, describe the facilities used for conducting the research.*
- *Describe your process to ensure that all persons assisting with the trial are adequately informed about the protocol, the investigational product(s), and their trial-related duties and functions. If research is being carried out in a clinical setting explain how the clinical staff will be informed of the trial and their involvement, e.g. medication administration. If nursing or other services have reviewed and approved this project please upload the documentation.*

Preliminary data on patients receiving care at the participating sites, using ICD-10 codes and prescription data for NRT and VAR in a one-year time period are listed in the table below:

Table 1. Characteristics of PLWH engaged in care at participating sites

Characteristic	Haelen Center	Nathan Smith Clinic	STAR Health Center	Mount Sinai*
**Total in care, n**	773	725	1208	8,709
**Tobacco use disorder, n**	306	319	235	2,439
Prescribed NRT, n	71	124	76	1,082
Prescribed VAR, n	40	31	24	415

*Based on available data, reflects numbers from 3 out of 5 HIV clinics at Mt. Sinai. Recruitment will occur at Jack Martin and Peter Krueger Clinics, with expansion as needed to additional sites.

Among participating clinics, we have access to over 3,300 potentially eligible patients. Recruitment of the 622 participants will occur over a 3.5 year period. We plan to enroll 64 participants in year 1, and 186 participants in each of years 2, 3, and 4. Given the large number of potential subjects and the incentives associated with study participation, we anticipate achieving enrollment targets.

The organization structure, including roles of each team member, is outlined in the chart below:

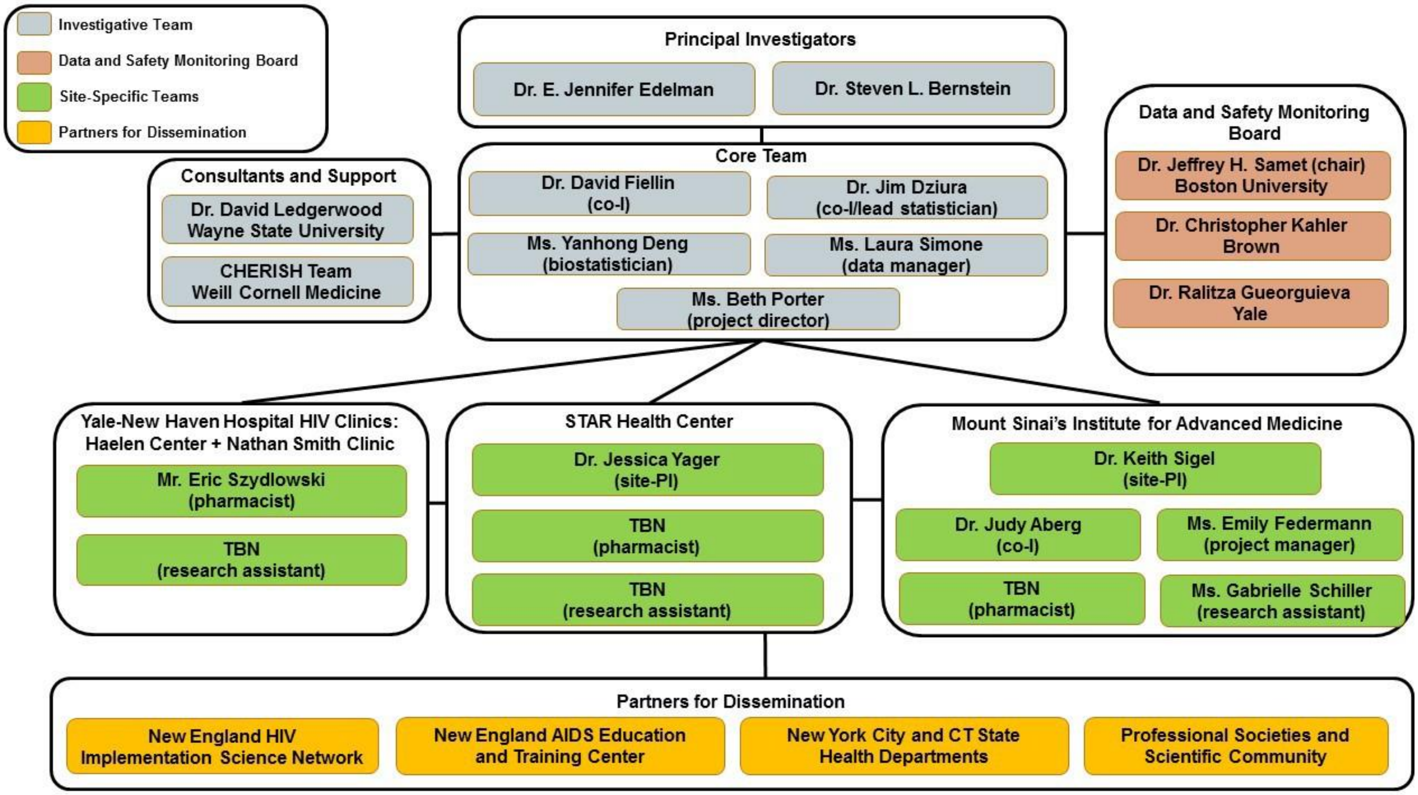

E. Jennifer Edelman, MD, is an Associate Professor of Medicine and Public Health at Yale School of Medicine and is certified as an internist, HIV specialist, and in Addiction Medicine. In addition, she serves as the physician consultant in the Addiction Treatment Program at the Nathan Smith HIV clinic. Her research over the past 10 years has focused on optimizing HIV prevention and treatment as it relates to substance use in HIV treatment settings.

Steven Bernstein, MD is Professor of Emergency Medicine and Public Health at Yale and Founding Director of the Yale Center for Implementation Science. He has extensive expertise in developing screening and treatment interventions in clinical settings (e.g. emergency departments [ED], inpatient settings) to address tobacco use among vulnerable patients, including PLWH. He is currently leading his fifth NIH-funded clinical trial, including a randomized trial using the novel Multiphase Optimization Strategy (MOST) design of four evidence-based interventions for the treatment of tobacco use disorder.

David Fiellin, MD is Professor of Medicine and Public Health and Founding Director of the Program on Addiction Medicine at Yale. He is certified in Internal and Addiction

Medicine and researches the implementation of addiction treatment services in HIV clinics, Primary Care and Emergency Departments. He directs the Clinical Health Services Research Core at the Yale Center for Interdisciplinary Research on AIDS (CIRA) and is PI on current NIDA and NIAAA-funded randomized trials focused on addressing substance use in HIV clinics.

James Dziura, PhD is Professor in the Section of Analytics and Informatics in Emergency Medicine and Department of Biostatistics School of Public Health, Deputy Directory of Yale Center for Analytical Sciences and Yale Data Coordinating Center, Co-Director of Yale CTSA Biostatistics Epidemiology and Research Design Core. Dr. Dziura is a trained epidemiologist with a research focus in the design, coordination, conduct and analysis of longitudinal studies and clinical trials. Dr. Dziura has over 14 years of experience in organizing and leading biostatistical and data management teams.

Elizabeth Porter, MBA is a Clinical Research Coordinator at Yale University with more than20 years of experience in clinical trials. She currently works closely with Drs. Edelman, Fiellin and Dziura in her role as Project Coordinator on multiple NIH-funded projects.

Yanhong Deng, MPH has more than 9 years of biostatistical experience on multiple clinical trials and works closely with Drs. Dziura, Fiellin and Edelman.

Ms. Simone is a Senior Data Manager and an expert in multi-site data coordination, developmentof data protocols and manual of procedures, and maintenance and monitoring of data quality and security. She works closely with Dr. James Dziura and Ms. Yanhong Deng.

Dr. Jessica Yager is an Assistant Professor in the Department of Medicine at SUNY-DMCand will be responsible for overall direction and implementation of the proposed project at the Brooklyn site.

Dr. Keith Sigel is an Associate Professor of Medicine at ISMMS. He is an Infectious Diseases specialist and clinical investigator with a strong interest in the long-term care of persons with HIV. He will act in a supervisory capacity in the recruitment and retention of participants within the Mt Sinai Health System.

Dr. Judith Aberg will assist Dr. Sigel in identifying and recruiting HIV patients at clinical sites within the Mount Sinai Health System. She will also assist with the dissemination of study results. She is a high-level administrator and clinician for the HIV clinics within the Mt Sinai Health System.

Ms. Nadia Zubair will act as Project Manager and will be responsible for the day-to-day implementation of the proposal at ISMMS.

Ms. Daniela Solis and Ms. Inna Lishchenko will be primarily responsible for the recruitment of subjects and collection of data under the supervision of Dr. Sigel and Ms. Zubair.

<u>Facilities outside MSSM:</u>

The Haelen Center at Yale New Haven Hospital is an outpatient clinic that serves the wider New Haven, CT community that provides comprehensive services related to HIV prevention and treatment. Services include HIV clinical care, HIV testing and counseling, and PrEP counseling and treatment. It is one of the largest clinical sites for HIV services in the state of Connecticut (about 750 patients currently in care) and utilizes a multidisciplinary team to offer quality and holistic outpatient care to its clientele. In years past, this included on-site smoking cessation services that were funded by the CT Department of Public Health. In addition to clinicians (physicians, PAs), there is an on-site nutritionist, clinical pharmacist and case manager.

The Nathan Smith Clinic at Yale-New Haven Hospital is an outpatient clinic that also serves the wider New Haven, CT community and provides comprehensive services related to HIV prevention and treatment. It is the largest clinical sites for HIV services in the state of Connecticut (about 725 patients currently in care) and provides comprehensive care to patients living with HIV. On-site specialty services include psychiatry, neurology, women's health and addiction treatment (by Dr. Edelman). In addition to clinicians (physicians, infectious disease fellows, medical residents, a nurse practitioner, and a PA), there is an on-site clinical pharmacist, social workers, and case manager.

The **STAR Health Center (SHC) at SUNY Downstate Medical Center** offers specialized care for HIV disease, hepatitis C and substance abuse by health care professionals who are experienced in meeting the medical, social and behavioral health needs of our community. The SHC is a NYS licensed Diagnostic and Treatment Center under Article 28 of the NYSDOH and a NYS Designated AIDS Center (DAC). The SHC includes 8 exam rooms, 4 counseling rooms, 1 nursing station, space for 4 case managers,

as well as a large waiting room and conference room which can both be used for educational purposes or support groups.

<u>Training:</u>

Before patients are enrolled, we plan to conduct a full day of training for all study staff at the Coordinating Center in New Haven. In addition, Dr Edelman, Dr. Bernstein, and Ms. Porter will make annual visits to each site to monitor study-related activities which include recruitment and retention, data collection and quality and adherence to regulatory requirements. All study staff will be required to undergo all institutional-specific regulatory trainings prior to beginning the project and will also be required to update this training annually. We will develop a Contingency Management study manual to ensure consistency across sites in the delivery of the intervention.

Training and supervision of study interventionists will employ structured encounter forms and ongoing supervision to support treatment fidelity and avoid intervention drift, as is our approach in other trials. Initial training will occur at a 1 day, in-person session in New Haven with follow-up by videoconference. Our team has extensive experience in training. The objectives are to ensure that pharmacists can prescribe NRT and VAR, provide counseling regarding proper use and potential side effects, and deliver the CM intervention per protocol. Training on prescribing of NRT and VAR, potential side effects, and appropriate counseling with considerations for PLWH, will be provided by Drs. Bernstein and Edelman. CM training will be provided by Dr. Ledgerwood following established procedures used in other NIH-funded trials. Dr. Ledgerwood will conduct 2 half-day trainings that include didactic instruction on CM, demonstration of procedures for monitoring and tracking target behaviors, and role-play exercises. Before and after completing the CM didactic training, clinical pharmacists will complete a 20- item test that assesses knowledge about CM and behavioral therapy principles. After CM training, pharmacists will complete an additional multiple choice-test that assesses understanding of the protocol of the present study. The pharmacist must also successfully complete role-plays with Dr. Ledgerwood. The roles plays will include scenarios in which pharmacists are expected to demonstrate specific skills (e.g., describe strategies to avoid smoking; provide increasing reinforcement to a patient who met the behavioral targets for the week). Trained RAs will rate audiotapes of the role-plays using the Contingency Management Competence Scale (CMCS), developed by Dr. Ledgerwood and colleagues. Initially, raters will establish inter-rater reliability, and minimum coefficients of .80 will be required for acceptable ratings. The clinical pharmacist must obtain scores of at least 80% on relevant objective tests and minimum average scores of 4/7 on the role-play tests before being eligible to treat study participants.

To ensure ongoing fidelity to CM procedures, RAs will rate at least 10% of all audio-recorded sessions (randomly selected) for fidelity using the CMCS observational rating system. If the clinical pharmacists' ratings fall below a minimum average score of 4/7, or if there are low scores on any specific aspect of CM (e.g., he/she never assesses desire for prizes), we will conduct additional training until his/her ratings have reached an acceptable level.

3. Study Design:

a) Recruitment Methods (see PPHS policy):

- *Describe the source of potential subjects.*

For aims 1 and 2, potential participants will be identified by chart review or via data reports filtered on eligibility criteria, in advance of the clinic. We will develop recruitment flyers specific which have been attached. Potential participants may be referred to the study team by a clinic provider or staff member. In addition, eligible patients may be informed about the study by messaging via medical record as applicable at a given site (e.g., Epic MyChart).

- *Describe the methods that will be used to identify potential subjects (e.g. ResearchMatch.org, social media, texting, etc.).*

Potentially eligible patients may be recruited via multiple pathways:

1. Direct referral by clinic provider or staff;
2. Self-referral based on recruitment flyer, peer, or otherwise;
3. Proactive, opt-out recruitment based on electronic medical record review, followed by mailed letter and, if the patient does not opt out of being called, research team outreach; and/or
4. Messaging through the secure medical record with invitation to participate as available at a given site and per site policies.

Individuals who meet inclusion and exclusion criteria and provide written consent to participate will have baseline assessments performed, and then will be randomized 1:1 to either NRT or NRT+CM.

- *Describe how potential subjects will be approached, the involvement of the treating clinicians, use of letter, emails, calls, or similar, opt-out provisions, etc. Describe materials that will be used to recruit subjects. Include copies of these documents with the application. For advertisements, submit the final copy of printed advertisements. When advertisements are taped for broadcast, provide the final audio/video file. You may submit the wording of the advertisement prior to taping for pre-approval but final audio/video recording has to receive IRB approval before use.*

For patients who receive letters, if they do not opt out of being called, the research assistant will contact them via the phone, by phone call or text using HIPAA compliant Zoom Phone and determine whether the patient meets the remaining eligibility criteria. Those who meet criteria will be invited to meet with the research assistant in person, to be consented.
For patients who are given the flyer, if they express interest, the research assistant will approach and perform the in person screener. Those who are eligible will then be consented.

- *For social media, in addition to providing the content please explain how people will be selected or targeted to receive the ads. For websites used for screening or recruitment, provide details and access. Complete the INFO Sec screening questions and follow up as needed.*

  N/A

b) Inclusion and Exclusion Criteria:

- *Indicate any local changes not specified or differing from the protocol.*

No local changes, please refer to protocol

- *Describe how you will screen for eligibility and if you will need a waiver of Informed Consent or HIPAA. If you are using an outside firm to screen or data mine for subjects please provide details here.*

For aims 1 and 2, potential participants will be identified by chart review in advance of the clinic or via data reporting filtered on inclusion/exclusion criteria. We have attached a waiver of HIPPA authorization in order to screen patient charts to identify them as potentially eligible to approach for the study.
Patients will either be sent a letter and called or texted on the phone, or approached in person on the day of the clinic and provided with a flyer.
Patients who are called or texted on the phone will be further assessed for eligibility. Those who are eligible will be invited into the clinic for consenting.
Patients who receive the flyer and are interested will be screened using the in person screener. Those who are eligible will be consented.

For aim 3, all clinicians, staff, clinical leadership, social workers, psychologists, and care coordinators employed at the participating site will be invited to complete the brief surveys pre- and post- trial intervention.

Inclusion Criteria

- For aims 1 and 2: Patients will be eligible for study entry if they: 1. are HIV positive; 2. Receive care at 1 of the participating sites; 3. Are >18 years old; 4. have smoked >=100 cigarettes lifetime; 5. report smoking every day or some days; 6. smoke, on average, >=5 cigarettes per day; and 7. provide written informed consent. A HIPAA waiver will be requested to allow prescreening via patient chart review.
- For aim 3: All clinicians, staff, clinical leadership, social workers, psychologists, and care coordinators employed at the participating site will be invited to complete the brief surveys pre- and post- trial intervention. Survey data will be collected in a confidential fashion and administered through REDCap. Surveys will be distributed by email and clinicians and staff will be informed and encouraged to participate by clinical leadership. An alteration of written informed consent will be requested for Aim 3.

- *Any exclusions based on race, sex/gender, preferred language must be explained.*

(NOTE: You may not include members of vulnerable populations as subjects in your research unless you indicate this in your inclusion criteria).

Exclusion Criteria

- For aims 1 and 2: Patients will be excluded for: 1. exclusive use of non-cigarette tobacco or nicotine products, including e-cigarettes; 2. current use of NRT, VAR or buproprion (defined as use of medication in prior 7 days); 3. Self-report or urine testing confirming pregnancy, nursing, or trying to conceive; 4. Life-threatening or unstable medical, surgical, or psychiatric condition; 5. inability to provide at least 1 collateral contact for a friend or family member; 6. Living out-of-state; or 7. Inability to read or understand English or Spanish.
- For aim 3: Participants will be excluded for 1) less than 18 years of age and 2) NOT employed at the HIV clinic.

  Enrollment is restricted to 18 to 64 and 65 years of age or older, adults/patients and MSSM employees. We require that all subjects are able to understand what is being asked of them and can fully consent to participation and be able to ask questions. The clinic sites do not serve children. We are excluding pregnant women due to possible randomization to medication.

c) Number of Subjects:

- *Specify local recruitment numbers if not in the protocol*

  300

- *Indicate the total number of subjects to be accrued locally. This will be the maximum number of subjects that can sign the consent form without additional IRB approval. Please be sure to account for screen failures, drop-outs, etc. If applicable, distinguish between the number of subjects who are expected to be pre-screened, enrolled (consent obtained), randomized, and complete the research procedures (i.e., numbers of subjects excluding screen failures) and between subgroups of subjects (e.g. healthy volunteer, disease cohort).*

  300

- *If this is a multicenter study, indicate the total number of subjects to be accrued across all sites.*

  This study is multi-center totaling in three sites including Mount Sinai Hospital. The total number of subject goal is 320.

d) Study Timelines:

- *If not in the protocol, please clarify the how long an individual subject may be in the protocol, and for how long the protocol will be active at Mount Sinai.*

  Describe: Please refer to protocol

- *The duration of an individual subject's participation in the study (including follow-up).*

For aims 1 and 2, study participation will end by 24 weeks.
For aim 3, there will be a pre and post trial survey.

- *The duration anticipated to enroll all study subjects.*

Study launch was delayed due to COVID 19. For aims 1 and 2, we plan to enroll 3 participants in year 1, 101 participants in year 2, 97 participants in year 3,77 participants in year 4 and 42 participants in year 5.
For aim 3, the pre-trial survey will occur during year 1 and the post-trial survey will occur during year 5. Due to staff changes, it will not be the same staff in both years. We aim to recruit a total of 98 clinicians, staff, clinical leadership, social workers, psychologists, and care coordinators.

- *The estimated date for the investigators to complete this study (complete primary analyses)*

Within 5-10 years

e) Specimen Banking for Future Uses Not Part of This Project:

N/A

- *If storage will be occur at Sinai and elsewhere (e.g. the sponsor) be sure to answer these questions for both sets of samples as, at the very least, governance of future uses will differ.*
- *If specimens will be banked for future use, describe:*
- *Where the specimens will be stored, what access controls and security systems will be in place?*
- *How long they will be stored?*
- *How will researchers gain access to the specimens? If there is a resource utilization committee please provide a description.*
- *List the information to be stored or associated with each specimen (including how the specimens are labeled/coded).*
- *If the specimens are part of bank where frequently sharing among several or more users is contemplated then a complete set of Standard Operating Procedures for the repository should be submitted.*

f) Data Storage, Transmission and Confidentiality:

Describe the data and specimens to be sent out or received. If storage will occur at Sinai and elsewhere (e.g. the sponsor) be sure to answer these questions for both sets of samples as, at the very least, governance of future uses will differ.

As applicable, describe:

- *How will data be collected?*

  Data will be collected on computers at study sites using REDCap. REDCap data will be uploaded live to a Yale maintained university server in encrypted fashion. This is a live connection; therefore, all data are immediately stored on the secure server. The server is backed up hourly. This system is compliant with HIPAA and regulations for the collection of ePHI. Computers are encrypted with PGP software.

- *How will data be transmitted? If clinical trials software or similar is being used provide the names as well as the security/regulatory specifications it complies with (e.g. FDA)*

  Data will be collected on computers at study sites using REDCap. REDCap data will be uploaded live to a Yale maintained university server in encrypted fashion. This is a live connection; therefore, all data are immediately stored on the secure server. The server is backed up hourly. This system is compliant with HIPAA and regulations for the collection of ePHI. Computers are encrypted with PGP software.

- *How will data be stored? Provide data standards where known*

  Data will be stored in a computerized database system, REDCap. Then the REDCap data will be uploaded live to a Yale-maintained university server in encrypted fashion. This is a live connection; therefore, all data are immediately stored on the secure server. The server is backed up hourly.

  Upon completion of the study, all computerized participant datasets will be coded and stored in a password-protected study computer, to which only the MPIs and study personnel will have access. Any paper files with participant information will remain in locked files in the study office of the Project Director, until they are destroyed, after all analyses are complete and after the federal requisite waiting period (7 years) to maintain records. Any records containing PHI will be stored separately from coded data during storage, use, and transmission.

- *How long will data be stored?*

Any paper files with participant information will remain in locked files in the study office of the Project Director, until they are destroyed, after all analyses are complete and after the federal requisite waiting period (7 years) to maintain records. Data will be stored in the REDCap system for five years after data collection is completed.

- *What are the SOPs that govern data sharing?*

- *If this project is not funded by NIH will a Certificate of Confidentiality be obtained? If not, why not?*

This project is currently funded by the NIH and a certificate of confidentiality will be obtained

g) Data and Safety Monitoring Plan:

- **For projects with a Data Safety Monitoring Board/Data Safety Committee (DMSB/DMC):**
- *If not included in the protocol, attach a description of the DMC/DSMB, including the number, names (if available} and area of professional expertise of the members. The responsibilities of the DSMB/DMC must be clear as well as their powers and their degree of independence. The DSMB charter must be provided to the PPHS before the study may begin. Reports of the DMC/DSMB must be made available to the local PI and the MSSM PPHS. The report need not contain specifics of the study or data, but there should be clear statement if the study can continue as is, or requires changes or termination.*

Refer to protocol for DSMB members

The principal investigators (MPIs) will have overall and ultimate responsibility for monitoring the data, assuring protocol compliance, conducting the safety reviews. Although we have assessed all aspects of the proposed study as one of minimal risk, the potential exists for anticipated and/or unanticipated adverse events, serious or otherwise, to occur since it is not possible to predict with certainty the absolute risk in any given individual or in advance of first-hand experience with the proposed study methods. Therefore, we provide a plan for monitoring the data and safety of the proposed study. We will use the HIV Symptom Index, a 20-item validated tool that assess symptom burden, to facilitate monitoring of adverse events; any new or worsening symptoms will be reported to the site-PI immediately.

h) For other projects with greater than minimal risk a monitoring plan must be provided:

1. List the name(s) of the individual(s) at MSSM who will be responsible for data and safety monitoring of this study. For each individual, indicate their role, name, title, and department information. The Principal Investigator may be the only monitor of a study.

Keith Sigel
Principal Investigator
Associate Professor
Department of General Internal Medicine

Judith Aberg
Principal Investigator
Professor
Department of Medicine – Infectious Disease

2. If the qualifications of an individual to serve as a monitor are not contained in the PPHS application, they must be added to the DSMP either as a narrative description or as a CV.

MSSM Principal Monitor:
Indicate whether this person is the PI, a Team Member, or is Independent:
Last Name: Sigel
First Name: Keith
Academic Title: Associate Professor
Department: General Internal Medicine
Mailing Address: 1 Gustave L. Levy Place, Box 1087 New York, NY 10029
Phone: 212-825-7558
Fax:
E-mail: keith.sigel@mssm.edu

MSSM Additional Monitor:
Indicate whether this person is the PI, a Team Member, or is Independent:
Last Name: Aberg
First Name: Judith
Academic Title: Professor
Department: Department of Medicine – Infectious Disease
Mailing Address: 1 Gustave L. Levy Place, Box 1090 New York, NY 10029
Phone: 212-534-3240
Fax:
E-mail: judith.aberg@mssm.edu

3. Justify your choice of principal monitor in terms of the assessed risk to the research subject's health and wellbeing. In high risk studies when the principal monitor is independent of the study staff, indicate the individual's credentials, relationship to the PI, and the rationale for selection.

The principal investigators (MPIs) will have overall and ultimate responsibility for monitoring the data, assuring protocol compliance, conducting the safety reviews.
Both MPIs specialize in infectious disease therefore put them in good position to understand the risks of this population and treatment related needs of this population

4. List the specific items that will be monitored for safety (e.g., adverse events, subject compliance with the protocol, drop outs, etc.).

Although we have assessed all aspects of the proposed study as one of minimal risk, the potential exists for anticipated and/or unanticipated adverse events, serious or otherwise, to occur since it is not possible to predict with certainty the absolute risk in any given individual or in advance of first-hand experience with the proposed study methods. Therefore, we provide a plan for monitoring the data and safety of the proposed study. We will use the HIV Symptom Index, a 20-item validated tool that assess symptom burden, to facilitate monitoring of adverse events; any new or worsening symptoms will be reported to the site-PI immediately.

5. Indicate the frequency at which ACCUMULATED safety and data information (items listed in number 3 above and interim analysis of efficacy outcomes) will be reviewed by the monitor(s) or the Data Monitoring Committee (DMC). Although this information must be reviewed at least annually, the higher the study risks, the more frequently reviews must be scheduled.

Twice annually the DSMB will review the progress of the study and frequency of serious adverse events.

6. Where applicable, describe rules which will guide interruption or alteration of the study design.

During the review process, the MPIs will evaluate whether the study MPIs or the DSMB or the IRB have the authority to stop or suspend the study or require modifications.
The principal investigators (E. Jennifer Edelman, MD, MHS and Steven L. Bernstein, MD) will conduct a review of all adverse events upon completion of every study participant. The MPIs will evaluate the frequency and severity of the adverse events and determine if modifications to the protocol or consent form are required. Dr. Keith Sigel will be responsible for IRB approval and for monitoring day-to-day study activities at the Mount Sinai site. Dr. Jessica Yager will be responsible for study activities at STAR.

7. Where applicable, indicate dose selection procedures that will be used to minimize toxicity.

Symptoms associate with nicotine "overdose" include cold sweats, fainting, confusion, and pounding heart. Toxicity resolves in several hours. Cases of nicotine toxicity are extremely rare. In terms of the patch, nicotine continues to enter the bloodstream for several hours after removing it, as it leaches through the skin, so smoking within 12 hours of removing the patch is strongly discouraged. One of the most common side effects of the patch is a localized skin rash due to a

reaction to the adhesive on the patch or a reaction to the nicotine. The second most common side effect is sleep disturbance. This side effect is common in people who use the 24-hour patch. Nicotine can lead to vivid, colorful dreams and difficulty sleeping. For this study, the 24-hour patch will be utilized. Switching to a 16-hourpatch might alleviate this problem, but it often can result in nicotine cravings. Patients using the nicotine inhaler may experience cough.
There is also a very small risk that the participant could have an allergic reaction to the drug in any of these products. If this occurs, the participant will be asked to notify the study team at once and seek immediate medical attention.
Symptoms of an allergic reaction include rash, itching, swelling, dizziness, and trouble breathing.
VAR may cause nausea in some users, generally mitigated by taking before meals, and usually abating over time. Sleep disturbance, including vivid dreams, have been reported. Less common side effects include indigestion, abdominal pain and gas, fatigue, headache, and dry mouth. If these occur, the research participant will be asked to notify study personnel. The main risks of bupropion include increased risk of seizure; thus it should be avoided in patient with an underlying seizure disorder, eating disorder, untreated alcohol use disorder, and/or concurrent use of a monoamine oxidase inhibitor (MAOI). Common side effects may include insomnia, dry mouth, and constipation. Uncommon side effects may include rash, itching or hives. The medicine may also cause dizziness; thus individuals should avoid driving or using heavy machinery before they know how they might react. The medicine may also cause some patients to have abnormal behaviors or worsening mood or suicidal thoughts. Participants will be instructed to report any of these symptoms immediately to the research team.

8. List any specialized grading system that will be used to evaluate adverse events (e.g., National Cancer Institute Common Toxicity Criteria).

Grading System to Evaluate Adverse Events

The following scale will be used in grading the severity of adverse events noted during the study:
1. Mild adverse event
2. Moderate adverse event.
3. Severe unanticipated adverse event resulting inpatient hospitalization or prolongation of existing hospitalization, a persistent or significant disability/incapacity, or a congenital anomaly/birth defect.
4. Life-threatening adverse event
5. Fatal adverse event

9. Describe procedures that will be used to assure data accuracy and completeness.

Procedures for data collection, management, monitoring of quality, and analysis will include use of a computerized database system (REDCap) to monitor research activities, screening, enrollment, compliance with protocol and treatment interventions, completion of scheduled assessments, and data retrieval.

Data quality will be assured by: 1) extensive training/supervision of RAs in data collection; 2) preliminary review of all assessment instruments prior to data entry and checks for completeness and coding errors; and 3) error checking statistical programs. No interim looks are planned. Monthly reports will monitor accrual, randomization; data timeliness, quality, completeness, and overall event rates (e.g. abstinence). Error corrections will be documented.

10. Should a temporary or permanent suspension of your study occur, in addition to the PPHS, indicate to whom (NIH, FDA, sponsor, IRB) will you report the occurrence.

For this study, the following individuals, funding, and/or regulatory agencies will be notified: all co-Investigators listed on the protocol, the HIC, and the National Institutes of Health.

i) Withdrawal of Subjects:

- *Describe anticipated circumstances under which subjects will be withdrawn from the research without their consent.*

Subjects may be withdrawn from the research without their consent if they are deemed ineligible for the study.
Subjects may also be withdrawn from the research if they have experienced serious or related adverse events.

- *Describe any procedures for orderly termination.*

If a subject asks for complete withdrawal from the study, he or she must do so by written notification to the site PI.

- *Describe procedures that will be followed when subjects withdraw from the research, including partial withdrawal from procedures with continued data collection.*

If any subject asks to withdraw from the study, he/she will be notified that medical record data collection will continue, but any interventional procedures may cease.

4. Provisions for Research Related Harm/Injury:

- *Explain how clinically important incidental results will be handled. Examples include an abnormal lab finding or a survey answer indicating possible danger to self or others.*

  The surveys do not have any questions that would indicate possible danger. However, if a participant expresses suicidal thoughts the researcher assistant/coordinator will follow our suicide protocol by contacting the PI and/or a licensed physician for immediate evaluation. Any abnormal lab values will be reported to the participant immediately

- *Describe the availability of medical or psychological resources that subjects might need as a result of any anticipated adverse events that may be known to be associated with the Human Research.*

For any harms stemming from study procedures or assessments, there will be medical and mental health professionals available for consultation and/or referral.

- *If the research involves more than minimal risk to subjects, explain any medical treatments that are available if research-related injury occurs, who will provide it, what will be provided, and who will pay for it.*

For any harms stemming from study procedures or assessments, there will be medical and mental health professionals available for consultation and/or referral.

5. Recordings:

- *Will any video or audio recordings be made for research purposes? If so please describe:*

For any harms stemming from study procedures or assessments, there will be medical and mental health professionals available for consultation and/or referral.

All of the Contingency Management sessions will be audio recorded and reviewed to ensure compliance with management strategies. NRT only sessions will also be recorded and a random sample (10-20%) will be reviewed to also ensure fidelity to the protocol and to provide feedback to the clinical pharmacists. No video recording will occur... Dr. David Ledgerwood, the Contingency Management consultant on the project, will be primarily responsible for reviewing the recordings. The audio files will be sent to the Coordinating Center via the Yale OneDrivesystem, which encrypts the recordings. The files will not contain names or other identifying information. The audio files will be stored on Yale Secure Box; only Dr. Edelman, Dr. Bernstein, and Dr. Ledgerwood will have access to the folder. A random sample of the audio files may be transcribed via professional transcription services to allow for qualitative analysis. The recordings will be destroyed after 6 years.

- *How will the data be recorded, transmitted and stored, keeping in mind that this is likely PHI and should be encrypted, etc.?*

Dr. David Ledgerwood, the Contingency Management consultant on the project, will be primarily responsible for reviewing the recordings. The audio files will be sent to the Coordinating Center via the Yale Secure File Transfer system, which encrypts the recordings. The files will not contain names or other identifying information. The audio files will be stored on Yale Secure Box; only Dr. Edelman, Dr. Berstein, and Dr. Ledgerwood will have access to the folder. The recordings will be destroyed after 6 years.

- *How long will the data be held?*

Audio files will be coded by number and will be erased. The recordings will be stored (no identifiers on the recordings) for the duration of the study (6 years) and then destroyed.

- *Is this an optional part of the project, and if not, what is the scientific need to compel people to be recorded in order to participate in the research?*
  This part of the research study is optional

- *How can subjects ask to have the recordings and transcripts destroyed?*
  Subjects may provide a written request for their records and transcripts to be destroyed

6. Provisions to Protect the Privacy Interests of Subjects:

[Note: This section is soliciting *__different information__* *than the confidentiality information solicited in section #5f. Answers will vary widely based on the complexity of the research design, the sensitivity of the subject matter and the populations being recruited.]*

- *Describe the steps that will be taken to protect subjects' privacy interests, particularly a person's desire to control how, where and with whom, they interact and communicate, especially on issues that prospective research participants may deem sensitive or private. Consider privacy interests that may arise from the time participants are identified for recruitment until they complete study participation. Consider privacy interests that may arise in communications with the study subjects (e.g. phone messages, mail, etc), including through long-term follow-up.*

  Participants will first be contacted by research coordinator after their have had the chance to review a letter about the study or a flyer informing them about the study. After receiving the letter, if the participant wishes to opt out, they may do so by calling the research team and give their provided ID. If participant wishes to not participate in the research study, they may end participation at any time.

  The HIPAA Authorization will be reviewed during the consent process with the participants. This Authorization describes how our study is compliant with the HIPAA Authorization and that their information will not shared by anyone outside of the research team.

  Participants are only identified by study ID when providing study data to other sites for analysis. Pharmacists do not include participant's PHI within the recording.

- *Describe what steps you will take to make the subjects feel at ease with the research situation in terms of the questions being asked and the procedures being performed. "At ease" does not refer to physical discomfort, but the sense of intrusiveness a subject might experience in response to questions, examinations, and procedures.*

  Participants will be given continuous opportunities to skip any questions or examination that they are not comfortable completing. Participant will be reminded at time of consent and during study participation. If participant show's signs of being uncomfortable mentally, emotionally, or physically, participant will be asked if they would like to move on to next part of study.

- *Describe why it is acceptable and appropriate for members of the research team to approach the prospective participant about the research.*

  Because cigarette smoking is a major health threat to patients with HIV, we believe that the patient population will benefit from this clinical trial. We only approach those who are interested in participation and hope to improve their smoking behaviors.

7. Economic Impact on Subjects:

- *Describe any foreseeable costs that subjects may incur through participation in the research (exclude billing for procedures that are part of clinical care e.g. copayments for studies that involve an overlap of clinical care & research).*

  If participants choose to come to in-person visits for research they may acquire travel costs. We try to schedule appointments/labs around already scheduled doctor's appointments to minimize this cost.

- *In answering this question, the Financial Administration of Clinical Trials Services (FACTS) must be consulted in determining the appropriate responsible party for subject care costs incurred as part of the clinical research study. Additional information can be found at (FACTS Office)*

  There are no additional care costs involved in this research study.

8. Payments/Reimbursements to Subjects:

Describe the amount and timing of any payments/reimbursements to subjects. Payments must be pro-rated. Completion bonuses may be permitted but may not represent undue inducement. Review ISMMS Finance webpage for restrictions on payments, requirements for tracking cash, gift card, etc. payments.

For baseline, follow up and end of treatment assessments, participants will receive $50 each. Compensation is provided only to patient participants; clinic staff will not be compensated. Based on participant treatment arm and smoking abstinence, participants will be able to participate in raffle system. Participants may be randomized to treatment arm of 0, 5 or 10 total CM visits. If the patient adheres to treatment, they may draw the following amounts in gift cards: $5, $20, $50, $100. The number of draws they receive per session depends on how well they adhere to treatment. Total compensation will vary depending on a participant's adherence to treatment and subsequently the number of raffle draws they receive. For those who receive NRT or VA + CM, the draw schedule will start at 1 and increase by 1 to a cap of 5 with evidence of abstinence. Those who do not respond to NRT+CM and are randomized to CM+, the draw schedule will start at 5 draws and increase by 1. Number of visits vary from 5 to 10.

9. Consent Process:

This section always applies, please indicate whether consent will be obtained from subjects. (If not, proceed to the ***Waiver or Alteration of the Consent Process*** *section below).* *If you will be obtaining consent, describe:*

- *The setting of the consent process.*

For aims 1 and 2, patients will be consenting in person in the clinic where they have been recruited from or remotely. Due to social distancing requirements and safety protocols, if the situation requires, participant may be consented via phone or HIPAA compliant video conferencing with Zoom. Participants can either mail the signed consent or bring back a signed copy in person within two weeks of the phone call/video call or email it to research assistant.

For aim 3, for the survey, clinicians, staff, clinical leadership, social workers, psychologists, and care coordinators will be located wherever they have access to email and the online Redcap survey. They will not be providing any health identifiers and thus there will be no need for HIPAA authorization. They will only need to provide consent prior to study activities. The consent document will be located on the very first page of the survey; participants will not be allowed to proceed unless they agree to the terms of consent.

- *Describe any waiting period available between informing the prospective subject and obtaining the consent.*

Participants will have as much time as needed to thoroughly review the consent form

- *If you will be following "SOP HRP-090 Informed Consent Process for Research", after addressing the points above, indicate this. Otherwise, also describe:*

This study will be following SOP HRP-090 Informed Consent Process for Research

- *The role of the individuals listed in the application as being involved in the consent process.*
- *The time that will be devoted to the consent discussion.*
- *Steps that will be taken to minimize the possibility of coercion or undue influence.*
- *Steps that will be taken to ensure the subjects' understanding.*
- *Describe any tools that will be utilized during the consent process*

Children:

Federal regulations define "children" as persons who have not attained the legal age for consent to treatments or procedures involved in the research [clinical investigation] under the applicable law of the jurisdiction in which the research [clinical investigation] will be conducted (45 CFR 46.402(a) and [21 CFR 50(o)]). If the Human Research involves children:

- *Review the "CHECKLIST HRP-416 Criteria for Research Involving Children" and ensure that your protocol has sufficiently addressed these additional regulatory criteria for approval, paying particular attention to providing protocol specific findings.* N/A
- *Describe the criteria that will be used to determine whether a prospective subject has not attained the legal age for consent to treatments or procedures involved in the Human Research under the applicable law of the jurisdiction in which the Human Research will be conducted (e.g., individuals under the age of 18 years).* N/A
- *NOTE: For research conducted in New York State, review "SOP HRP-013- Legally Authorized Representatives, Children, and Guardians" to be aware of which individuals in the state meet the DHHS and FDA definition of "children" in New York State.* N/A
- *NOTE: For research conducted outside of New York State, obtain consultation from Mount Sinai legal counsel as to the definition of "minor" in the jurisdiction(s) where you are performing your research, given the treatments and procedures involved in the Human Research. [Contact the PPHS Office regarding how to obtain a Legal consultation.] After receiving consultation with Legal, provide an explanation in this section about whether you will be enrolling subjects who are defined as minors in other jurisdictions and the basis for your conclusion that they are not legally capable of consenting to the treatments or procedures involved in the research.* N/A
- *Describe whether parental permission will be obtained from:* N/A

- *Both parents unless one parent is deceased, unknown, incompetent, or not reasonably available, or when only one parent has legal responsibility for the care and custody of the child.*
- *One parent even if the other parent is alive, known, competent, reasonably available, and shares legal responsibility for the care and custody of the child.*

Describe whether permission will be obtained from individuals other than parents, and if so, who will be allowed to provide permission. Describe the process used to determine these individuals' authority to consent to each child's general medical care. N/A

- *Indicate whether assent will be obtained from all, some, or none of the children. If assent will be obtained from some children, indicate which children will be required to assent.* N/A
- *When assent of children is obtained describe whether and how it will be documented.* N/A
- *Describe whether child subjects may be expected to attain legal age to consent to the procedures of the research prior to the completion of their participation in the research (including storage of samples). If so, describe the process that will be used to obtain their legal consent to continue participation in the study. Describe the timing of this process, and what will occur if consent is not obtained from the now-adult subjects. Be certain to include your retention policy here and the consent/assent forms (SEE PPHS POLICY).* N/A

Cognitively Impaired Adults

N/A

- *If the Human Research involves adults who may be unable to consent, describe the process to determine whether an individual is capable of consent.*
- *Indicate who (must be either an attending physician, or independent consulting physician, with appropriate training, licensing and certification qualifications to make the determination regarding capacity) will make the assessment and how the assessment will be made. Pay special attention to the required qualifications regarding assessing incapacity due to mental illness, mental retardation or developmental disability.*

Research involving incapacitated adults must also comply with Mount Sinai Hospital Policies regarding Incapacity (see Policy GPP-312 for further information). Please attest that you will comply with MSH Policy GPP-312 on Consents – revised 6/19 (GPP-312 Consent Policy)

- *The assessment of capacity must include the cause and extent of incapacity and the likelihood that the subject will regain capacity. The plan must also indicate that documentation of the determination and assessment will be placed in the medical record, when applicable, in addition to the research record.*
- *If the Human Research involves cognitively impaired adults:*
- *If permission of a legally authorized representative will be obtained:*
  - *List the individuals from whom permission will be obtained in order of priority.*

For research conducted in New York State, review "SOP HRP-013 Legally Authorized Representatives, Children, and Guardians" to be aware of which individuals in the state meet the DHHS and FDA definition of "legally authorized representative."

  - *For research conducted outside of New York State, obtain consultation from Mount*

Sinai legal counsel as to the definition of "legally authorized representative" in the jurisdiction(s) where you are performing your research. [Contact the PPHS Office regarding how to obtain a Legal consultation.] After receiving consultation with Legal, provide an explanation in this section about which individuals are authorized under applicable law to consent on behalf of a prospective subject to their participation in the procedure(s) involved in this Human Research.

- *Describe the process for assent of the subjects. Indicate whether:*
  - *Assent will be required of all, some, or none of the subjects. If some, indicated, which subjects will be required to assent and which will not.*
  - *If assent will not be obtained from some or all subjects, an explanation of why not.*
  - *Describe whether assent of the subjects will be documented and the process to document assent.*
- *Describe the procedure to consent the individual if they regain capacity during the course of the research study. Keep in mind that this may a considerable time if specimen collection is involved.*

***Non-English Speaking Subjects* (See PPHS policy)**

Indicate what language(s) other than English are understood by prospective subjects or representatives. If subjects who do not speak English will be enrolled, describe the process to ensure that the oral and written information provided to those subjects will be in that language. If you intend to exclude potential participants who do not speak English, provide a justification for doing so.

We intend to recruit Spanish speaking participants once the required forms and consent form are translated and approved.

Waiver or Alteration of the Consent Process

If the Human Research involves a request for a waiver or alteration of the consent process, review the "CHECKLIST HRP-410 Criteria for Waiver or Alteration of the

Consent Process" and make sure your submission provides adequate information for the IRB to assess the criteria for approval. It is highly recommended that you provide additional information here to address each of the criteria for approval (e.g. impracticability).

Examples of when a waiver or alteration of the consent process may be applicable:

- *Research that does not obtain consent from subjects*
- *Research that omits some information that is required in the consent template*
- *Research that involves deception*
- *Research that involves obtaining private information about third parties who have not provided consent*

- *Research that requests a waiver of the consent process for planned emergency research. Please review the "CHECKLIST HRP-506 Criteria for Waiver of the Consent Process for Planned Emergency Research" to ensure you have provided sufficient information for the IRB to make these determinations.*

Please reference the HRP 410 – Waiver or alteration of consent process checklist – uploaded to RUTH.

Requested and had approved an alteration of informed consent for participants in Aim 3.

Aim 3 study procedures, involving clinicians, staff, clinical leadership, social workers, psychologists, and care coordinators , to perform pre and post trial surveys. We will use an information sheet to inform participants of the study. We will not be obtaining signatures. As we will not be collecting private health identifiers as part of Aim 3, there is no need for HIPAA authorization.

Survey data will be collected in a confidential fashion. No health identifiers will be collected. The information we will be focused on collecting will include evidence and context for intervention delivery. Thus we do not anticipate risksfor the clinicians, staff, clinical leadership, social workers, psychologists, and care coordinators participating in the survey .

Because no health identifiers will be collected and due to the nature of the data collected, we do not anticipate that altering informed consent will impact the rights and welfare of the participants. We will use emails to link provider/ staff to a studyID and create a record for them. This will allow us to link the pre/post RC surveys. Data will then be de-identified after linkage is performed to create a de-identified dataset.

The alteration of consent will not adversely affect the rights and welfare of participants- we are not collecting any health identifiers.

It is not practical to conduct this research w/o an alteration as the written informed consent and/or HIPAA authorization would involve documentation of participant identifiers and thus linkage to study participation.

Note that we will be using an information sheet in lieu of written consent- this information sheet implies verbal consent and does not require a signature.

Additional pertinent information will be provided as needed to participants. The investigators are available for contact and questions.

We will be using an information sheet in lieu of written consent- this information sheet implies verbal consent and does not require a signature.

It is not practical to conduct this research w/o an alteration as the written informed consent and/or HIPAA authorization would involve documentation of participant identifiers and thus linkage to study participation. The information sheet provides sufficient information on the study in order for participants to make a determination as to whether they would like to participate. The surveys and participation in the study do not present more than a minimal risk to participants.

10. Process to Document Consent in Writing:

a) *If consent will be obtained at a distance using the written consent form as the official version, please provide details of how the subjects will receive a copy of the consent form, how the consent form will be reviewed with the subject, and how signature will be obtained and documented.*

Due to social distancing requirements and safety protocols, if the situation requires, participant may be consented via phone or HIPAA compliant video conferencing with Zoom. Participants can be either mailed or e-mailed a copy of the consent for their review. Participants can either mail the signed consent or bring back a signed copy in person within two weeks of the phone call/video call or email it to research assistant.

b) *If using any sort of e-Consent on any device, including iOPEN, provide access to the PPHS to allow us to review the built consent. e-Consent is defined as consent designed to be obtained with minimal human interaction, generally using specialized software.*

- *If not iOPEN please provide details and complete the InfoSec "screening questions" for further instructions.*
  N/A

c) *If you want a waiver of written documentation, please review the "CHECKLIST HRP-411" to ensure that your protocol has sufficiently addressed these additional regulatory criteria for approval, including protocol specific information to support the determinations. You will also need to request a waiver of HIPAA authorization. Unless you are eligible for a complete waiver of consent, your consent form or script should still contain the needed elements of consent as laid out in our consent template.*

N/A

11. Vulnerable Populations:

a) *Unless already detailed in the protocol, please indicate which of the following populations are either included or excluded in this project: Indicate specifically whether you will include (target) or exclude each of the following populations:*

Include	*Exclude*	*Vulnerable Population Type*
	X	*Adults unable to consent*
	X	*Individuals who are not yet adults (e.g. infants, children, teenagers)*
	X	*Wards of the State (e.g. foster children)*
	X	*Pregnant women*
	X	*Prisoners*

b) *Describe other aspects of the subject population that may increase their vulnerability (marginalized populations, poverty, illiteracy and under-education, legal status, home/institution-bound individuals; students participating in their professor's research, cognitively-impaired minors, etc.). For those subjects at an increased risk of not understanding the aims, procedures, risks and benefits of this project, OR whom may be at increased vulnerability to coercion or undue influence, describe additional safeguards included to protect their rights and welfare.*

No other aspects foreseen that could increase subjects' vulnerability.
Potential subjects will be informed about the study by screening processes and/or while they are present in clinic for a routine visit. They will be asked how they would prefer to be contacted in the future (in person, by telephone, or by email). Participants will be fully informed of all possible study procedures and will be notified that they may withdraw from the study at any time without any effect on their health care. In addition, participants have the option to skip any questions that may cause them discomfort.
Blood samples will be collected as part of the standard of care in the clinics; these results will be utilized for this study, but no additional study-only phlebotomies are planned. Breath samples to

assess carbon monoxide levels are collected for research purposes and should add no risks other than those normally associated with the collection procedure. The rating scales and structured assessments are all non-invasive and have been utilized in clinical studies with no known negative outcomes and should also add no risks to participants, as our past experience indicates. The main risk associated with the study is the possibility that confidential information obtained during the study will be disclosed. All efforts will be made to protect participants' confidentiality. The alternative to participation is for a potential participant to decide NOT to participate.

c) *What steps are being taken to assure that a diverse group of research subjects are approached to participate in this study? What are the projected demographics of the enrolled subjects at study completion.*

In part of the study design, we recruit at sites with an already diverse patient population. In the future, we plan to expand our patient pool to Spanish speaking participants.

d) *Review the following checklists that are available in RUTH. The research team should provide the "project specific findings" that are requested in the relevant checklists.*
N/A

- *If the Human Research involves cognitively impaired adults, review the "CHECKLIST HRP-417 Criteria for Research Involving Cognitively Impaired Adults" to ensure that your protocol has sufficiently addressed these additional regulatory criteria for approval, including protocol specific information to support the determinations.*

- *If the Human Research involves persons who have not attained the legal age for consent to treatments or procedures involved in the research ("children"), review the "CHECKLIST HRP-416 Criteria for Research Involving Children" to ensure that your protocol has sufficiently addressed these additional regulatory criteria for approval, including protocol specific information to support the determinations.*

- *If the Human Research involves pregnant women, review the "CHECKLIST HRP-412 Criteria for Research Involving Pregnant Women" to ensure that your protocol has sufficiently addressed these additional regulatory criteria for approval, including protocol specific information to support the determinations.*

- *If the Human Research involves non-viable neonates or neonates of uncertain viability, review the "CHECKLIST HRP-413 Criteria for Research Involving Non-Viable Neonates" "CHECKLIST HRP-414 Criteria for Research Involving Neonates of Uncertain Viability" to ensure that your protocol has sufficiently addressed these additional regulatory criteria for approval, including protocol specific information to*

support the determinations.

- *If the Human Research involves Prisoners, review the "CHECKLIST HRP-415 Research Involving Prisoners" to ensure that your protocol has sufficiently addressed these additional regulatory criteria for approval, including protocol specific information to support the determinations.*

12. Multi-Site Human Research:

a) Besides research sites within the Mount Sinai System please detail the PI's responsibilities for other sites, or overall responsibility for the project?

PI Dr. Keith Sigel will be responsible for IRB approval and for monitoring day-to-day study activities at the Mount Sinai site.

b) If coordinating center functions are taking place at Sinai, whether or not it is also a clinical site, please answer the following with appropriate justification and documentation, if needed:

(i) Are the management, data analysis, and Data Safety and Monitoring (DSM) systems adequate, given the nature of the research involved? Yes

(ii) Is the sample protocols and informed consent documents developed and distributed to each collaborating institution? Yes;

(iii) Does each collaborating institution hold an applicable OHRP-approved Assurance?; Yes

(iv)Will each protocol be reviewed and approved by the IRB at the collaborating institution prior to the enrollment of subjects?

Sinai has oversight of the study as the main IRB. The Downstate IRB will conduct an administrative review to verify all local requirements are met, prior to activation, including confirmation of required elements of the informed consent, local required training, and COI disclosures. The Yale IRB verified that local requirements were met when the reliance agreement was put in place Yale had and that local language was included in the consent. After reliance agreement was put in place, Yale tracks local personnel updates and that protocol is renewed each year.

(v) Have all substantive modifications by the collaborating institution to the sample consent, especially related to risks or alternative procedures, been appropriately justified? Yes;

(vi) Will informed consent be obtained from each subject in compliance with HHS regulations? Yes

If this is a multi-site study where you are the lead investigator, describe the management of information (e.g., results, new information, unanticipated problems involving risk to subjects or others, or protocol modifications) among sites to protect subjects.

13. Community-Based Participatory Research

N/A

a) *Describe involvement of the community in the design and conduct of the research.*

(Note: "Community-based Participatory Research" is a collaborative approach to research that involves the community in all aspects of research process. Community-based Participatory Research begins with a research topic of importance to the community and has the aim of combining knowledge with action and achieving social change to improve health outcomes and eliminate health disparities. Simply recruiting participants from the community is not CBPR. If your research does not involve the community in all aspects of the research process, mark N/A)

b) *Composition and involvement of any community advisory board for research conducted outside of MSSM.*

14. Sharing of individual and study Results with Subjects:

a) *If not in the protocol, or in response to other questions (e.g. Radiation Safety) add here. Be sure to remain compliant with NYS laws around genetic testing and the use of research tests, as only FDA or NYS DOH a approved tests from CLIA labs can be used to guide clinical decision making. If results are not being returned, explain the rationale.*

N/A

b) *Specify which results will be returned to subjects and/or their clinical care team. If results are not being returned, explain the rationale.*

Lab results can be viewed on MyChart which is a part of clinical care. If there are an abnormal findings, the principal investigator will contact the participant directly.

c) *How and when will subjects be informed of the study results? The PPHS expects proactive outreach by the study team.*

Lab results can be viewed on MyChart which is a part of clinical care. If there are an abnormal findings, the principal investigator will contact the participant directly.

15. External IRB Review History

If you have previously submitted this protocol for review by an *__external__* *IRB (non-Mount Sinai IRB), provide the name of the reviewing IRB and the associated project identification number. Please include the details of the review with appropriate documentation including the date of review and the IRB contact information.*

N/A

16. Control of Drugs, Biologics, or Devices:

a) *If not included in the protocol, the supplemental questions of the Investigational Drug service or included in other uploaded documents please add here:*

- *If the Human Research involves drugs, biologics, or devices, describe the plans to store, handle, and control those drugs, biologics or devices so that they will be used only on subjects and be used only by authorized investigators. This very well may involve OR personnel, nursing, central sterilization and supplies etc. The plan should be comprehensive and robust.*

  A pharmacist accesses the treatment plan for the participant. Both the clinician and the pharmacist are knowledgeable of the use of these drugs in this specific patient population. Dosing of medications carefully follow a pre-determined dispensing plan based on individual participant's smoking cessation needs. Symptoms and side effects are carefully monitored frequently by the pharmacist. The drugs that are used in this study are FDA approved.

- *Note: If there are required departmental policies that regulate the control of drugs, biologics, or devices, provide that information here.*

- *Note: For studies involving research drugs or biologics, you will need to obtain the approval of Investigational Drug Service (IDS) of the Mount Sinai Pharmacy, regardless of whether you will be utilizing the IDS to manage the control of research drugs and biologics.*

A SMART APPROACH TO TREATING TOBACCO USE DISORDER IN PERSONS LIVING WITH HIV

Protocol Number: STUDY-19-01121

National Clinical Trial (NCT) Identified Number: 1R01CA243910-01

Principal Investigator: Keith M Sigel MD, PhD

<IND/IDE> Sponsor: N/A

Funded by: NCI

Version Number: v.4.0

December 12, 2022

Summary of Changes from Previous Version:

Affected Section(s)	Summary of Revisions Made	Rationale
1.1, 2.1, 2.2, 3, 9.1	Change in primary and secondary outcome measures	At the suggestion of the DSMB
9.1, 9.2, 9.3, 9.4	Change in statistical analysis due to changes in outcome measures and new reference added	At the suggestion of the DSMB and biostatistician
n/a	Extension of timeline to recruit into Year 5	To maximize recruitment, which was slowed during COVID-19 surges. (Original recruitment timeline was through Year 4.)
5.5	Addition of script for public service announcement to be shown in waiting room (SUNY Downstate site only)	To reach patients in the waiting room so that they are aware of the study and can refer themselves for screening
6.3	Sample size description to be deleted	Biostatistician discovered that it is in the wrong section
n/a	Changes made to the MyChart script and recruitment letter (Yale site only)	Requested by the Yale IRB

n/a	Letter to be sent to enrolled study participants who cannot be reached	Some participants can no longer be reached by telephone due to numbers no longer in service
4.1, 7.3	More specific language about texting participants in the study	Requested by Mount Sinai IRB texting will no longer be done on Google Voice as it's not HIPAA compliant and will now be using Zoom Phone

Table of Contents

STATEMENT OF COMPLIANCE 1
1 PROTOCOL SUMMARY 1
1.1 Synopsis 1
1.2 Schema 2
1.3 Schedule of Activities (SoA) 3
2 INTRODUCTION 4
2.1 Study Rationale 4
2.2 Background 5
2.3 Risk/Benefit Assessment 6
2.3.1 Known Potential Risks 6
2.3.2 Known Potential Benefits 6
2.3.3 Assessment of Potential Risks and Benefits 6
3 OBJECTIVES AND ENDPOINTS 7
4 STUDY DESIGN 8
4.1 Overall Design 8
4.2 Scientific Rationale for Study Design 10
4.3 Justification for Dose 10
4.4 End of Study Definition 11
5 STUDY POPULATION 11
5.1 Inclusion Criteria 11
5.2 Exclusion Criteria 11
5.3 Lifestyle Considerations 11
5.4 Screen Failures 11
5.5 Strategies for Recruitment and Retention 12
6 STUDY INTERVENTION 13
6.1 Study Intervention(s) Administration 13
6.1.1 Study Intervention Description 13
6.1.2 Dosing and Administration 13
6.2 Preparation/Handling/Storage/Accountability 13
6.2.1 Acquisition and accountability 13
6.2.2 Formulation, Appearance, Packaging, and Labeling 14
6.2.3 Product Storage and Stability 14
6.2.4 Preparation 14
6.3 Measures to Minimize Bias: Randomization and Blinding 14
6.4 Study Intervention Compliance 14
6.5 Concomitant Therapy 14
6.5.1 Rescue Medicine 14
7 STUDY INTERVENTION DISCONTINUATION AND PARTICIPANT DISCONTINUATION/WITHDRAWAL 14
7.1 Discontinuation of Study Intervention 14
7.2 Participant Discontinuation/Withdrawal from the Study 14
7.3 Lost to Follow-Up 15
8 STUDY ASSESSMENTS AND PROCEDURES 15
8.1 Efficacy Assessments 15
8.2 Safety and Other Assessments 15
8.3 Adverse Events and Serious Adverse Events 15

8.3.1 Definition of Adverse Events (AE) 15
8.3.2 Definition of Serious Adverse Events (SAE) 16
8.3.3 Classification of an Adverse Event 16
8.3.4 Time Period and Frequency for Event Assessment and Follow-Up 17
8.3.5 Adverse Event Reporting 18
8.3.6 Serious Adverse Event Reporting 19
8.3.7 Reporting Events to Participants 19
8.3.8 Events of Special Interest 19
8.3.9 Reporting of Pregnancy 19
8.4 Unanticipated Problems 19
8.4.1 Definition of Unanticipated Problems (UP) 19
8.4.2 Unanticipated Problem Reporting 20
8.4.3 Reporting Unanticipated Problems to Participants 20
9 STATISTICAL CONSIDERATIONS 20
9.1 Statistical Hypotheses 20
9.2 Sample Size Determination 21
9.3 Populations for Analyses 22
9.4 Statistical Analyses 22
9.4.1 General Approach .... **Error! Bookmark not defined.**
9.4.2 Analysis of the Primary Efficacy Endpoint(s) 23
9.4.3 Analysis of the Secondary Endpoint(s) 23
9.4.4 Safety Analyses 24
9.4.5 Baseline Descriptive Statistics 24
9.4.6 Planned Interim Analyses 24
9.4.7 Sub-Group Analyses 24
9.4.8 Tabulation of Individual participant Data 24
9.4.9 Exploratory Analyses 24
10 SUPPORTING DOCUMENTATION AND OPERATIONAL CONSIDERATIONS 25
10.1 Regulatory, Ethical, and Study Oversight Considerations 25
10.1.1 Informed Consent Process 25
10.1.2 Study Discontinuation and Closure 26
10.1.3 Confidentiality and Privacy 26
10.1.4 Future Use of Stored Specimens and Data 27
10.1.5 Key Roles and Study Governance 28
10.1.6 Safety Oversight 29
10.1.7 Clinical Monitoring 29
10.1.8 Quality Assurance and Quality Control 29
10.1.9 Data Handling and Record Keeping 30
10.1.10 Protocol Deviations 31
10.1.11 Publication and Data Sharing Policy 32
10.1.12 Conflict of Interest Policy 32
10.2 Additional Considerations 33
10.3 Abbreviations 34
10.4 Protocol Amendment History 36
11 REFERENCES 38

STATEMENT OF COMPLIANCE

The trial will be carried out in accordance with International Conference on Harmonisation Good Clinical Practice (ICH GCP) and the following:

- United States (US) Code of Federal Regulations (CFR) applicable to clinical studies (45 CFR Part 46, 21 CFR Part 50, 21 CFR Part 56, 21 CFR Part 312, and/or 21 CFR Part 812)

National Institutes of Health (NIH)-funded investigators and clinical trial site staff who are responsible for the conduct, management, or oversight of NIH-funded clinical trials have completed Human Subjects Protection and ICH GCP Training.

The protocol, informed consent form(s), recruitment materials, and all participant materials will be submitted to the Institutional Review Board (IRB) for review and approval. Approval of both the protocol and the consent form must be obtained before any participant is enrolled. Any amendment to the protocol will require review and approval by the IRB before the changes are implemented to the study. In addition, all changes to the consent form will be IRB-approved; a determination will be made regarding whether a new consent needs to be obtained from participants who provided consent, using a previously approved consent form.

1 PROTOCOL SUMMARY

1.1 SYNOPSIS

Title: A SMART Approach to Treating Tobacco Use Disorder in Persons Living with HIV

Study Description: The study aims to answer the main research question, what is the optimal, adaptive strategy to promote long-term (24 week) reduction in average cigarettes smoked per day among patients with HIV?

Objectives:

Primary Objective: The first aim is to identify the optimal adaptive approach to promote a reduction in average cigarettes smoked per day among participants.
Secondary Objectives: The second aim is to identify the impact of various tobacco treatment regimens on confirmed smoking abstinence and HIV-related biomarkers over time, including promoting improvements in: 1) CD4 count; 2) HIV viral load suppression; and 3) VACS Index scores within participants. The third aim is to conduct an implementation focused-evaluation of a pharmacist-delivered intervention involving NRT, VAR or Bupropion, and CM among all the stakeholders (clinicians, staff and leadership).

Endpoints: Primary Endpoint: The primary endpoint will be reduction in average cigarettes smoked per day at 12 and 24 weeks.
Secondary Endpoints: Verified 7-day smoking cessation and change in CD4 count, HIV viral load and VACS index at 12 and 24 weeks for each treatment strategy.

**Study Population:**	18 to 64 Years, 65 Years and Over, Patients, MSSM Employees
**Phase:**	N/A
**Description of Sites/Facilities Enrolling Participants:**	Mount Sinai Hospital, Yale New Haven Hospital, SUNY Downstate
**Description of Study Intervention:**	The purpose of this research study is to compare different strategies, involving medications (nicotine replacement therapy and varenicline or bupropion) and contingency management to help people with HIV reduce daily cigarettes smoked.
**Study Duration:**	10 years
**Participant Duration:**	24 weeks

1.2 SCHEMA

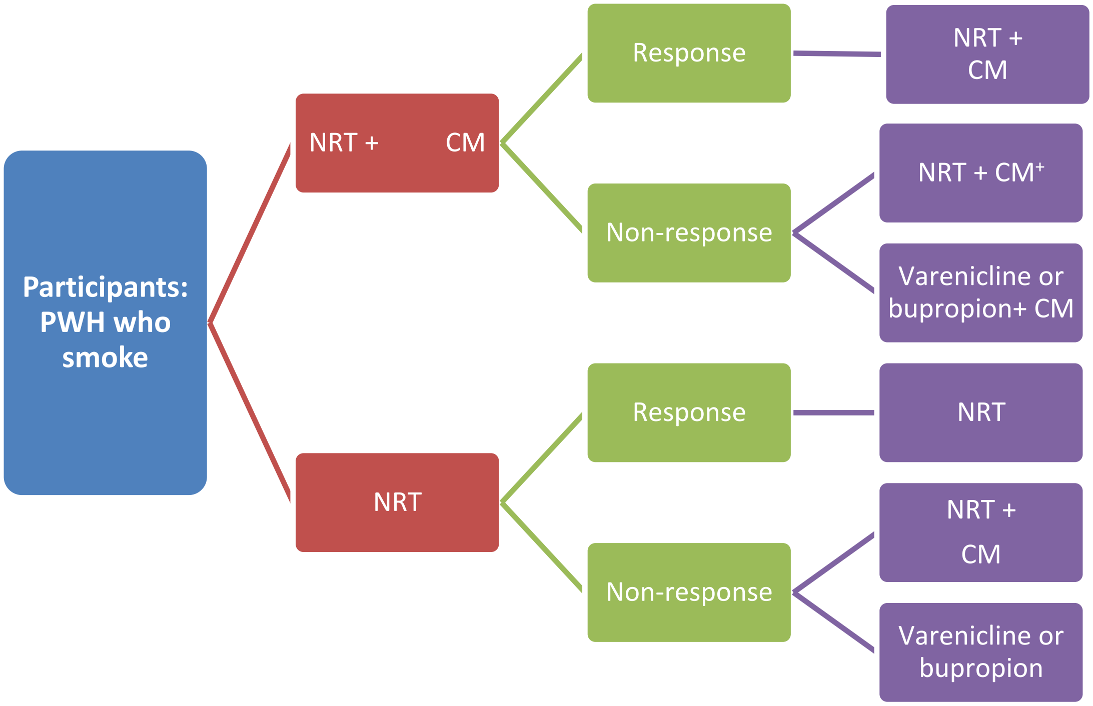

1.3 SCHEDULE OF ACTIVITIES (SOA)

Instrument	Baseline	Week 12	Week 24
Intake History/Patient Demographics	X		
Medical and psychiatric comorbidities	X		
Impulsivity Scale	X		
ASSIST	X		X
PROMIS PROPr	X	X	X
Exhaled carbon monoxide level	X	X	X
Wisconsin Predicting Patients' Relapse Questionnaire	X	X	X
Heaviness of Smoking Index	X	X	X
Self-report of tobacco use	X	X	X
E-cigarette use	X	X	X
HIV biomarkers (CD4 cell count, HIV viral load)	X		X
VACS Index 2.0	X		X
HIV Symptom Index	X	X	X
Treatment Service Review (TSR)	X	X	X
NRT and/or varenicline or bupropion adherence		X	X
CM session attendance, draws and rewards earned	X	X	X
Cost-related data (cost of tobacco, medication co-pay)	X	X	X

Patient Satisfaction Survey		X	X

	0	1	2	3	4	5	6	7	8	9	10	11	12	13	14	15	16	17	18	19	20	21	22	23	24
Ph	X	X		X		X			X				X	X	X			X			X				X
CM		X		X		X			X				X	X	X			X			X				X
As	X												X												X

Study Visits- Ph=pharmacist Visits, CM=Contingency management visits and As=assessments

If participants are unable to attend CM visits due to COVID-19 related restrictions and remote CO monitoring is not available we will accept the attestation, over the phone or in writing, from a family member, partner, or closest informant that they have not smoked in 7 days as a successful CM visit.

In the event of shutdown of in-person human subjects research for studies that are non-COVID- related but have benefit, the study PI will contact any participants reaching key data collection milestones (week 12 or week 24) and ask for permission to do a home visit to perform CO monitoring in an outdoor setting. The study team will perform this home visit if or when it is applicable. Study team will also do remote CM visits with remote CO monitoring as indicated and if possible (i.e., participant has eCO monitor and needed technology).

2 INTRODUCTION

2.1 STUDY RATIONALE

The study aims to answer the main research question, what is the optimal, adaptive strategy to promote long-term (24 week) reduction in average daily cigarettes smoked among patients with HIV? The first aim is to identify the optimal adaptive approach to promote reduction in average cigarettes smoked per day among participants. The second aim is to identify the impact of various tobacco treatment regimens on promoting confirmed smoking abstinence and HIV-related biomarkers over time, including promoting improvements in: 1) CD4 count; 2) HIV viral load suppression; and 3) VACS Index scores within participants. The third aim is to conduct an implementation focused-evaluation of a clinical pharmacist-delivered intervention involving NRT, VAR, bupropion, and CM among all the stakeholders (clinicians, staff and leadership). Aim 3: To inform future implementation efforts of our clinical pharmacist-delivered intervention involving NRT, CM and varenicline and bupropion to promote smoking cessation among patients with HIV, we will conduct a comprehensive evaluation of factors that may impact delivery of this intervention in real-world settings. This evaluation will be based on recruitment experiences; surveys from clinicians, staff, clinical leadership, social workers, psychologists, and care coordinators; and costs associated with the intervention.

2.2 BACKGROUND

Background

Cigarette smoking is a major health threat to patients with HIV (PLWH). Cigarette smoke is uniquely harmful among PWH due to effects on HIV and the immune system. The Veterans Aging Cohort Study (VACS) Index, a validated measure that predicts morbidity and mortality risk, correlates with markers of inflammation, is responsive to smoking status, and may serve as an outcome in intervention studies. Smoking cessation is consistently recommended for PWH, yet current approaches are inadequate. There is a potential role for clinical pharmacists in delivering smoking cessation treatment in HIV treatment settings. Nicotine replacement therapy (NRT), varenicline (VAR) and bupropion are effective treatments and safe in HIV. Contingency management (CM) promotes smoking cessation and has been used with PWH. Adaptive treatment regimens (ATRs) that are responsive to patients' needs and outcomes may be more effective than static pathways for addressing tobacco use. An important gap in our understanding of tobacco treatment among PWH is the lack of an evidence-based ATR. The overarching goal of this study is to identify such a regimen with the Sequential Multiple Assignment Randomized Trial (SMART) design, which allows adjustments in treatment according to patient response, and to develop optimal regimens.

Primary and Secondary Study Endpoints

Aim 1: The primary endpoint will be mean number of cigarettes smoked per day. Tobacco use will be assessed by self-report.).

To complete Aim 1, we will evaluate the impact of the initial treatment strategy on 12 week mean number of cigarettes smoked per day and, among participants who do not respond at week 12, evaluate the impact of switching from NRT to an oral medication (varenicline or bupropion) compared to intensifying contingency management on 24 week mean number of cigarettes smoked per day. We will then identify the optimal treatment strategy.

Aim 2. For secondary outcomes, we will assess smoking abstinence by both self-report and confirmatory biochemical testing with exhaled carbon monoxide (eCO) or verification of abstinence by closest informant (if unable to obtain eCO.) To assess the impact of the smoking cessation treatment strategies on HIV outcomes, we will examine change in CD4 count, HIV viral load and VACS index at 12 and 24 weeks for each treatment strategy.

Aim 3: We will conduct a survey upon initiation of the RCT and post-RCT to assess knowledge, attitudes and practice of a clinical pharmacist-delivered smoking cessation intervention involving medications and contingency management, including the 12-item Organization Readiness for Implementation Change (ORIC) assessment and Contingency Management Beliefs Questionnaire (CMBQ). The ORIC has been widely applied to the evaluation of interventions intended to promote evidence-based practices. The CMBQ has been validated to assess factors impacting implementation of CM. Differences in pre-post trial responses in the survey items will be assessed using appropriate parametric or non-parametric methods. We will assess cost variation based on potential other providers of the intervention (e.g., physician, nurse, advanced practice practitioner, social worker). We will assess participant satisfaction with the intervention at week 24.

2.3 RISK/BENEFIT ASSESSMENT

2.3.1 KNOWN POTENTIAL RISKS

Potential participants will be informed about the study by screening processes and/or while they are present in clinic for a routine visit. They will be asked how they would prefer to be contacted in the future (in person, by telephone, by text, or by email). Participants will be fully informed of all possible study procedures and will be notified that they may withdraw from the study at any time without any effect on their health care. In addition, participants have the option to skip any questions that may cause them discomfort.

Blood samples will be collected as part of the standard of care in the clinics; these results will be utilized for this study, but no additional study-only phlebotomies are planned. Breath samples to assess carbon monoxide levels are collected for research purposes and should add no risks other than those normally associated with the collection procedure. The rating scales and structured assessments are all non-invasive and have been utilized in clinical studies with no known negative outcomes and should also add no risks to participants, as our past experience indicates. The main risk associated with the study is the possibility that confidential information obtained during the study will be disclosed. All efforts will be made to protect participants' confidentiality. The alternative to participation is for a potential participant to decide NOT to participate.

2.3.2 KNOWN POTENTIAL BENEFITS

Possible benefits may be that the participant is able to stop smoking, which may greatly improve their overall health. In addition, this study will generate new information about the role for clinical pharmacists in optimizing strategies to help people with HIV stop smoking.

2.3.3 ASSESSMENT OF POTENTIAL RISKS AND BENEFITS

The main risks to the participant are possible side effects to nicotine replacement therapy, varenicline or bupropion.

- NRT: There is a very small risk of nicotine toxicity, if nicotine replacement products are used in higher quantities than prescribed or from smoking while using the products. Symptoms of nicotine toxicity include cold sweats, fainting, confusion, or pounding heart. The Food and Drug Administration (FDA) has published new guidelines, which suggest that these products are safe to use longer than 12 weeks. Based on the study design, the risks of toxicity are minimized by having the clinical pharmacist prescribe the dosage of nicotine replacement therapy based on how much the participant smokes daily.
- Varenicline: The main risks of varenicline are nausea at onset of use that is generally mitigated by taking before meals and usually abates over time. Sleep disturbance, including vivid dreams, has been reported. Less common effects include indigestion, abdominal pain, and gas, fatigue, headache and dry mouth.
- Bupropion: The main risks of bupropion include increased risk of seizure; thus, it should be avoided in patient with an underlying seizure disorder, eating disorder, untreated alcohol use disorder, and/or concurrent use of a monoamine oxidase inhibitor (MAOI). Common side effects may include insomnia, dry mouth, and constipation. Uncommon side effects may include rash, itching or hives. The medicine may also cause dizziness; thus, individuals should avoid driving or

using heavy machinery before they know how they might react. The medicine may also cause some patients to have abnormal behaviors or worsening mood or suicidal thoughts. Participants will be instructed to report any of these symptoms immediately to the research team.

3 OBJECTIVES AND ENDPOINTS

OBJECTIVES	ENDPOINTS	JUSTIFICATION FOR ENDPOINTS
Primary		
Aim 1: The primary endpoint will be mean of cigarettes smoked per day. .	Tobacco use will be assessed by self-report<br><br>To complete Aim 1, we will evaluate the impact of the initial treatment strategy on 12-week mean number of cigarettes per day and, among participants who do not respond at week 12, evaluate the impact of switching from NRT to varenicline or bupropion compared to intensifying contingency management on 24-week mean number of cigarettes per day.	This endpoint was chosen to identify the optimal treatment strategy on promoting harm reduction from tobacco use.
Secondary		
Aim 2. For secondary outcomes and to assess the impact of the smoking cessation treatment strategies tobacco abstinence and on HIV outcomes.	We will evaluate abstinence by confirmatory biochemical testing with eCO or verification by close informant.<br><br>We will examine change in CD4 count, HIV viral load and VACS index at 12 and 24 weeks for each treatment strategy.	This endpoint was chosen to see if and how the treatment strategies promote abstinence and whether smoking cessation treatment affects HIV outcomes.
Tertiary/Exploratory		
Aim 3: We will conduct a survey upon initiation of the RCT and post-RCT.	We will conduct a survey upon initiation of the RCT and post-RCT, including the 12-item Organization Readiness for Implementation Change (ORIC) assessment and Contingency Management Beliefs Questionnaire (CMBQ). The ORIC has been widely applied to the evaluation	To assess knowledge, attitudes and practice of a clinical pharmacist-delivered smoking cessation intervention involving medications and contingency management and

	of interventions intended to promote evidence-based practices. and the CMBQ has been validated to assess factors impacting implementation of CM. Differences in pre-post trial responses in the survey items will be assessed using appropriate parametric or non-parametric methods.	to assess cost variation based on potential other providers of the intervention (e.g., physician, nurse, advance practice practitioner, social worker). We will assess participant satisfaction with the intervention at week 24.

4 STUDY DESIGN

4.1 OVERALL DESIGN

Description of the Study Design

To address aims 1 and 2, we plan to use a two-arm, two-stage randomized trial design of 632 patients with HIV who smoke cigarettes and receive their HIV care in one of three health systems.
To address aim 3, we plan to hold pre- and post-trial surveys involving clinicians, staff, clinical leadership, social workers, psychologists, and care coordinators to conduct an implementation focused-evaluation of a clinical pharmacist-delivered intervention.

Description of Procedures Being Performed

For aims 1 and 2, at baseline, intake history, demographics, questionnaires relating to depression, drug and alcohol problems, smoking habits, medication adherence, HIV symptoms and quality of life will be performed. The depression, substance use, quality of life questionnaires, HIV symptom index and costs related to tobacco use and clinic travel will be administered at weeks 12 and 24. Tobacco measures will be performed at all time points - relating to carbon monoxide levels, cigarette consumption, nicotine dependence, tobacco use and e-cigarette use. HIV related measures pertaining to CD4 count, viral load and other lab values, health care utilization (ED visits, hospitalizations), and treatment services to address tobacco use will be collected at baseline, week 24 and month 12. Patient satisfaction, opinions about services and adherence will also be recorded. At baseline, participants will be randomized 1:1 to either NRT (nicotine replacement therapy) or NRT + CM. Participants will receive a prescription for 12 weeks of the long-acting NRT patches and short-acting NRT (i.e., gum, lozenges, an inhaler or spray) with standard advice. Participants will fill the prescription through their usual pharmacy of choice.
Participants will be invited to complete five follow-up visits with the clinical pharmacist over the following 12 weeks to address medication adherence, side effects and review progress in achieving smoking abstinence. For those randomized to additionally receive contingency management (CM), these visits will be integrated into the sessions with the clinical pharmacist. At week 12, participants who responded to initial treatment will continue without change. Participants who do not respond to the intervention, will be re-randomized, to switch to either VAR or bupropion or intensify to CM+.
Participants will be invited to return to visit the clinical pharmacist for an additional 5 visits, with the final visit at week 24. All of the CM sessions will be audio recorded and reviewed to ensure compliance

with management strategies. NRT only sessions will also be recorded and a random sample (10-20%) will be reviewed to also ensure fidelity to the protocol and to provide feedback to the clinical pharmacists. No video recording will occur. Dr. David Ledgerwood, the CM consultant on the project, will be primarily responsible for reviewing the recordings. The audio files will be sent to the Coordinating Center via the Yale OneDrivesystem, which encrypts the recordings. The files will not contain names or other identifying information. The audio files will be stored on Yale Secure Box; only Dr. Edelman, Dr. Bernstein, and Dr. Ledgerwood will have access to the folder. A random sample of the audio files may be transcribed via professional transcription services to allow for qualitative analysis. The recordings will be destroyed after 6 years.

For aim 3, all clinicians, staff, clinical leadership, social workers, psychologists, and care coordinators employed at the participating site will be invited to complete the brief surveys pre- and post- trial intervention, administered through REDCap and distributed via email. The survey will assess knowledge, attitudes and practices that may impact implementation of a clinical pharmacist delivered intervention involving medications and CM and will be modeled after the ORIC and CMBQ and other surveys.

To maintain safety procedures, in some circumstances, patients can participate in study activities/counseling, and undergo surveys and be consented for the study, via video conferencing link or phone. Patients will be given a link to video conference with provider/clinical pharmacist/coordinator to maintain social distancing guidelines. Zoom version provided is HIPAA compliant per Sinai. Or if using phone for study procedures, patients will be contacted via the phone number they provided.

Description of the Source Records that Will Be Used to Collect Data About Subjects

Participants will be verbally asked during pre-screening (in person and/or phone screening) whether we can collect and store some demographic data and the answers to their pre-screening questions. Information from the phone and in person screeners will (1) only be used to determine eligibility, (2) will not be included in study analyses other than to determine (a) the proportion of eligibility among persons approached (b) the characteristics of those who decline to participate among those eligible. This will be done using HIPAA compliant Zoom Phone. Pre-screening questions include current and lifetime smoking habits, language spoken, study eligibility, demographic questions include age, gender, race, ethnicity. This information will be captured in REDCap with no identifiers. For Aims 1 and 2, electronic medical records, lab results, questionnaire answers, responses on measures, will all be used to provide data about the participants.

Primary Care provider records:
- HIV/AIDS status, hepatitis infection status, sexually transmitted diseases, physical exam (height, weight, blood pressure), diagnosis and treatment of medical and psychiatric conditions, sexual practices, tobacco and alcohol use, use of drugs, smoking status and habits, medication list, pregnancy status, language abilities, adverse events, side effects, transportation

Specialist provider records:
- HIV/AIDs status, hepatitis infection status, sexually transmitted diseases, physical exam (height, weight, blood pressure), diagnosis and treatment of medical and psychiatric conditions, sexual practices, tobacco and alcohol use, use of illegal drugs, smoking status and habits, medication list, adverse events, side effects, transportation, time/costs travel to clinic

Laboratory records, other test results, and treatment services:

CD4 count, hepatitis infection status, sexually transmitted disease, viral load, creatinine, hemoglobin, WBC, eGFR, albumin, platelets, AST, ALT, Body-mass index, tobacco treatment services, hospitalizations, and emergency department visits.

Psychiatrist/therapy records:
-as needed to help determine presence of an unstable psychiatric condition and whether patient meets exclusion criteria, phone call records: diagnosis and treatment of medical and psychiatric conditions, adverse events, side effects

Demographics from electronic medical record:
-First name, last name, gender, date of birth, MRN, telephone number, employment status, insurance, income, self-identified gender identity, race, ethnicity, education, mailing address, proxies/alternate contacts

For Aim 3, survey data will be collected in a confidential fashion and administered through REDCap. The ORIC has been applied to the evaluation of interventions intended to promote evidence-based practices, and the CMBQ is a validated measure to assess providers' perspectives regarding CM. Information will be collected to allow for linkage pre and post survey data and measure change in responses.

Description of Data that Will Be Collected Including Long-Term Follow-Up
There will not be any long term follow up for Aims 1 & 2 except for the 12 month chart review for lab data and treatment services.

For Aim 3, in year 5, the post-trial survey will be sent out. The same information will be collected during the post-trial survey.

4.2 SCIENTIFIC RATIONALE FOR STUDY DESIGN

There are no potential problems associated with the control group chosen in light of the SMARTTT intervention.

4.3 JUSTIFICATION FOR DOSE

Prescribe dosing of patch based on patient’s level of tobacco use
5-9 cigarettes per day: 14mg patch
>10 cigarettes per day: 21mg patch

Prescribe short-acting NRT based on timing of first daily cigarette:
First cigarette is within 30 minutes of waking: 4mg
First cigarette is after 30 minutes of waking: 2mg

Varenicline and bupropion dosing is prescribed per standard clinical guidelines and at the discretion of the clinical pharmacist.

Varenicline: 0.5 mg each day for 3 days, then -0.5 mg twice per day for 4 days, then 1mg twice per day.

Bupropion: 150 mg for 3 days, then 150mg twice per day.

4.4 END OF STUDY DEFINITION

A participant is considered to have completed the study if he or she has completed all phases of the study including the last visit or the last scheduled procedure shown in the Schedule of Activities (SoA), Section 1.3.

5 STUDY POPULATION

5.1 INCLUSION CRITERIA

Inclusion Criteria

For Aims 1 and 2: Patients will be eligible for study entry if they: 1. are HIV positive; 2. Receive care at 1 of the participating sites; 3. are >=18 years old; 4. have smoked >=100 cigarettes lifetime; 5. report smoking every day or some days; 6. smoke, on average, >=5 cigarettes per day; and 7. provide written informed consent. A HIPAA waiver will be requested to allow prescreening via patient chart review.

For Aim 3: All clinicians, staff, clinical leadership, social workers, psychologists, and care coordinators employed at the participating site will be invited to complete the brief surveys pre- and post- trial intervention. Survey data will be collected in a confidential fashion and administered through REDCap. Surveys will be distributed by email and clinicians and staff will be informed and encouraged to participate by clinical leadership. An alteration of written informed consent will be requested for Aim 3.

5.2 EXCLUSION CRITERIA

Exclusion Criteria

For Aims 1 and 2: Patients will be excluded for: 1. exclusive use of non-cigarette tobacco or nicotine products, including e-cigarettes; 2. current use of NRT, VAR, or bupropion (defined as use of medication in prior 7 days); 3. self-report or urine testing confirming pregnancy, nursing, or trying to conceive; 4. Life-threatening or unstable medical, surgical, or psychiatric condition; 5. inability to provide at least 1 collateral contact for a friend or family member; 6. living out-of-state; or 7. inability to read or understand English or Spanish.

For Aim 3: Participants will be excluded for 1) less than 18 years of age and 2) NOT employed at the HIV clinic.

Enrollment Restrictions Based Upon Gender, Pregnancy, Childbearing Potential, or Race
Yes

5.3 LIFESTYLE CONSIDERATIONS

If the participant is a woman and wants to take part of the study, they need to use birth control while they are participating in the study.

5.4 SCREEN FAILURES

People who are pre-screened are assigned a study ID will be tracked so we know who is eligible and ineligible.

5.5 STRATEGIES FOR RECRUITMENT AND RETENTION

How Participants Will Be Identified

For Aims 1 and 2, potential participants will be identified by chart review or via data reports filtered on eligibility criteria, in advance of the clinic. We will develop recruitment flyers specific to each site . Potential participants may be referred to the study team by a clinic provider or staff member. In addition, eligible patients may be informed about the study by messaging via medical record as applicable at a given site (e.g., Epic MyChart). Patients may also self-refer after hearing about the study from a public service announcement in the clinic waiting room.

For Aim 3, all clinicians, staff, clinical leadership, social workers, psychologists, and care coordinators employed at the participating site will be invited to complete the brief surveys pre- and post- trial intervention.

Who Will Initially Approach Potential Participants

Study Personnel

How Research Will Be Introduced to Participants

Potentially eligible patients may be recruited via multiple pathways:

1. Direct referral by clinic provider or staff;
2. Self-referral based on recruitment flyer, peer, or otherwise;
3. Proactive, opt-out recruitment based on electronic medical record review, followed by mailed letter and, if the patient does not opt out of being called, research team outreach; and/or
4. Messaging through the secure medical record with invitation to participate as available at a given site and per site policies.
5. Self-referral via a public service announcement on video monitors in the clinic waiting room.

Individuals who meet inclusion and exclusion criteria and provide written consent to participate will have baseline assessments performed, and then will be randomized 1:1 to either NRT or NRT+CM.

For the Yale New Haven Hospital site only (Aims 1 and 2):

To assist in recruitment, the Yale Clinical Center for Investigation (YCCI) will help to identify potential eligible participants. The YCCI team will mail recruitment letters and post social media advertisements. In addition, they will send announcements through the Help Us Discover database, which contains several thousand individuals who volunteer for clinical research. Additionally, they will send messages through the secure messaging system through the medical record (MyChart via Epic) for those patients who have opted in to being contacted for clinical research studies.

For the SUNY/Downstate site only (Aims 1 and 2):

A public service announcement (PSA) will play on a loop on video monitors in clinic waiting areas. The PSA will give a brief description of the study and will include a contact telephone number for patients to contact the study team.

For Aim 3, all clinicians, staff, clinical leadership, social workers, psychologists, and care coordinators employed at the participating site will be invited to complete the brief surveys pre- and post- trial intervention. The pre-trial survey will occur within the first 3 months of the RCT launch; the post-trial survey will occur within the last three months of the RCT completion.

6 STUDY INTERVENTION

6.1 STUDY INTERVENTION(S) ADMINISTRATION

6.1.1 STUDY INTERVENTION DESCRIPTION

Refer to these documents that were submitted and approved by the IRB:
Nicorette insert
Nicorette lozenge insert
Nicorette patch insert
Varenicline (Chantix) insert
Bupropion

6.1.2 DOSING AND ADMINISTRATION

6.2 PREPARATION/HANDLING/STORAGE/ACCOUNTABILITY

6.2.1 ACQUISITION AND ACCOUNTABILITY

The clinical pharmacist during the study visit will control the product (based on the images of the drugs that we submitted and were approve by the IRB) based on how much the participant smokes:

Prescribe dosing of patch based on patient’s level of tobacco use
5-9 cigarettes per day: 14mg patch
>10 cigarettes per day: 21mg patch

Prescribe short-acting NRT based on timing of first daily cigarette:
First cigarette is within 30 minutes of waking: 4mg
First cigarette is after 30 minutes of waking: 2mg

Prescribe varenicline as follows:
Days 1-3: 0.5 mg once per day
Days 4-7: 0.5 mg twice per day
Day 8 until end of treatment: 1 mg twice per day

Prescribe bupropion as follows:
Days 1-3: 150mg per day
Day 4 until end of treatment: 150mg twice per day

6.2.2 FORMULATION, APPEARANCE, PACKAGING, AND LABELING

Refer to sections 6.1.1 and 6.2.1

6.2.3 PRODUCT STORAGE AND STABILITY

N/A

6.2.4 PREPARATION

N/A

6.3 MEASURES TO MINIMIZE BIAS: RANDOMIZATION AND BLINDING

Randomization is conducted through REDCap, clinical trials data management system. Participants, clinical pharmacists, nor research coordinators are blinded to treatment condition.

6.4 STUDY INTERVENTION COMPLIANCE

Study intervention compliance will be recorded in REDCap based on self-reported information of medication and compliance will be measured based on participants showing up to pharmacy visits which will also be recorded on REDCap.

6.5 CONCOMITANT THERAPY

6.5.1 RESCUE MEDICINE

N/A

7 STUDY INTERVENTION DISCONTINUATION AND PARTICIPANT DISCONTINUATION/WITHDRAWAL

7.1 DISCONTINUATION OF STUDY INTERVENTION

The intervention may be discontinued if a participant has adverse effects from the prescribed medications. However, the participant will remain in the study for follow-up assessments.

7.2 PARTICIPANT DISCONTINUATION/WITHDRAWAL FROM THE STUDY

Anticipated Circumstances of Subject Withdrawal

Participants may be withdrawn from the research without their consent if they are deemed ineligible for the study. Participants may also be withdrawn from the research if they have experienced serious or related adverse events. If any participant asks to withdraw from the study, they will be notified that medical record data collection will continue, but any interventional procedures may cease. If a participant asks for complete withdrawal from the study, he or she must do so by written notification to the site-PI.

Procedures for Subjects to Request Withdrawal

If any participant asks to withdraw from the study, they will be notified that medical record data collection will continue, but any interventional procedures may cease. If a participant asks for complete withdrawal from the study, they must do so by written notification to the site PI.

Procedures for Investigator to Withdraw Subjects
Participants may be withdrawn from the research without their consent if they are deemed ineligible for the study. Participants may also be withdrawn from the research if they have experienced serious or related adverse events. If participants are lost to follow-up, they will not be withdrawn. We will try to continue to get assessments through 24 weeks and complete data collection via the electronic medical record, unless consent is withdrawn in writing.

7.3 LOST TO FOLLOW-UP

Site staff will make every effort to contact study participants, including calling and texting alternate telephone numbers using the Zoom Phone application. In addition, site staff will try to meet participants when they are in clinic for their regular visits with their providers. If they are unable to reach a participant, a letter will be sent to their last known mailing address. Participants should not be considered lost to follow up until 6 months after the 24 weeks have elapsed.

8 STUDY ASSESSMENTS AND PROCEDURES

8.1 EFFICACY ASSESSMENTS

We will have participants do labs to study the longitudinal impact of smoking on the Veterans Aging Cohort Study (VACS) Index, a validated measure that predicts morbidity and mortality risk. Based on routinely collected lab tests (CD4 count, HIV viral load, hemoglobin, AST, ALT, platelets, creatinine, and HCV status, albumin, white blood cell count), the VACS Index correlates with measures of inflammation, microbial translocation, and impaired T cell activation known to be elevated with HIV and smoking. Exhaled carbon monoxide testing will also be collected to confirm abstinence.

8.2 SAFETY AND OTHER ASSESSMENTS

In this study we will collect lab work from participants that includes their physical examinations, CD4 count, viral load, diagnostic labs (metabolic panel) and eCO.

8.3 ADVERSE EVENTS AND SERIOUS ADVERSE EVENTS

8.3.1 DEFINITION OF ADVERSE EVENTS (AE)

Grading System to Evaluate Adverse Events

The following scale will be used in grading the severity of adverse events noted during the study:
1. Mild adverse event
2. Moderate adverse event.
3. Severe unanticipated adverse event resulting inpatient hospitalization or prolongation of existing hospitalization, a persistent or significant disability/incapacity, or a congenital anomaly/birth defect.

4. Life-threatening adverse event
5. Fatal adverse event

8.3.2 DEFINITION OF SERIOUS ADVERSE EVENTS (SAE)

An adverse event (AE) or suspected adverse reaction is considered "serious" if, in the view of either the investigator or sponsor, it results in any of the following outcomes: death, a life-threatening adverse event, inpatient hospitalization or prolongation of existing hospitalization, a persistent or significant incapacity or substantial disruption of the ability to conduct normal life functions, or a congenital anomaly/birth defect. Important medical events that may not result in death, be life-threatening, or require hospitalization may be considered serious when, based upon appropriate medical judgment, they may jeopardize the participant and may require medical or surgical intervention to prevent one of the outcomes listed in this definition. Examples of such medical events include allergic bronchospasm requiring intensive treatment in an emergency room or at home, blood dyscrasias or convulsions that do not result in inpatient hospitalization, or the development of drug dependency or drug abuse.

8.3.3 CLASSIFICATION OF AN ADVERSE EVENT

8.3.3.1 SEVERITY OF EVENT

- **Mild** – Events require minimal or no treatment and do not interfere with the participant’s daily activities.
- **Moderate** – Events result in a low level of inconvenience or concern with the therapeutic measures. Moderate events may cause some interference with functioning.
- **Severe** – Events interrupt a participant’s usual daily activity and may require systemic drug therapy or other treatment. Severe events are usually potentially life-threatening or incapacitating. Of note, the term “severe” does not necessarily equate to “serious”.]

Grading System to Evaluate Adverse Events
The following scale will be used in grading the severity of adverse events noted during the study:
1. Mild adverse event: Events require minimal or no treatment and do not interfere with the participant’s daily activities.
2. Moderate adverse event: Events result in a low level of inconvenience or concern with the therapeutic measures. Moderate events may cause some interference with functioning.
3. Severe unanticipated adverse event: resulting inpatient hospitalization or prolongation of existing hospitalization, a persistent or significant disability/incapacity, or a congenital anomaly/birth defect.
4. Life-threatening adverse event
5. Fatal adverse event

8.3.3.2 RELATIONSHIP TO STUDY INTERVENTION

The degree of certainty about causality will be graded using the categories below. In a clinical trial, the study product must always be suspect.

- **Related** – The AE is known to occur with the study intervention, there is a reasonable possibility that the study intervention caused the AE, or there is a temporal relationship between the study intervention and event. Reasonable possibility means that there is evidence to suggest a causal relationship between the study intervention and the AE.
- **Not Related** – There is not a reasonable possibility that the administration of the study intervention caused the event, there is no temporal relationship between the study intervention and event onset, or an alternate etiology has been established.

OR

- **Definitely Related** – There is clear evidence to suggest a causal relationship, and other possible contributing factors can be ruled out. The clinical event, including an abnormal laboratory test result, occurs in a plausible time relationship to study intervention administration and cannot be explained by concurrent disease or other drugs or chemicals. The response to withdrawal of the study intervention (dechallenge) should be clinically plausible. The event must be pharmacologically or phenomenologically definitive, with use of a satisfactory rechallenge procedure if necessary.
- **Probably Related** – There is evidence to suggest a causal relationship, and the influence of other factors is unlikely. The clinical event, including an abnormal laboratory test result, occurs within a reasonable time after administration of the study intervention, is unlikely to be attributed to concurrent disease or other drugs or chemicals, and follows a clinically reasonable response on withdrawal (dechallenge). Rechallenge information is not required to fulfill this definition.
- **Potentially Related** – There is some evidence to suggest a causal relationship (e.g., the event occurred within a reasonable time after administration of the trial medication). However, other factors may have contributed to the event (e.g., the participant's clinical condition, other concomitant events). Although an AE may rate only as "possibly related" soon after discovery, it can be flagged as requiring more information and later be upgraded to "probably related" or "definitely related", as appropriate.
- **Unlikely to be related** – A clinical event, including an abnormal laboratory test result, whose temporal relationship to study intervention administration makes a causal relationship improbable (e.g., the event did not occur within a reasonable time after administration of the study intervention) and in which other drugs or chemicals or underlying disease provides plausible explanations (e.g., the participant's clinical condition, other concomitant treatments).
- **Not Related** – The AE is completely independent of study intervention administration, and/or evidence exists that the event is definitely related to another etiology. There must be an alternative, definitive etiology documented by the clinician.]

8.3.3.3 EXPECTEDNESS

Site PI will be responsible for determining whether an adverse event (AE) is expected or unexpected. An AE will be considered unexpected if the nature, severity, or frequency of the event is not consistent with the risk information previously described for the study intervention.

8.3.4 TIME PERIOD AND FREQUENCY FOR EVENT ASSESSMENT AND FOLLOW-UP

The occurrence of an adverse event (AE) or serious adverse event (SAE) may come to the attention of study personnel during study visits and interviews of a study participant presenting for medical care, or upon review by a study monitor.

All AEs including local and systemic reactions not meeting the criteria for SAEs will be captured on the appropriate case report form (CRF). Information to be collected includes event description, time of onset, clinician’s assessment of severity, relationship to study product (assessed only by those with the training and authority to make a diagnosis), and time of resolution/stabilization of the event. All AEs occurring while on study must be documented appropriately regardless of relationship. All AEs will be followed to adequate resolution.

Any medical condition that is present at the time that the participant is screened will be considered as baseline and not reported as an AE. However, if the study participant’s condition deteriorates at any time during the study, it will be recorded as an AE.

Changes in the severity of an AE will be documented to allow an assessment of the duration of the event at each level of severity to be performed. AEs characterized as intermittent require documentation of onset and duration of each episode.

The site-PIs will record all reportable events with start dates occurring any time after informed consent is obtained until 7 (for non-serious AEs) or 30 days (for SAEs) after the last day of study participation. At each study visit, the investigator will inquire about the occurrence of AE/SAEs since the last visit. Events will be followed for outcome information until resolution or stabilization.

8.3.5 ADVERSE EVENT REPORTING

The principal investigators (E. Jennifer Edelman, MD, MHS and Steven L. Bernstein, MD) will conduct a review of all adverse events upon completion of every study participant. The MPIs will evaluate the frequency and severity of the adverse events and determine if modifications to the protocol or consent form are required. Dr. Keith Sigel will be responsible for IRB approval and for monitoring day-to-day study activities at the Mount Sinai site. Dr. Jessica Yager will be responsible for study activities at STAR.

Selection Procedures to Minimize Toxicity

Symptoms associate with nicotine "overdose" include cold sweats, fainting, confusion, and pounding heart. Toxicity resolves in several hours. Cases of nicotine toxicity are extremely rare. In terms of the patch, nicotine continues to enter the bloodstream for several hours after removing it, as it leaches through the skin, so smoking within 12 hours of removing the patch is strongly discouraged. One of the most common side effects of the patch is a localized skin rash due to a reaction to the adhesive on the patch or a reaction to the nicotine. The second most common side effect is sleep disturbance. This side effect is common in people who use the 24-hour patch. Nicotine can lead to vivid, colorful dreams and difficulty sleeping. For this study, the 24-hour patch will be utilized. Switching to a 16-hour patch might alleviate this problem, but it often can result in nicotine cravings. Patients using the nicotine inhaler may experience cough. There is also a very small risk that the participant could have an allergic reaction to the drug in any of these products. If this occurs, the participant will be asked to notify the study team at once and seek immediate medical attention. Symptoms of an allergic reaction include rash, itching, swelling, dizziness, and trouble breathing.

VAR may cause nausea in some users, generally mitigated by taking before meals, and usually abating over time. Sleep disturbance, including vivid dreams, have been reported. Less common side effects include indigestion, abdominal pain and gas, fatigue, headache, and dry mouth. If these occur, the research participant will be asked to notify study personnel.

The main risks of bupropion include increased risk of seizure thus it should be used with caution in patient with an underlying seizure disorder, eating disorder, untreated alcohol use disorder, and/or concurrent use of a monoamine oxidase inhibitor (MAOI). Common side effects may include insomnia, dry mouth, and constipation. Uncommon side effects may include rash, itching or hives. The medicine may also cause dizziness thus individuals should avoid driving or using heavy machinery before they know how they might react. The medicine may also cause some patients to have abnormal behaviors or worsening mood or suicidal thoughts. Participants will be instructed to report any of these symptoms immediately to the research team.

8.3.6 SERIOUS ADVERSE EVENT REPORTING

Serious adverse events, whether related or unrelated, will be reported to the MPIs and the IRB as soon as study staff become aware of the event.

Reporting Events to Participants

Study participants will be notified individually of any new findings or adverse events which may affect their participation in the trial.

8.3.7 EVENTS OF SPECIAL INTEREST

N/A

8.3.8 REPORTING OF PREGNANCY

Prior to study participation, potential female participants are counseled to use birth control to prevent pregnancy during the trial. If a female participant becomes pregnant while on study, the PIs will determine if she should continue on study medication.

8.4 UNANTICIPATED PROBLEMS

8.4.1 DEFINITION OF UNANTICIPATED PROBLEMS (UP)

If any new risks to participants are identified, the following steps may be taken:

1. Modification of inclusion/exclusion criteria
2. Suspension of new enrollment or halting of study procedures for enrolled participants
3. Modification of informed consent document and/or protocol to include an explanation of the newly recognized risks.
4. Provision of additional information about newly recognized risks to enrolled participants.

Unanticipated problems may include any incident, experience, or outcome that meets the following criteria:

1. Unexpected in nature, severity, or frequency
2. Related or possibly related to study participation
3. Research participants or others are put at a greater risk of physical, psychological, economic, or social harm not previously known.

8.4.2 UNANTICIPATED PROBLEM REPORTING

The PI will report any unanticipated problem to the IRB and to the lead PI and include the following information:

1. Protocol number and title, name of PI
2. A detailed description of the event, incident, experience, or outcome.
3. A description of any necessary changes to the protocol or other corrective actions taken or proposed.

Unanticipated problems which are SAEs will be reported to the IRB and the lead PI with 48 hours of the PI becoming aware of the event. Unanticipated problems which are not serious will be reported within 7 days of the PI becoming aware of the event. In addition, the lead PI will report the unanticipated problem to the study sponsor as appropriate.

8.4.3 REPORTING UNANTICIPATED PROBLEMS TO PARTICIPANTS

Study participants will be individually notified of unanticipated problems if there are risks to their health or to their participation in the trial.

9 STATISTICAL CONSIDERATIONS

9.1 STATISTICAL HYPOTHESES

- Primary Efficacy Endpoint(s):

For our first primary hypothesis (Aim 1, Hypothesis 1), we will evaluate the impact of the initial treatment strategy (NRT vs NRT+CM) on daily cigarette consumption (self-reported mean cigarettes per day) at 12 weeks. We hypothesize that those receiving NRT+CM will have lower daily cigarettes consumed compared to those receiving only NRT.

In those not responding to the initial treatment, our second primary hypothesis is that switching to oral medication will lead to a greater reduction in average cigarettes per day at 24 weeks when compared to intensifying CM.

- Secondary Efficacy Endpoint(s):

We hypothesize that:

1) NRT+CM will be associated with higher rates of confirmed abstinence at 12 weeks compared to NRT only.
2) Among participants who do not respond to first stage treatment, switch to oral medication will be associate with higher rates of confirmed abstinence at 24 weeks compared to participants intensifying CM.

We do not have *a priori* hypotheses about HIV outcomes (i.e., CD4, HIV viral load, VACS Index) but will estimate the impact of the sequential treatment strategies on these outcomes.

9.2 SAMPLE SIZE DETERMINATION

Power and sample size calculation. Our initial sample size calculations (see below) based on abstinence at 12 and 24 weeks required a sample size of 632 randomized participants to achieve 85% power for our 2 primary hypotheses. As a sample size of 632 is not achievable within the timeframe of this study, we have altered the primary aims by changing the primary outcome to cigarette consumption (i.e. average cigarettes per day). We now hypothesize that NRT+CM will be superior to NRT alone in ***reducing average cigarettes per day*** (H1). Similarly, in those that do not respond to first treatment (i.e. are not abstinent), we hypothesize that switching will be superior to intensifying in reducing average cigarettes per day (H2). Our baseline data shows a daily cigarette consumption of 13 (SD=7). We currently have randomized 187 participants which translates to a rate of ~8 per month. The following table demonstrates the detectable effect sizes (i.e. difference in mean number of cigarettes per day) for H1 and H2 at 80 and 90% power for different enrollment months and number of subjects enrolled per month. The calculations used a signifance level of 0.025 (two-sided), a loss to follow-up of 20% and a a non-response rate of 88.5% for H2. The sample size calculations were performed with PASS v12 (Kaysville, UT).

						1st Randomization (H1)		2nd Randomization (H2) (Non-responders)	
Remaining Enrollment Months	Enroll Rate (Subjects/ Month)	Projected N	Non-response Rate	Projected Non-responders	Lost-to-Follow-up	Detectable Cigs/Day (80% power)	Detectable Cigs/Day (90% power)	Detectable Cigs/Day (80% power)	Detectable Cigs/Day (90% power)
19	5	282	0.885	250	0.2	2.90	3.31	3.07	3.51
	6	301	0.885	266	0.2	2.80	3.20	2.98	3.41
	7	320	0.885	283	0.2	2.71	3.10	2.89	3.30
	8	339	0.885	300	0.2	2.64	3.02	2.80	3.20
	9	358	0.885	317	0.2	2.56	2.93	2.72	3.11
	10	377	0.885	334	0.2	2.50	2.86	2.65	3.03
24	5	307	0.885	272	0.2	2.78	3.17	2.94	3.36
	6	331	0.885	293	0.2	2.67	3.05	2.84	3.24
	7	355	0.885	314	0.2	2.57	2.94	2.73	3.12
	8	379	0.885	335	0.2	2.49	2.85	2.65	3.03
	9	403	0.885	357	0.2	2.41	2.76	0.04	2.93
	10	427	0.885	378	0.2	2.34	2.68	2.49	2.85

Given the expected 19 months of enrollment and anticipated 5 participants per month, we will have 80% power to detect differences for the first randomization of 2.90 cigarettes per day and 3.07 cigarettes per day for the second randomization.

Original Sample Size Calculation: The overall goal of this proposal is to identify the optimal dynamic treatment strategy for PWH who smoke cigarettes. The primary aims are to identify the most efficacious initial treatment (Aim 1, H1), verified with measurement of eCO or by next closest informant at 12 weeks, and the most efficacious treatment (switch vs intensify) for non-responders (i.e. participants who continue to smoke) to the initial randomization (Aim 2, H2), verified with measurement of eCO or by next closet informant at week 24. For the effect sizes of the treatments, VAR, combination NRT, and CM, we use effect sizes taken from a Cochrane meta-analysis of tobacco dependence treatment in PLWH, focusing on data pooled from 11 clinical trials involving a total of 1785 participants. In these studies, tobacco dependence treatment interventions (behavioral, pharmacologic, or both) had a Mantel-Haenszel risk of abstinence at up to 6 months, relative to controls, of 1.51 (95%CI 1.15, 2.00).1 An additional Cochrane meta-analysis of incentive payments for smoking cessation in general populations of adult smokers, pooling data from 17 trials involving 7715 participants, found an odds ratio for abstinence at 6 months or more of 1.42 (95%CI 1.19, 1.69), relative to controls.2 Using estimates from these studies, we assume a 12 week biochemically confirmed abstinence rate of 16% for the first-stage treatment of combination NRT+CM, and 7% for NRT alone. For the second stage, in which non-responders from both NRT and NRT+CM arms are randomized to 12 weeks of either switching medication or intensifying CM, we assume an end-of-treatment biochemically confirmed abstinence rate of 16% for switching, and 7% for intensifying. Given these parameters and a 2-sided type I error of 0.025 (corrected for 2 primary hypotheses) a total sample size of 538 participants will provide 85% power to detect superiority of NRT+CM over NRT alone for initial treatment. To account for a 15% loss to attrition, we will enroll a total of 632 participants. Given the expected proportions of non-response to initial treatment (i.e. 88.5% total; 84% and 93% in NRT+CM and NRT alone respectively), the sample size will provide 80% power to detect a difference of 9% (i.e. 16% in switch, 7% in intensify). The sample size calculation was performed with PASS v12 (Kaysville, UT). Analysis for Aim 1. Primary Study Endpoints and Analyses. The primary efficacy endpoint for this study will be biochemically verified 7-day cessation at 12 weeks (Hypothesis 1) and 24 weeks (Hypothesis 2).3 Tobacco use will be assessed by self-report and confirmatory biochemical testing with exhaled carbon monoxide.

9.3 POPULATIONS FOR ANALYSES

Intention to treat – all randomized participants will be included in the primary analysis of average cigarettes per day provided they have an assessment at either baseline and/or follow-up. In addition, all randomized participants will be included in the denominator for calculating abstinence rates with the exception of unavoidable loss to follow-up. This will include 1) participants who have died and 2) those documented to have moved to an untraceable address. We will report the number of participants in these categories who are excluded separately for each condition.4 Multiple imputation will be used to account for missing data in those who decline to be involved in subsequent data collection.

9.4 STATISTICAL ANALYSES

9.4.1 ANALYSIS OF THE PRIMARY EFFICACY ENDPOINT(S)

For our first primary hypothesis (Aim 1, Hypothesis 1), we will evaluate the impact of the initial treatment strategy (NRT vs NRT+CM) on daily cigarette consumption (self-reported average cigarettes per day) at 12 weeks. We hypothesize that those receiving NRT+CM will have lower daily cigarettes consumed compared to those receiving only NRT. A repeated measures mixed model will be used to compare the two treatment groups. The mixed model will jointly model baseline and 12-week daily cigarette consumption and will allow for the inclusion of all participants with at least one smoking assessment (Carpenter 2007). The model will include fixed effects for time and the interaction of treatment and time (i.e. no main effect of treatment) as well as age, gender and HSI. Random effects will be included for subject and site. Linear contrasts will be used to compare the difference between groups for week 12. This difference will be conditional on baseline consumption and is equivalent to an analysis adjusted for baseline (Carpenter 2007). The difference in 12 week cigarette consumption between treatment groups will be evaluated at the 0.025 significance level (i.e. Bonferroni corrected for the 2 primary hypotheses). 97.5% CIs will be estimated for treatment differences at week 12.

A similar repeated measures mixed model analysis will be used for our other primary hypothesis (Aim 1, Hypothesis 2), to evaluate the impact of the second randomization (i.e. switch vs intensify) in non-responders on average cigarettes per day. This analysis will only include participants who were non-responders to the initial treatment. For those non-responders initially randomized to NRT, switching refers to receiving VAR or bupropion in the second randomization while intensifying refers to receiving NRT+CM. For those non-responders initially randomized to NRT+CM, switching refers to receiving VAR or bupropion+CM in the second randomization while intensifying refers to receiving NRT+CM+. The repeated measures mixed model will include second stage treatment as well as covariates for site, age, gender, HSI, reason for 12-week non-response (i.e. non-abstainer vs. loss to follow-up) and initial treatment group. Differences in average cigarettes per day at 24 weeks and 97.5% confidence intervals will be estimated.

9.4.2 ANALYSIS OF THE SECONDARY ENDPOINT(S)

A logistic regression will be used including initial treatment (NRT vs NRT+CM) along with covariates for site, age, gender and HSI. Odds ratios, predicted probabilities of 12 week abstinence/reduction in smoking and 97.5% confidence intervals will be estimated. Multiple imputation by chained equations (MICE) will be used to impute missing abstinence outcomes. Baseline characteristics and abstinence outcomes assessed at other timepoints will be used in the missing data model. The difference between probabilities of 12 week abstinence/reduction between NRT and NRT+CM will be evaluated at the 0.025 two-sided significance level (i.e. Bonferroni corrected for the 2 primary hypotheses).

A similar analysis will be used for to evaluate the impact of the second randomization (i.e. switch vs intensify) in non-responders abstinence at 24 weeks. This analysis will only include participants who were non-responders to the initial treatment. For those non-responders initially randomized to NRT, switching refers to receiving VAR or bupropion in the second randomization while intensifying refers to receiving NRT+CM. For those non-responders initially randomized to NRT+CM, switching refers to receiving VAR or bupropion+CM in the second randomization while intensifying refers to receiving NRT+CM+. Logistic regression will include second stage treatment as well as covariates for site, age,

gender, HSI, reason for 12-week non-response (i.e. non-abstainer vs. loss to follow-up) and initial treatment group. Odds ratios, predicted probabilities of 24 week abstinence and 95% confidence intervals will be estimated. Multiple imputation by chained equations (MICE) will be used to impute missing abstinence outcomes. Baseline characteristics will be used in the missing data model. The difference between probabilities of 24 week abstinence between switching and intensifying will be evaluated at the 0.05 two-sided significance level.

For secondary outcomes of CD4 cell count, HIV viral load and VACS index weighted linear mixed models will be used to estimate mean outcomes at 12 and 24 weeks for each of the treatment strategies. A random effect will be included for repeated measures and the analysis will include indicators for first and second randomization, time, the interactions of first/second randomization indicators with time as well as covariates for baseline outcome, site, age and gender. Inverse probability weighting will be used to account for non-responders being re-randomized. Least squares means and 95% confidence intervals will be estimated. Evaluation of initial treatment strategies at 12 and 24 weeks will be performed using linear mixed models. These analyses will include fixed effects for initial treatment, time and their interaction as well as covariates for baseline outcome, site, age and gender and a random effect for subject to accommodate repeated measures. Similar linear mixed models of only non-responders will be used to evaluate second stage treatment.

9.4.3 SAFETY ANALYSES

To assess safety of treatment interventions, we will describe adverse events by treatment group and compare by treatment group. We will additionally examine self-reported bothersome symptoms based on response to the HIV Symptom Index, numbers of hospitalizations and ED visits overall and by treatment group.

9.4.4 BASELINE DESCRIPTIVE STATISTICS

Randomization adequacy will be assessed by comparing the differences of baseline demographic and clinical characteristics among intervention groups.

9.4.5 PLANNED INTERIM ANALYSES

No interim looks are planned.

9.4.6 SUB-GROUP ANALYSES

To examine whether treatment differences vary among subgroups, we will conduct exploratory

9.4.7 AND HYPOTHESIS GENERATING ANALYSES. WE WILL STUDY WHETHER TREATMENT EFFECTS DIFFER BY DEMOGRAPHIC AND CLINICAL CHARACTERISTICS COMMON AMONG PWH AND KNOWN TO BE ASSOCIATED WITH TOBACCO USE: DEPRESSION, ALCOHOL USE.TABULATION OF INDIVIDUAL PARTICIPANT DATA

N/A

9.4.8 EXPLORATORY ANALYSES

Exploratory Analysis. We will determine the overall best strategy with regard to 24-week abstinence. There are four strategies to compare: 1) NRT followed by VAR or bupropion in non-responders; 2) NRT followed by NRT+CM in non-responders; 3) NRT+CM followed by VAR or bupropion+CM in non-responders; 4) NRT+CM followed by NRT+CM+ in non-responders. Choosing the optimal strategy is an estimation rather than hypothesis testing problem. Weighted logistic regression will be used to estimate 24-week abstinence rates for each of the strategies. Inverse probability weighting will account for non-

responders being re-randomized and thus split in two groups as they will be underrepresented relative to responders. The analysis will include parameters for the initial randomization group as well as site, age, gender and HSI as baseline covariates. Predicted probabilities and 95% confidence intervals will be estimated for each of the strategies. The strategy with the highest predicted probability will be identified as the optimal strategy.

We will additionally examine "dose response effects" and examine the impact of number of completed interventions sessions as measure of intensity of intervention on outcomes.7

We will conduct exploratory analyses by subgroups (see 9.4.6).

10 SUPPORTING DOCUMENTATION AND OPERATIONAL CONSIDERATIONS

10.1 REGULATORY, ETHICAL, AND STUDY OVERSIGHT CONSIDERATIONS

10.1.1 INFORMED CONSENT PROCESS

Where and When Consent Will Be Obtained
For aims 1 and 2, patients will be consenting in person in the clinic where they have been recruited from or remotely. Due to social distancing requirements and safety protocols, if the situation requires, participant may be consented via phone or HIPAA compliant video conferencing with Zoom. Participants can either mail the signed consent or bring back a signed copy in person within two weeks of the phone call/video call or email it to research assistant.

For aim 3, for the survey, clinicians, staff, clinical leadership, social workers, psychologists, and care coordinators will be located wherever they have access to email and the online REDCap survey. They will not be providing any health identifiers and thus there will be no need for HIPAA authorization. They will only need to provide consent prior to study activities. The consent document will be located on the very first page of the survey; participants will not be allowed to proceed unless they agree to the terms of consent.

Waiting Period for Obtaining Consent
Participants will have as much time as needed to thoroughly review the consent form.

10.1.1.1 CONSENT/ASSENT AND OTHER INFORMATIONAL DOCUMENTS PROVIDED TO PARTICIPANTS

Script for public service announcement (SUNY Downstate site only)
MyChart script change (Yale site only)
Recruitment letter change (Yale site only)
Letters for participants lost to follow up (all sites)

10.1.1.2 CONSENT PROCEDURES AND DOCUMENTATION

Informed consent is a process that is initiated prior to the individual's agreeing to participate in the study and continues throughout the individual's study participation. Consent forms will be Institutional Review Board (IRB)-approved and the participant will be asked to read and review the document. The investigator will explain the research study to the participant and answer any questions that may arise. A verbal explanation will be provided in terms suited to the participant's comprehension of the purposes, procedures, and potential risks of the study and of their rights as research participants. Participants will have the opportunity to carefully review the written consent form and ask questions prior to signing. The participants should have the opportunity to discuss the study with their family or surrogates or think about it prior to agreeing to participate. The participant will sign the informed consent document prior to any procedures being done specifically for the study. Participants must be informed that participation is voluntary and that they may withdraw from the study at any time, without prejudice. A copy of the informed consent document will be given to the participants for their records. The informed consent process will be conducted and documented in the source document (including the date), and the form signed, before the participant undergoes any study-specific procedures. The rights and welfare of the participants will be protected by emphasizing to them that the quality of their medical care will not be adversely affected if they decline to participate in this study.

10.1.2 STUDY DISCONTINUATION AND CLOSURE

This study may be temporarily suspended or prematurely terminated if there is sufficient reasonable cause. Written notification, documenting the reason for study suspension or termination, will be provided by the suspending or terminating party to study participants, investigator, funding agency, and regulatory authorities. If the study is prematurely terminated or suspended, the Principal Investigator (PI) will promptly inform study participants, the Institutional Review Board (IRB), and sponsor and will provide the reason(s) for the termination or suspension. Study participants will be contacted, as applicable, and be informed of changes to study visit schedule.

Circumstances that may warrant termination or suspension include, but are not limited to:

- Determination of unexpected, significant, or unacceptable risk to participants
- Demonstration of efficacy that would warrant stopping
- Insufficient compliance to protocol requirements
- Data that are not sufficiently complete and/or evaluable
- Determination that the primary endpoint has been met
- Determination of futility

Study may resume once concerns about safety, protocol compliance, and data quality are addressed, and satisfy the sponsor and IRB.

10.1.3 CONFIDENTIALITY AND PRIVACY

Participant confidentiality and privacy is strictly held in trust by the participating investigators, their staff, and the sponsor(s) and their interventions. This confidentiality is extended to cover testing of biological samples in addition to the clinical information relating to participants. Therefore, the study protocol, documentation, data, and all other information generated will be held in strict confidence. No

information concerning the study or the data will be released to any unauthorized third party without prior written approval of the sponsor.

All research activities will be conducted in as private a setting as possible.

The study monitor, other authorized representatives of the sponsor, representatives of the Institutional Review Board (IRB) or other regulatory agencies may inspect all documents and records required to be maintained by the investigator, including but not limited to, medical records (office, clinic, or hospital) and pharmacy records for the participants in this study. The clinical study site will permit access to such records.

The study participant's contact information will be securely stored at each clinical site for internal use during the study. At the end of the study, all records will continue to be kept in a secure location for as long a period as dictated by the reviewing IRB, Institutional policies, or sponsor requirements.

Study participant research data, which is for purposes of statistical analysis and scientific reporting, will be transmitted to and stored at the Yale Coordinating Center. This will not include the participant's contact or identifying information. Rather, individual participants and their research data will be identified by a unique study identification number. The study data entry and study management systems used by clinical sites and by the Yale Coordinating Center research staff will be secured and password protected. At the end of the study, all study databases will be de-identified and archived at the Yale Coordinating Center.

Certificate of Confidentiality

To further protect the privacy of study participants, a Certificate of Confidentiality will be issued by the National Institutes of Health (NIH). This certificate protects identifiable research information from forced disclosure. It allows the investigator and others who have access to research records to refuse to disclose identifying information on research participation in any civil, criminal, administrative, legislative, or other proceeding, whether at the federal, state, or local level. By protecting researchers and institutions from being compelled to disclose information that would identify research participants, Certificates of Confidentiality help achieve the research objectives and promote participation in studies by helping assure confidentiality and privacy to participants.

10.1.4 FUTURE USE OF STORED SPECIMENS AND DATA

Data collected for this study will be analyzed and stored at Yale University. After the study is completed, the de-identified, archived data will be transmitted to and stored at the Yale University, for use by other researchers including those outside of the study. Permission to transmit data to the Yale University will be included in the informed consent.

eCO biological specimens will be collected but not stored.

How PHI Will Be Protected from Improper Use or Disclosure
Any identifiable information that is obtained in connection with this study will be disclosed only with participant permission or as required by U.S. or State law. Individually identifiable health information will be protected in accordance with the Health Insurance Portability and Accountability Act of 1996.

Upon completion of the study, all computerized participant datasets will be coded and stored in a password-protected study computer, to which only the MPIs and study personnel will have access. Any paper files with participant information will remain in locked files in the study office of the Project Director, until they are destroyed, after all analyses are complete and after the federal requisite waiting period (7 years) to maintain records. Any records containing PHI will be stored separately from coded data during storage, use, and transmission. Data will be collected on computers at study sites using REDCap. REDCap data will be uploaded live to a Yale maintained university server in encrypted fashion. This is a live connection; therefore, all data are immediately stored on the secure server. The server is backed up hourly. This system is compliant with HIPAA and regulations for the collection of ePHI. Computers are encrypted with PGP software.

PHI Will Be Destroyed at the Earliest Opportunity Consistent with the Research
Yes

When and How PHI Will Be Destroyed
Any paper files with participant information will remain in locked files in the study office of the Project Director, until they are destroyed, after all analyses are complete and after the federal requisite waiting period (7 years) to maintain records.

PHI Will Be Shared Yes

Description of PHI that Will Be Shared
Date of services such as clinic visit, counseling, lab results and measurements will be recorded and shared with participating research collaborators.

Justification for Sharing PHI
This information is necessary to provide a timeline and understanding of when these assessments took place, relative to the designated time points of the study.

With Whom Directly PHI Will Be Shared
Approved research personnel will have access to this information - Investigators, clinical research coordinators, biostatisticians.

PHI Can Be Obtained By Members of the Research Team,
Researchers at Mount Sinai, Researchers Outside Mount Sinai

10.1.5 KEY ROLES AND STUDY GOVERNANCE

Principal Investigator	Medical Monitor
Keith M Sigel, MD, PhD	
Mount Sinai Hospital	
1 Gustave L. Levy Place, Box 1087 NY, NY 10029	
212-825-7558	
keith.sigel@mssm.edu	

10.1.6 SAFETY OVERSIGHT

Safety oversight will be under the direction of a Data and Safety Monitoring Board (DSMB) composed of individuals with the appropriate expertise, including experts in clinical trials for addiction and substance use disorders as well as experts in statistical analysis of clinical trials in substance use disorders. Members of the DSMB are independent from the study conduct and free of conflict of interest, or measures should be in place to minimize perceived conflict of interest. The DSMB will meet at least semiannually to assess safety and efficacy data on each arm of the study. The DMSB will operate under the rules of an approved charter that will be written and reviewed at the organizational meeting of the DSMB. At this time, each data element that the DSMB needs to assess will be clearly defined. The DSMB will provide its input to NCI.

10.1.7 CLINICAL MONITORING

The Yale Coordinating Center will conduct monitoring visits to each study site on an annual basis. The MPIs, CM consultant, and Project Manager will meet with site PIs, interventionists, and research assistants at each site. The Project Manager will review the regulatory binder as well as inspect consent documents and protocol for correct versions. In addition, a random sample of participants' data will be reviewed in detail to ensure appropriate data capture. The PIs and CM consultant will meet with the site PI and interventionists to review fidelity to the protocol, review study progress, and to make suggested changes as needed. These monitoring visits may be conducted either in-person or virtually.

10.1.8 QUALITY ASSURANCE AND QUALITY CONTROL

The investigational site will provide direct access to all trial related sites, source data/documents, and reports for the purpose of monitoring and auditing by the sponsor, and inspection by local and regulatory authorities.

Procedures for data collection, management, monitoring of quality, and analysis will include use of a computerized database system (REDCap) to monitor research activities, screening, enrollment, compliance with protocol and treatment interventions, completion of scheduled assessments, and data retrieval. Data quality will be assured by: 1) extensive training/supervision of RAs in data collection; 2) preliminary review of all assessment instruments prior to data entry and checks for completeness and coding errors; and 3) error checking statistical programs. No interim looks are planned. Monthly reports

will monitor accrual, randomization; data timeliness, quality, completeness, and overall event rates (e.g. abstinence). Error corrections will be documented.

For this study, the following individuals, funding, and/or regulatory agencies will be notified: all co-Investigators listed on the protocol, the HIC, and the National Institutes of Health.

Anticipated Circumstances of Subject Withdrawal
Participants may be withdrawn from the research without their consent if they are deemed ineligible for the study. Participants may also be withdrawn from the research if they have experienced serious or related adverse events. If any participant asks to withdraw from the study, he/she will be notified that medical record data collection will continue, but any interventional procedures may cease. If a participant asks for complete withdrawal from the study, he or she must do so by written notification to the site PI.

Primary or Secondary Safety Endpoints
The use of NRT involves minimal risk, and most risks are related to the use of the patch and discomfort at the site and difficulty sleeping. VAR's most common side effect is nausea at onset of use, which is generally manageable. The main risk with Bup is seizures, thus prescribing will be avoided in high risk individuals.

10.1.9 DATA HANDLING AND RECORD KEEPING

10.1.9.1 DATA COLLECTION AND MANAGEMENT RESPONSIBILITIES

Data collection is the responsibility of the clinical trial staff at the site under the supervision of the site investigator. The investigator is responsible for ensuring the accuracy, completeness, legibility, and timeliness of the data reported.

All source documents should be completed in a neat, legible manner to ensure accurate interpretation of data.

Hardcopies of the study visit worksheets will be provided for use as source document worksheets for recording data for each participant enrolled in the study. Data recorded in the electronic case report form (eCRF) derived from source documents should be consistent with the data recorded on the source documents.

Clinical data (including adverse events (AEs), concomitant medications, and expected adverse reactions data) and clinical laboratory data will be entered into REDCap, a 21 CFR Part 11-compliant data capture system provided by the Yale Coordinating Center>. The data system includes password protection and internal quality checks, such as automatic range checks, to identify data that appear inconsistent, incomplete, or inaccurate. Clinical data will be entered directly from the source documents.

10.1.9.2 STUDY RECORDS RETENTION

Study documents should be retained for a minimum of 2 years after the last approval of a marketing application in an International Conference on Harminosation (ICH) region and until there are no pending or contemplated marketing applications in an ICH region or until at least 2 years have elapsed since the

formal discontinuation of clinical development of the study intervention. These documents should be retained for a longer period, however, if required by local regulations. No records will be destroyed without the written consent of the sponsor, if applicable. It is the responsibility of the sponsor to inform the investigator when these documents no longer need to be retained.

10.1.10 PROTOCOL DEVIATIONS

Documenting Protocol Deviations

Medications:
If a participant does not receive the medications as part of their assigned treatment arm, this should be documented as a protocol deviation in REDCap. This may be entered once in the Protocol Deviation Form in the Baseline Forms, Week 12 Forms, Week 24 Forms, or Unscheduled Visit Forms.
This may occur for various reasons: 1) participant declines a particular medication; 2) participant did not fill a prescription for any reason; 3) incorrect medication was prescribed.

NRT: Participants should be prescribed both a long- and short-acting nicotine replacement therapy in Stage 1. If one or both were not acquired, then one protocol deviation should be submitted.
VAR: If varenicline or bupropion was not acquired when a participant was randomized to either the VAR or VAR+CM arm in Stage 2, then one protocol deviation should be submitted.

These deviations are not meant to be punitive in nature. Rather, we need a way to be able to track how often this is happening. (Medications actually taken will also be tracked via the Smoking Related Treatment Services Form)

Incorrect CM draws:
If participant is awarded an incorrect number of draws from the fishbowl, this needs to be documented as a protocol deviation.
Some examples may be:

a) Participant was awarded more than the maximum number of draws:
Maximum is 9 draws for the NRT+CM intensified group
Maximum is 5 draws for all other groups

b) Participant was awarded draws, even if they reported smoking (even a puff) in the last 7 days.
Reminder: Smokerlyzer should ONLY be used to confirm a verbal report of no smoking in the past 7 days. Draws are then awarded if eCO <= 6ppm.

c) Too many or too few draws per specific CM visit.

We will not report medication deviations related to medication non-use and incorrect CM draws (described in the above sections) as individual events to the Mount Sinai IRB to limit administrative burden as they are not indicators of human subject risk. Instead, we will present these data as aggregate to our DSMB and in our annual report.

It is the responsibility of the site Principal Investigator to use continuous vigilance to identify and report deviations within 5 working days of identification of the protocol deviation, or within 5 working days of

the scheduled protocol-required activity. All deviations must be addressed in study source documents, reported the Yale Data Coordinating Center Protocol deviations must be sent to the reviewing Institutional Review Board (IRB) per their policies. The site investigator is responsible for knowing and adhering to the reviewing IRB requirements. Further details about the handling of protocol deviations will be included in the MOP.

10.1.11 PUBLICATION AND DATA SHARING POLICY

This study will be conducted in accordance with the following publication and data sharing policies and regulations:

National Institutes of Health (NIH) Public Access Policy, which ensures that the public has access to the published results of NIH funded research. It requires scientists to submit final peer-reviewed journal manuscripts that arise from NIH funds to the digital archive PubMed Central upon acceptance for publication.

This study will comply with the NIH Data Sharing Policy and Policy on the Dissemination of NIH-Funded Clinical Trial Information and the Clinical Trials Registration and Results Information Submission rule. As such, this trial will be registered at ClinicalTrials.gov, and results information from this trial will be submitted to ClinicalTrials.gov. In addition, every attempt will be made to publish results in peer-reviewed journals. Data from this study may be requested from other researchers 7 years after the completion of the primary endpoint by contacting the lead PIs.

As study results are obtained, Drs. Edelman and Bernstein will review findings, contribute to data analysis and interpretation, co-author manuscripts and presentations for scientific meetings, draft a proposed dissemination plan (e.g., determine target journals, author contribution, and order) and receive input from the investigative team on the proposed dissemination plan with a goal of identifying appropriate venues across the fields of HIV, substance use and tobacco focused research. Dr. Edelman also will be responsible for submission of annual progress reports and will be in communication with NIH project staff throughout the study.

Consistent with NIH policy, we are planning to make the results and accomplishments of the study available to the research community and to the public at large. We will adhere to the NIH Grants Policy on “NIH Sharing Policies and Related Guidance on NIH-Funded Research Resources,” including the policies on “Sharing Clinical Trial Information,” including registration of our trial and reporting of all outcomes within ClinicalTrials.gov and work with institutional support at Yale University to ensure adherence to reporting timelines and ensure that consent procedures indicate this policy to inform potential participants. Results from the proposed research will be of interest to a wide range of audiences, including researchers in the fields of tobacco use and treatment, HIV, implementation science; service providers in HIV treatment settings; advocates for HIV treatment; and patients with HIV who smoke.

10.1.12 CONFLICT OF INTEREST POLICY

The independence of this study from any actual or perceived influence, such as by the pharmaceutical industry, is critical. Therefore, any actual conflict of interest of persons who have a role in the design, conduct, analysis, publication, or any aspect of this trial will be disclosed and managed. Furthermore,

persons who have a perceived conflict of interest will be required to have such conflicts managed in a way that is appropriate to their participation in the design and conduct of this trial. The study leadership in conjunction with the National Cancer Institute has established policies and procedures for all study group members to disclose all conflicts of interest and will establish a mechanism for the management of all reported dualities of interest.

10.2 ADDITIONAL CONSIDERATIONS

N/A

10.3 ABBREVIATIONS

AE	Adverse Event
ANCOVA	Analysis of Covariance
CFR	Code of Federal Regulations
CM	Contingency Management
CMP	Clinical Monitoring Plan
COC	Certificate of Confidentiality
CONSORT	Consolidated Standards of Reporting Trials
CRF	Case Report Form
DCC	Data Coordinating Center
DHHS	Department of Health and Human Services
DSMB	Data Safety Monitoring Board
EC	Ethics Committee
eCO	Exhaled carbon monoxide
eCRF	Electronic Case Report Forms
FDA	Food and Drug Administration
GCP	Good Clinical Practice
HIPAA	Health Insurance Portability and Accountability Act
HIS	Heaviness of Smoking Index
IB	Investigator’s Brochure
ICH	International Conference on Harmonisation
ICMJE	International Committee of Medical Journal Editors
IRB	Institutional Review Board
ISM	Independent Safety Monitor
ISO	International Organization for Standardization
ITT	Intention-To-Treat
LSMEANS	Least-squares Means
MedDRA	Medical Dictionary for Regulatory Activities
MOP	Manual of Procedures
MPI	Multiple Principal Investigators
NCT	National Clinical Trial
NIH	National Institutes of Health
NIH IC	NIH Institute or Center
NRT	Nicotine replacement therapy
OHRP	Office for Human Research Protections
PI	Principal Investigator
PWH	Persons with HIV
QA	Quality Assurance
QC	Quality Control
RCT	Randomized Controlled Trial
SAE	Serious Adverse Event
SAP	Statistical Analysis Plan
SMC	Safety Monitoring Committee
SOA	Schedule of Activities
SOP	Standard Operating Procedure
UP	Unanticipated Problem
US	United States
VAR	varenicline
VACS	Veterans Aging Cohort Study

YCCI	Yale Center for Clinical Investigation

10.4 PROTOCOL AMENDMENT HISTORY

The table below is intended to capture changes of IRB-approved versions of the protocol, including a description of the change and rationale. A Summary of Changes table for the current amendment is located in the Protocol Title Page.

Version	Date	Description of Change	Brief Rationale
1.1, 2.1, 2.2, 3, 9.1	12/22/22	Change in primary and secondary outcome measures	At the suggestion of the DSMB
9.1, 9.2, 9.3, 9.4	12/22/22	Change in statistical analysis due to changes in outcome measures and new reference added	At the suggestion of the DSMB and biostatistician
n/a	12/22/22	Extension of timeline to recruit into Year 5	To maximize recruitment, which was slowed during COVID-19 surges. (Original recruitment timeline was through Year 4.)
5.5	12/22/22	Addition of script for public service announcement to be shown in waiting room (SUNY Downstate site only)	To reach patients in the waiting room so that they are aware of the study and can refer themselves for screening
6.3	12/22/22	Sample size description to be deleted	Biostatistician discovered that it is in the wrong section
n/a	12/22/22	Changes made to the MyChart script and recruitment letter (Yale site only)	Requested by the Yale IRB
n/a	12/22/22	Letter to be sent to enrolled study participants who cannot be reached	Some participants can no longer be reached by telephone due to numbers no longer in service
4.1, 7.3	12/22/22	More specific language about texting participants in the study	Requested by Mount Sinai IRB texting will no longer be done on Google Voice as it's not HIPAA compliant and will now be using Zoom Phone

11 REFERENCES

Include a list of relevant literature and citations for all publications referenced in the text of the protocol. Use a consistent, standard, modern format, which might be dependent upon the required format for the anticipated journal for publication (e.g., N Engl J Med, JAMA, etc.). The preferred format is International Committee of Medical Journal Editors (ICMJE). Include citations to product information such as manufacturer's IB, package insert, and device labeling.

1. Pool ER, Dogar O, Lindsay RP, Weatherburn P, Siddiqi K. Interventions for tobacco use cessation in people living with HIV and AIDS. The Cochrane Library. 2016.
2. Cahill K, Hartmann#Boyce J, Perera R. Incentives for smoking cessation. Cochrane Database of Systematic Reviews. 2015(5).
3. Hughes JR, Keely JP, Niaura RS, Ossip-Klein DJ, Richmond RL, Swan GE. Measures of abstinence in clinical trials: issues and recommendations. Nicotine & Tobacco Res. 2003;5:13-25.
4. West R, Hajek P, Stead L, Stapleton J. Outcome criteria in smoking cessation trials: proposal for a common standard. Addiction. 2005;100(3):299-303.
5. Crivello ALJM, SA. Statistical Methodology for a SMART Design in the Development of Adaptive Treatment Strategies. (Tech. Rep. No. 07-82). . University Park, PA: The Pennsylvania State University, The Methodology Center.;2007.
6. Carpenter, JR and Kenward MG (2007). Missing data in randomised controlled trials - a practical guide. Birmingham: National Institute for Health Research, Publication RM03/JH17/MK. Available at *http://www.missingdata.org.uk*
7.. Pool ER, Dogar O, Lindsay RP, Weatherburn P, Siddiqi K. Interventions for tobacco use cessation in people living with HIV and AIDS. Cochrane Database Syst Rev. 2016(6):CD011120.

Mount Sinai

Icahn School of Medicine at Mount Sinai
Mount Sinai Beth Israel
Mount Sinai Brooklyn
The Mount Sinai Hospital
Mount Sinai Queens
New York Eye and Ear Infirmary
of Mount Sinai
Mount Sinai St. Luke's
Mount Sinai West

Program for the Protection of Human Subjects
Institutional Review Boards
Mount Sinai Health System
One Gustave L. Levy Place, Box 1081
New York, NY 10029-6574
T 212-824-8200
F 212-876-6789
irb@mssm.edu
icahn.mssm.edu/pphs

APPROVAL

October 23, 2024

KEITH SIGEL
2128247558
keith.sigel@mssm.edu

Dear KEITH SIGEL:

On 10/22/2024 an Institutional Review Board of the Mount Sinai School of Medicine, in accordance with Mount Sinai's Federal Wide Assurances (FWA#00005656, FWA#00005651) to the Department of Health and Human Services approved the following human subject research from 10/22/2024 to 10/21/2025 inclusive:

Type of Review:	Modification and Continuing Review
Project Title:	R2S: A SMART Approach to Treating Tobacco Use Disorder in Persons Living with HIV
Investigator:	KEITH SIGEL
Project Information:	STUDY-19-01121-MODCR002<br>Funding Source: Name: Yale University School Of Medicine, Grant Office ID: 18-2805
MSHS System Sites:	St. Luke Hospital, Mount Sinai
IND, IDE, or HDE:	None
Documents Reviewed:	• 19-01121 Protocol Clean, Category: IRB Protocol;<br>• 19-01121 ProtocolTracked, Category: IRB Protocol;<br>• Advertisement 19-01121 SMARTTT_Pen_Mount Sinai.pdf, Category: Other;<br>• Chantix insert.pdf, Category: Drug Attachment;<br>• Documentation 19-01121 Cert HIC# 2000026332 SMARTTT ICF Sinai Specific_12.9.2020_clean .docx.pdf, Category: Other;<br>• Documentation 19-01121 Certification HIC# 2000026332 SMARTTT Reminder Slips Med ONLY.DOCX.pdf, Category: Other;<br>• Documentation 19-01121 Certification HIC# 2000026332 SMARTTT Reminder Slips Medication CM.DOCX.pdf, Category: Other;<br>• Documentation 19-01121 CM Patient Education + Pharmacist Tips, Category: Other;

	• Documentation 19-01121 MedicationCM_ReminderSlip_Sinai10.22.2020.docx, Category: Other;<br>• Documentation 19-01121 MedicationOnly_ReminderSlip_Sinai10.22.2020.docx, Category: Other;<br>• Documentation 19-01121 Proposed added question in REDCap.docx, Category: Validated questionnaires / evaluation instruments;<br>• Documentation 19-01121 Template MedicationCM_ReminderSlip.pdf, Category: Other;<br>• Documentation 19-01121 Template MedicationOnly_ReminderSlip.pdf, Category: Other;<br>• Documentation 19-01121 Translated HIC# 2000026332 SMARTTT Reminder Slips Medication CM.docx, Category: Other;<br>• Documentation 19-01121 Translated HIC# 2000026332 SMARTTT Reminder Slips Medication ONLY.doc, Category: Other;<br>• Documentation 19-01121Certification HIC# 2000026332 Tobacco Treatment HIV Study Mount Sinai.docx.pdf, Category: Other;<br>• DSMB Letter 19-09211 7.12.21, Category: Other;<br>• Flyer 19-01121 Smoking Flyer_03.10.2020_clean.pdf, Category: Recruitment Materials;<br>• Flyer 19-01121 Tobacco Treatment HIV General Template Flyer final.pdf, Category: Recruitment Materials;<br>• Flyer 19-01121 Tobacco Treatment HIV -Mount Sinai_finalpdf_6.30.21 edit.pdf, Category: Recruitment Materials;<br>• Flyer 19-01121 Translated Tobacco Treatment HIV Study Mount Sinai_CCedit_7.12.21.pdf, Category: Recruitment Materials;<br>• FW R2S - Moving sites from Ideate to RUTH.htm, Category: Other;<br>• HIPAA_waiver_authorization_SMARTTT_v3_AS.pdf, Category: Other;<br>• HRP-212 - FORM - Continuing Review Progress 12.19.19_SINAI_FINAL.doc, Category: Other;<br>• HRP-388, Category: Other;<br>• HRP-410 - CHECKLIST - Waiver or Alteration of Consent Process 11.24.2020.docx, Category: Other;<br>• HRP-503 Full Version 19-01121 5.17.23, Category: Other;<br>• Implementation Survey SMARTTT_FINAL.docx, Category: Validated questionnaires / evaluation instruments;

	• Main ICF 19-01121 Translated SMARTTT ICF Sinai Specific_7.9.2021_clean.pdf, Category: Consent Form;<br>• Main ICF 19-01121_new 2022 template, Category: Consent Form;<br>• Memo regarding protocol changes 19-01121 7.12.21, Category: Other;<br>• nicorette insert.pdf, Category: Drug Attachment;<br>• nicotine lozenge insert.pdf, Category: Drug Attachment;<br>• nicotine patch insert.pdf, Category: Drug Attachment;<br>• Protocol 19-01121 Template-12.22.22_tracked, Category: Other;<br>• Protocol 19-01121 Template-SMARTTT_04-27-2021_clean.docx, Category: Other;<br>• Research Info Sheet-Aim 3_second survey 08012024_Sinai specific.pdf, Category: Consent Form;<br>• Research Info Sheet-Aim 3_second survey 08012024_template.pdf, Category: Consent Form;<br>• Sinai Specific Hard to Reach Participants Letter 19-01121 , Category: Recruitment Materials;<br>• Sinai Specific ICF 19-01121_new 2022 template, Category: Consent Form;<br>• Sinai Specific In-Person Screening Form_Sinai_Clean.pdf, Category: Recruitment Materials;<br>• Sinai Specific Patient Recruitment Letter_No Change_Sinai_Clean.pdf, Category: Recruitment Materials;<br>• Sinai Specific Sinai Smoking Flyer_07.28.2020_clean.pdf, Category: Recruitment Materials;<br>• Sinai Specific SMART English Phone Screener_Sinai_Clean.pdf, Category: Recruitment Materials;<br>• SMARTTT Video Content- Clinic Recruitment, Category: Recruitment Materials;<br>• Statement of Work - Mount Sinai - FINAL.docx, Category: Other;<br>• Study 19-01121 HRP235A Transition to 2022 Consent form_signed, Category: Other;<br>• Study 19-01121 HRP-235B Sinai Notification Plan to 2022 consent form_signed, Category: Other;<br>• Template InPerson Screening Form 19-01121_07.28.2020_clean.pdf, Category: Recruitment Materials;<br>• Template Patient Recruitment Letter 19-01121 03.10.2020_clean.pdf, Category: Recruitment Materials;<br>• Template SMART English Phone Screener_03.10.2020_clean.docx.pdf, Category: Recruitment Materials;<br>• TSR modified FU.docx, Category: Other;

	• zyban bupropion insert.pdf, Category: Drug Attachment;

• The request for a waiver of informed consent was approved for the specified procedures described in the protocol.

The PHI for which access and use has been granted for this project [which are the minimum necessary] include the following

Identifiers:

Name

Date of Birth

Medical Number

Telephone Number

Dates of Service

Address/zipcode

Health Information:

For aim 1/2, electronic medical records, lab results, questionnaire answers, responses on measures, will all be used to provide data about the subjects.

Primary Care provider records:

- HIV/AIDs status, hepatitis infection status, sexually transmitted diseases, physical exam (height, weight, blood pressure), diagnosis and treatment of medical and psychiatric conditions, sexual practices, tobacco and alcohol use, use of illegal drugs, smoking status and habits, medication list, pregnancy status, language abilities, adverse events, side effects, transportation, time/costs travel to clinic.

Specialist provider records:

- HIV/AIDs status, hepatitis infection status, sexually transmitted diseases, physical exam (height, weight, blood pressure), diagnosis and treatment of medical and psychiatric conditions, sexual practices, tobacco and alcohol use, use of illegal drugs, smoking status and habits, medication list, adverse events, side effects, transportation, time/ costs travel to clinic

Laboratory records and other test results:

-CD4 count, hepatitis infection status, sexually transmitted disease, viral load, creatinine, hemoglobin, WBC, eGFR, albumin, platelets, AST, ALT, Body-mass index.

Psychiatrist/therapy records:

- HIV/AIDs status, hepatitis infection status, sexually transmitted diseases, diagnosis and treatment of medical and

psychiatric conditions, sexual practices, tobacco and alcohol use, use of illegal drugs, smoking status and habits,

medication list, adverse events, side effects, transportation, time/costs travel to clinic

Phone call records:

- diagnosis and treatment of medical and psychiatric conditions, adverse events, side effects, transportation, time/

costs to travel to clinic

Demographics from electronic medical record:

-First name, last name, gender, date of birth, MRN, telephone number, employment status, insurance, income, self-identified gender identity, race, ethnicity, education, mailing address, proxies/alternate contacts, transportation, time/costs travel to clinic.

Requesting Waiver for Aim 3 of study procedures involving clinicians, staff and clinical leadership. Waiver is requested to perform pre and post trial surveys. Survey data will be collected in a de-identified fashion. No health identifiers will be collected. An information sheet will be used.

• The request for a waiver of HIPAA authorization was approved. The PHI for which access and use has been granted for this project [which are the minimum necessary] include the following:

Identifiers:

Name
Date of Birth
Medical Number
Telephone Number
Dates of Service
Address/zipcode

Health Information:

For aim 1/2, electronic medical records, lab results, questionnaire answers, responses on measures, will all be used to provide data about the subjects.

Primary Care provider records:
- HIV/AIDs status, hepatitis infection status, sexually transmitted diseases, physical exam (height, weight, blood pressure), diagnosis and treatment of medical and psychiatric conditions, sexual practices, tobacco and alcohol use, use of illegal drugs, smoking status and habits, medication list, pregnancy status, language abilities, adverse events, side effects, transportation, time/costs travel to clinic.

Specialist provider records:
- HIV/AIDs status, hepatitis infection status, sexually transmitted diseases, physical exam (height, weight, blood pressure), diagnosis and treatment of medical and psychiatric conditions, sexual practices, tobacco and alcohol use, use of illegal drugs, smoking status and habits, medication list, adverse events, side effects, transportation, time/ costs travel to clinic

Laboratory records and other test results:
-CD4 count, hepatitis infection status, sexually transmitted disease, viral load, creatinine, hemoglobin, WBC, eGFR, albumin, platelets, AST, ALT, Body-mass index.

Psychiatrist/therapy records:
- HIV/AIDs status, hepatitis infection status, sexually transmitted diseases, diagnosis and treatment of medical and
psychiatric conditions, sexual practices, tobacco and alcohol use, use of illegal drugs, smoking status and habits,
medication list, adverse events, side effects, transportation, time/costs travel to clinic

Phone call records:
- diagnosis and treatment of medical and psychiatric conditions, adverse events, side effects, transportation, time/
costs to travel to clinic

Demographics from electronic medical record:
-First name, last name, gender, date of birth, MRN, telephone number, employment status, insurance, income, self-identified gender identity, race, ethnicity, education, mailing address, proxies/alternate contacts, transportation, time/costs travel to clinic.

Requesting Waiver for Aim 3 of study procedures involving clinicians, staff and clinical leadership. Waiver is requested to perform pre and post trial surveys. Survey data will be collected in a de-identified fashion. No health identifiers will be collected. An information sheet will be used.

IRB approval requires that this research is conducted in full compliance with the requirements indicated in the PPHS/IRB Investigator Manual. Additionally, all required local committee approvals at each research affiliate site must be obtained prior to study initiation.

It is imperative to recognize that IRB approval does not constitute or imply institutional support for the conduct of this research. Further, IRB approval from Mount Sinai is provided with the understanding that the investigator and research team will strictly adhere to all laws and regulations governing research in the localities where the project is to be conducted.

To access stamped consent forms, log into RUTH, search for your project in the IRB, Active tab, and click the Documents tab in the study workspace.

To avoid rejection of your manuscript by a journal and/or non-compliance with federal requirements, you must register and update your study on ClinicalTrials.gov via the ISMMS Office of Research Services (ORS) ticket Clinicaltrials.gov Assistance. For ICMJE studies, registration must occur prior to the first enrolled subject in your study and to meet federal requirements registration must be completed within 21 days after the first enrollment.

As a courtesy reminder, in order to request continuing IRB approval or study closure, you are required to submit a Continuing/Final Review Progress Report and required attachments at least six weeks prior to your project's expiration date. If IRB continuing review approval is not granted before the expiration date of 10/21/2025, IRB approval of this research expires on that date. There is no grace period beyond one year from the last approval date. It is your responsibility to submit your research protocol for continuing review.

If your project qualified for an extended expedited expiration, and was given an approval period of more than one year, please note that the elimination of annual continuing reviews does not remove the responsibility of the PI/Research Team to monitor the ongoing conduct of the research. The rules as they apply to reporting new information, requesting modifications to the protocol or consent and making final reports have not changed. CITI refresher training for research staff must still be completed every three years, and financial conflicts of interest must be managed by the FCOI committee independent of the project approval period. In addition, any other changes by the PPHS, ISMMS, or federal or state regulations that affect projects, e.g. consent language changes or new policy, must still be implemented during the three-year period.

For questions, please contact the IRB staff member working on the project or the PPHS at 212-824-8200.
