# Supplementary material for: Clinical Pharmacists, Medications, and Contingency Management for Targeting Smoking in HIV Clinics: A Randomized Clinical Trial
Source: JAMA Netw Open. 2026 Feb 27;9(2):e2560593. doi: 10.1001/jamanetworkopen.2025.60593 (PMC12949440; doi:10.1001/jamanetworkopen.2025.60593)
Supplement: Supplement 2. — eTable 1. Baseline characteristics of participants by stage 2 treatment eTable 2. Effects of adaptive strategies on cigarettes per day and abstinence at week 12 and week 24 (strategies n=323) eTable 3. Number of participants experiencing adverse events in stage 1 and stage 2 treatment by group eFigure 1. Clinical pharmacist visit completion by stage and treatment group eFigure 2. MTUD prescription at first visit by stage, treatment group, and medication eAppendix. Medications with contingency management for treating tobacco use disorder in persons with HIV—Trial intervention training manual [file jamanetwopen-e2560593-s002.pdf]

Supplementary Online Content

Edelman EJ, Deng Y, Dziura J, et al. Clinical pharmacists, medications, and contingency management for targeting smoking in HIV clinics: a randomized clinical trial. *JAMA Netw Open*. 2026;9(2):e2560593. doi:10.1001/jamanetworkopen.2025.60593

eTable 1. Baseline characteristics of participants by stage 2 treatment
eTable 2. Effects of adaptive strategies on cigarettes per day and abstinence at week 12 and week 24 (strategies n=323)
eTable 3. Number of participants experiencing adverse events in stage 1 and stage 2 treatment by group
eFigure 1. Clinical pharmacist visit completion by stage and treatment group
eFigure 2. MTUD prescription at first visit by stage, treatment group, and medication
eAppendix. Medications with contingency management for treating tobacco use disorder in persons with HIV—Trial intervention training manual

This supplementary material has been provided by the authors to give readers additional information about their work.

© 2026 Edelman EJ et al. *JAMA Network Open*.

eTable 1. Baseline characteristics of participants by stage 2 treatment

	Number (%) of participants			
**Characteristic**	**NRT alone continue (n=16)**	**NRT+CM continue (n=36)**	**Switch (NRT→Oral MTUD and NRT+CM→oral MTUD+CM) (n=130)**	**Intensify (NRT→CM and NRT+CM→NRT+CM+) (n=136)**
**Sociodemographic characteristics**				
Age, mean (SD), y	55.8 (11.0)	55.0 (11.6)	55.2 (9.6)	54.7 (11.6)
Sex at birth				
Male	12/16 (75.0%)	24/36 (66.7%)	65/130 (50.0%)	77/136 (56.6%)
Female	4/16 (25.0%)	12/36 (33.3%)	65/130 (50.0%)	59/136 (43.4%)
Sex at baseline				
Male	12/16 (75.0%)	23/35 (65.7%)	66/130 (50.8%)	75/136 (55.2%)
Female	4/16 (25.0%)	12/35 (34.3%)	63/130 (48.5%)	59/136 (43.4%)
Transgender	0/16 (0.0%)	1/159 (0.6%)	1/130 (0.8%)	2/136 (1.5%)
Race, No (%)				
American Indian or Alaska Native	1/16 (6.25%)	2/35 (5.7%)	4/129 (3.1%)	1/132 (0.8%)
Asian	0/16 (0.0%)	1/35 (2.9%)		0/132 (0.0%)
Black or African American	11/16 (68.8%)	26/35 (74.3%)	93/129 (72.1%)	99/132 (75.0%)
Native Hawaiian or Other Pacific Islander	0/159 (0.0%)	0/35 (0%)	1/129 (0.8%)	0/132 (0.0%)
White	3/16 (18.8%)	6/35 (17.1%)	21/129 (16.3%)	29/132 (22.0%)
Mixed Race	1/16 (6.3%)	6/158 (3.8%)	10/129 (7.8%)	33/132 (2.3%)
Hispanic, Latino/a	2/16 (12.5%)	7/36 (19.4%)	34/130 (26.2%)	30/135 (22.2%)
Sexuality				
Lesbian or gay	5/16 (31.3%)	8/36 (22.2%)	19/128 (14.8%)	27/135 (20.0%)
Straight	6/16 (37.5%)	24/36 (66.7%)	92/128 (71.9%)	96/135 (71.1%)
Bisexual	4/16 (25.0%)	3/36 (8.3%)	13/128 (10.2%)	7/135 (5.2%)

© 2026 Edelman EJ et al. *JAMA Network Open*.

Something else	1/16 (6.3%)	1/36 (2.8%)	4/128 (3.1%)	5/135 (3.7%)
Marital status				
Married/In a civil union or domestic Partnership	1/16 (6.3%)	4/36 (11.1%)	19/130 (14.6%)	23/136 (16.9%)
Divorced	2/16 (12.5%)	6/36 (16.7%)	13/130 (10.0%)	24/136 (17.7%)
Separated	2/16 (12.5%)	3/36 (8.3%)	11/130 (8.5%)	9/136 (6.6%)
Widowed	0/16 (6.8%)	3/36 (8.3%)	9/130 (6.9%)	9/136 (6.6%)
Never married	11/16 (68.8%)	20/36 (55.6%)	73/130 (56.2%)	71/136 (52.2%)
Housing insecurity, past 12 months	5/16 (31.3%)	7/36 (19.4%)	19 (14.6%)	18/136 (13.2%)
Food insecurity, past 12 months	4/16 (25.0%)	7/36 (19.4%)	26 (20.0%)	19/136 (14.0%)
Medication insecurity, past 12 Months	2/16 (12.5%)	3/36 (8.3%)	8/124 (6.5%)	7/125 (5.6%)
Currently employed	1/16 (6.3%)	8/36 (22.2%)	21/124 (16.9%)	27/134 (20.2%)
Highest level of education				
Elementary school	0/16 (0.0%)	0/36 (0%)	4/129 (3.1%)	8/136 (5.9%)
Some high school	3/16 (18.8%)	9/36 (25.0%)	30/129 (23.3%)	30/136 (22.1%)
High school graduate	8/16 (50.0%)	10/36 (27.8%)	46/129 (35.7%)	43 (31.6%)
Some college or technical school	5/16 (31.3%)	10/36 (27.8%)	34/129 (26.4%)	41/136 (30.2%)
College graduate or more	0/16 (0.0%)	7/36 (19.4%)	15/129 (11.6%)	14/136 (10.3%)
Have any health insurance	16/16 (100.0%)	36/36 (100.0%)	130/130 (100.0%)	135/135 (100.0%)
Public[a]				
Medicaid	13/16 (81.3%)	32/36 (88.9%)	105/130 (80.8%)	110/134 (82.1%)
Medicare	5/16 (31.3%)	16/36 (44.4%)	40/129 (31.0%)	50/132 (37.9%)
Other public insurance	3/16 (20.0%)	8/36 (22.2%)	25/130 (19.2%)	30/134 (22.4%)
Private	4/16 (25.0%)	36/36 (100%)	16/128 (12.5%)	16/134 (11.9%)
Ryan White HIV/AIDS program or AIDS Drug Assistance Program	5/15 (33.3%)	11/36 (30.6%)	24/128 (18.8%)	28/134 (20.9%)
Mode of usual transportation				
Drive self	0/16 (0.0%)	6/35 (17.1%)	19/124 (15.6%)	19/129 (14.7%)
Someone else drives	2/16 (12.5%)	2/35 (5.7%)	14/124 (11.5%)	15/129 (11.6%)
Clinic van	4/16 (25.0%)	1/35 (2.9%)	22/124 (18.0%)	12/129 (9.3%)

© 2026 Edelman EJ et al. *JAMA Network Open*.

Bus	4/16 (25.0%)	6/35 (17.1%)	24/122 (19.7%)	35/129 (27.1%)
Subway	4/16 (25.0%)	10/35 (28.6%)	17/124 (13.9%)	24/129 (18.6%)
Walk	1/16 (6.3%)	6/35 (17.1%)	15/124 (12.3%)	15/129 (11.6%)
Other	1/16 (6.3%)	4/35 (11.4%)	11/124 (9.0%)	9/129 (7.0%)
**Smoking characteristics**				
Smoking pattern				
Smokes every day	16/16 (100.0%)	35/36 (97.2%)	130130 100.0%)	135/136 (99.3%)
Smokes some days	0/16 (0.0%)	1/36 (2.8%)	0/130 (0.0%)	1 /136(0.7%)
eCO, median (IQR), ppm	9.0 (2.0 – 14.5)	10.5 (4.0 – 14.0)	10.0 (6.5 – 16.0)	10.0 (7.0 - 16.0)
Cigarettes per day, median (IQR)	10.0 (6.5 – 12.0)	10.0 (7.0 – 15.0)	10.0 (8.0 – 20.0)	10.0 (7.0 – 20.0)
HSI, median (IQR), total score[b]	2.5 (1.0 – 3.0)	2.0 (1.0 – 3.0)	3.0 (2.0 – 4.0)	3.0 (2.0 – 3.5)
Readiness to quit, median (IQR)[c]	9.5 (8.5 – 10.0)	9.0 (8.0 – 10.0)	9.0 (7.0 – 10.0)	8.5 (7.0 – 10.0)
Relapse risk score[d], median (IQR)	6.5 (4.0 – 7.5)	7.0 (5.0 – 7.0)	7.0 (5.0 – 8.0)	7.0 (5.0 – 8.0)
Type of cigarettes usually smoked				
Menthol	14/16 (87.5%)	27 (75.0%)	109/130 (83.9%)	117/136 (86.0%)
Non-menthol	1/16 (6.3%)	8 (22.2%)	16/130 (12.3%)	18/136 (13.2%)
No usual type	1/16 (6.3%)	1 (2.8%)	5/130 (3.9%)	1/136 (0.7%)
E-cigarette or other electronic vaping products use				
Some days or every day	2/16 (12.5%)	4/36 (11.1%)	10/130 (7.7%)	11/134 (8.2%)
Prior use, not at all currently	4/16 (25.0%)	5/36 (13.9%)	21/130 (16.2%)	20/134 (14.9%)
Never used	10/16 (62.5%)	27/36 (75.0%)	99/130 (76.2%)	103/134 (76.9%)
**Non-medical substance use, past 3 months[e]**				
Alcohol use				
Low risk	14/16 (87.5%)	32/36 (88.9%)	114/130 (87.7%)	120/136 (88.2%)
Medium risk	2/16 (12.5%)	3/36 (8.3%)	8/130 (6.2%)	13/136 (9.6%)
High risk	0/16 (0.0%)	1/36 (2.8%)	8/130 (6.2%)	3/136 (2.2%)
Cannabis use				
Low risk	9/16 (56.3%)	26/36 (72.2%)	92/130 (70.8%)	98/136 (72.1%)
Medium risk	7/16 (43.8%)	10/36 (27.8%)	34/130 (26.2%)	32/136 (23.5%)

© 2026 Edelman EJ et al. *JAMA Network Open*.

High risk	0/16 (0.0%)	0/36 (0%)	4/130 (3.1%)	6/136 (4.4%)
Stimulant use				
Low risk	16/16 (100.0%)	32/36 (88.9%)	119/130 (91.5%)	119/136 (87.5%)
Medium risk	0/16 (0.0%)	3/36 (8.3%)	7/130 (5.4%)	6/136 (4.4%)
High risk	0/16 (0.0%)	1/36 (2.8%)	4/130 (3.1%)	11/136 (8.1%)
Sedative or sleeping medication use				
Low risk	14/16 (87.5%)	32/36 (88.9%)	129/130 (99.2%)	132/136 (97.1%)
Medium risk	2/16 (12.5%)	3/36 (8.3%)	1/130 (0.8%)	4/136 (2.9%)
High risk	0/16 (0%)	1/36 (2.8%)	0/130 (0%)	0/136 (0%)
Opioid use				
Low risk	16/16 (100.0%)	36/36 (100%)	129/130 (99.2%)	134/136 (98.5%)
Medium risk	0/16 (0.0%)	0/36 (0%)	1/130 (0.8%)	2/136 (1.5%)
High risk	0/16 (0.0%)	0/36 (0%)	0/130 (0%)	0/136 (0%)
Depressive symptoms, moderate-severe[f]	3 (18.8%)	6 (17.1%)	28 (22.4%)	25 (18.5%)
**HIV-related measures**				
Prescribed antiretroviral therapy	16/16 (100.0%)	35/36 (97.2%)	127/130 (97.7%)	132/134 (97.1%)
HIV viral load, detectable	1/16 (6.3%)	3/36 (8.3%)	16/128 (12.5%)	16/134 (11.9%)
CD4 cell count, cells/mm^3, median (IQR)	653.0 (343.5 – 986.5)	511.0 (355.5 – 796.0)	591.5 (326.0 – 858.0)	635.0 (354.0 – 887.0)
VACS Index 2.0 score, median (IQR)	46.0 (39.0 – 57.0)	40.0 (33.0 – 51.0)	43.5 (32.0 – 56.5)	43.0 (31.0 – 56.0)
**Location**				
Brooklyn, NY	4 (25.0%)	6 (16.7%)	35 (26.9%)	37 (27.2%)
Manhattan, NY	8 (50.0%)	17 (47.2%)	55 (42.3%)	59 (43.4%)
New Haven, CT	4 (25.0%)	12 (33.3%)	36 (27.7%)	37 (27.2%)
Bridgeport, CT	0(0%)	1 (2.8%)	4 (3.1%)	3 (2.2%)

Abbreviations: eCO=exhaled carbon monoxide; IQR= interquartile range; HSI=heaviness of smoking index

- **a.** Not mutually exclusive categories
- **b.** Based on Heaviness of smoking index
- **c.** Based on scale from 1 to 10, where 1=not ready, 10=ready.
- **d.** Based on the WI-PREPARE score
- **e.** Based on the ASSIST-Lite
- **f.** Based on the Personal Health Questionnaire (PHQ)-8, score >9

© 2026 Edelman EJ et al. *JAMA Network Open*.

eTable 2. Effects of adaptive strategies on cigarettes per day and abstinence at week 12 and week 24 (strategies n=323)

	Adaptive strategy 1	Adaptive strategy 2	Adaptive strategy 3	Adaptive strategy 4
Stage 1 treatment	NRT	NRT	NRT+CM	NRT+CM
Stage 2 treatment	Oral MTUD (Switch)	NRT+CM (Intensify)	Oral MTUD+CM (Switch)	NRT+CM+ (Intensify)
Cigarettes				
12 week, LSMEANS (99% CI)	5.0 (3.6, 6.5)	5.0 (3.6, 6.5)	4.8 (3.4, 6.1)	4.8 (3.4, 6.1)
24 week, LSMEANS (99% CI)	6.4 (4.5,8.4)[2,3,4]	2.6 (1.1, 4.1)[1]	3.9 (2.4, 5.5)[1]	4.2 (2.6, 5.9)[1]
MI				
12 week, LSMEANS (99% CI)	5.1 (3.8, 6.5)	5.13 (3.8, 6.5)	4.8 (3.4, 6.1)	4.8 (3.4, 6.1)
24 week, LSMEANS (99% CI)	6.2 (4.5, 8.0)[2,3,4]	3.0 (1.5, 4.4) [1]	4.0 (2.5, 5.5)[1]	4.2 (2.6, 5.8)[1]
Abstinence				
12 week, % (99%CI)	12.3 (5.9, 23.9)	12.3 (5.9, 23.9)	29.6 (17.4, 45.6)	29.6 (17.4, 45.6)
24 week, % (99%CI)	11.4 (4.2, 27.8)[3,4]	12.8 (5.2, 28.3)[4]	25.1 (12.8, 43.4)[1]	30.0 (16.2, 48.7)[1,2]
MI				
12 week, % (99%CI)	15.1 (7.4, 28.3)	15.1 (7.4, 28.3)	32.6 (19.9, 48.5)	32.6 (19.9, 48.5)
24 week, % (99%CI)	13.3 (4.6, 32.8)	15.3 (6.4, 32.2)	26.0 (11.6, 48.4)	31.4 (15.6, 53.0)
Imp Not Abs*				
12 week, % (99%CI)	9.7 (4.7-19.2)	9.7 (4.7-19.0)	23.2 (13.3-37.3)	23.2 (13.3-37.3)
24 week, % (99%CI)	10.3 (3.8-24.5)	10.4 (4.2-23.4)	18.5 (9.4-33.3)	25.2 (13.5-42.2)

*Imp Not Abs= imputation not abstinent, single imputation at 12 and 24 weeks of not abstinent and superscript indicates significantly different at p<0.01. MI=multiple imputation. Model covariates include site, age, heaviness of smoking index, and sex.

© 2026 Edelman EJ et al. *JAMA Network Open*.

eTable 3. Number of participants experiencing adverse events in stage 1 and stage 2 treatment by group

A. Stage 1

Adverse Event	NRT (n=163)	NRT+CM (n=160)
Infections - pathogen unspecified	1 (0.6%)	1 (0.6%)
Gastrointestinal (nausea, diarrhea, abdominal pain)	3 (1.8%)	2 (1.3%)
Headaches	1 (0.6%)	0 (0%)
Sleep disorders and disturbances	1 (0.6%)	2 (1.3%)
Neurological disorders (sedation/lethargy dizziness)	3 (1.8%)	0 (0%)
Suicidality	0 (0%)	2 (1.3%)
Respiratory disorders	3 (1.8%)	3 (1.9%)
Liver injury or dysfunction (increase in AST/ALT 5x upper limit of normal)	1 (0.6%)	0 (0%)
Chest Pain	0 (0%)	1 (0.6%)
Reaction to nicotine replacement therapy	0 (0%)	3 (1.9%)
Other	6 (3.7%)	10 (6.3%)
Death	1 (0.6%)	2 (1.3%)

a. The nine adverse events that were considered possibly or probably protocol-related were pertinent to known side effects with nicotine replacement therapy and all were considered mild in nature (e.g., vivid dreams, sleeplessness, dry mouth from lozenges, skin irritation, heartburn with NRT, palpitations, feeling "shaky").

© 2026 Edelman EJ et al. *JAMA Network Open*.

B. Stage 2 treatment

Adverse Event	Switch to oral MTUD, n=130	NRT alone, n=16	NRT+CM, n=36	Intensify CM, n=136
Infections - pathogen Unspecified	1 (0.8%)	0 (0%)	0 (0%)	0 (0%)
Gastrointestinal (nausea, diarrhea, abdominal pain)	3 (2.3%)	1 (6.3%)	0 (0%)	0 (0%)
Joint pain and/or connective tissue disorders	0 (0%)	0 (0%)	1 (2.8%)	0 (0%)
Neurological disorders (sedation/lethargy dizziness)	0 (0%)	1 (6.3%)	0 (0%)	0 (0%)
Suicidality	1 (0.8%)	0 (0%)	0 (0%)	0 (0%)
Respiratory disorders	1 (0.8%)	0 (0%)	0 (0%)	2 (1.5%)
Liver injury or dysfunction (increase in AST/ALT 5x upper limit of normal)	1 (0.8%)	0 (0%)	0 (0%)	0 (0%)
Pneumonia	1 (0.8%)	0 (0%)	0 (0%)	1 (0.7%)
Chest Pain	1 (0.8%)	0 (0%)	0 (0%)	0 (0%)
Other	1 (0.8%)	0 (0%)	1 (2.8%)	5 (3.7%)
Death	1 (0.8%)	0 (0%)	0 (0%)	2 (1.5%)

a. *These two adverse events that were considered possibly or probably protocol-related were pertinent to known side effects with varenicline and considered mild (n=1) or moderate (n=1) in nature (e.g., diarrhea, nausea, abdominal pain).

© 2026 Edelman EJ et al. *JAMA Network Open*.

eFigure 1. Clinical pharmacist visit completion by stage and treatment group

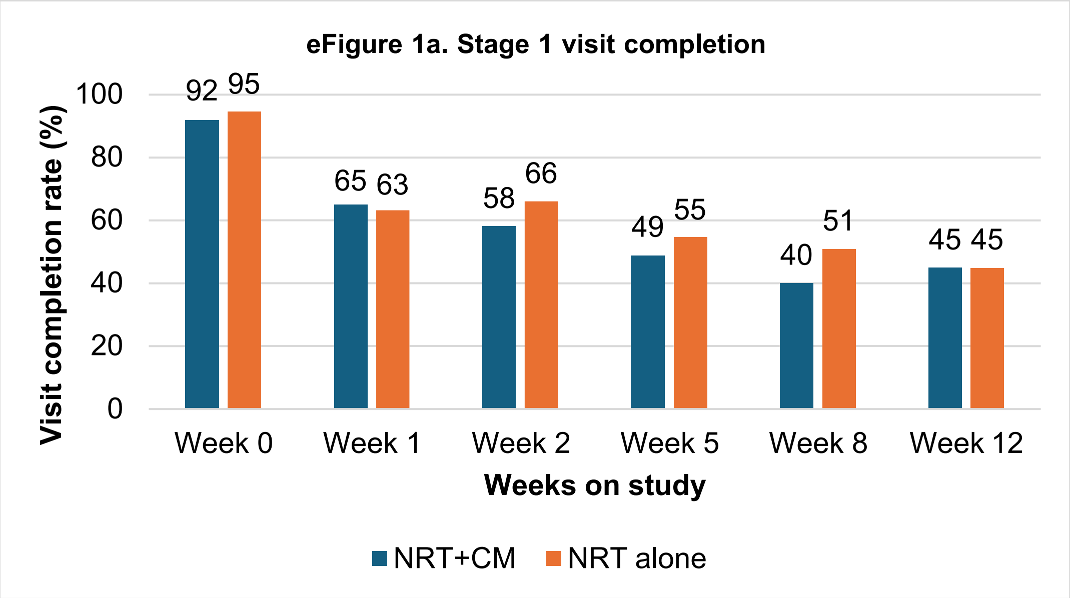

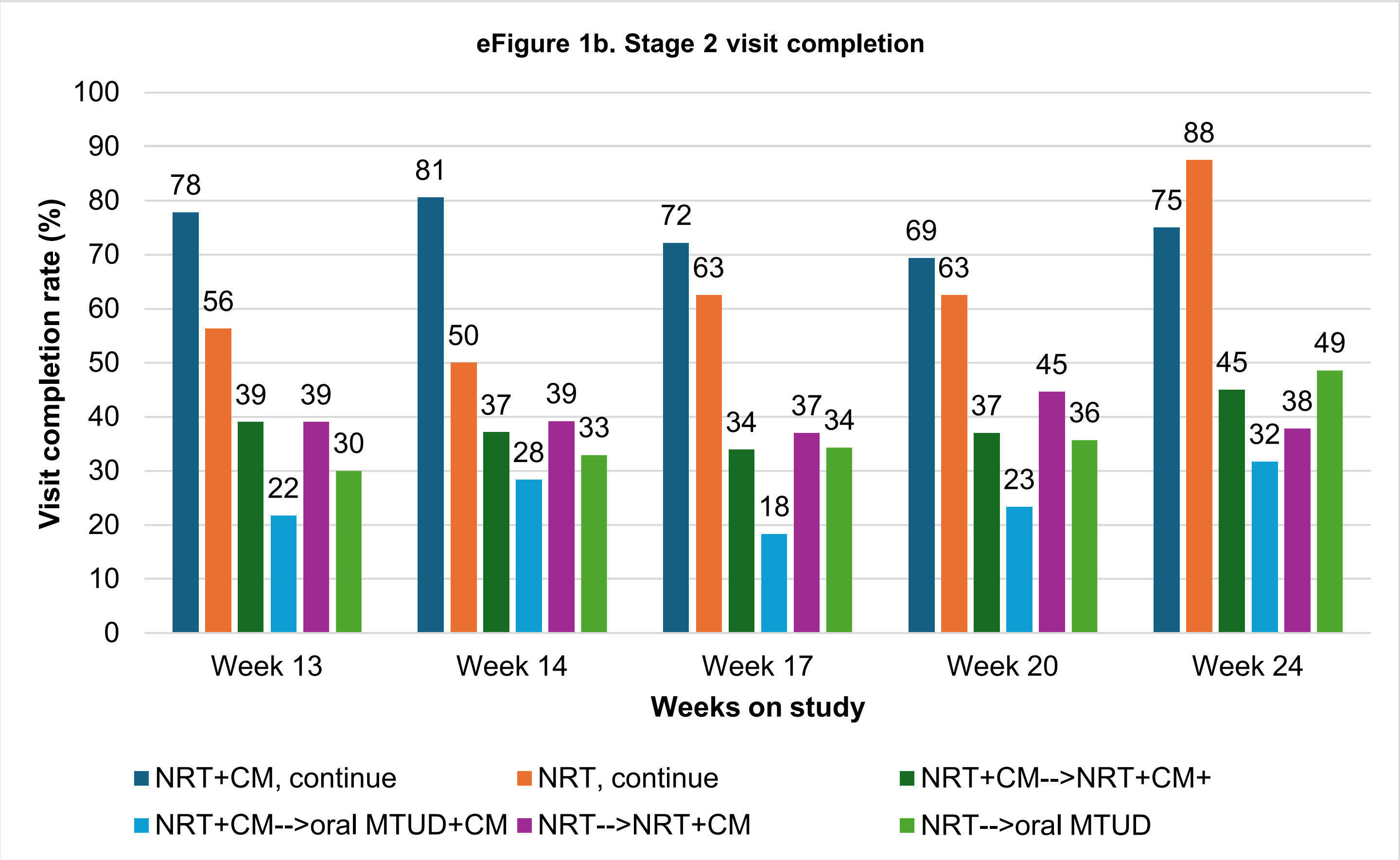

© 2026 Edelman EJ et al. *JAMA Network Open*.

eFigure 2. MTUD prescription at first visit by stage, treatment group, and medication

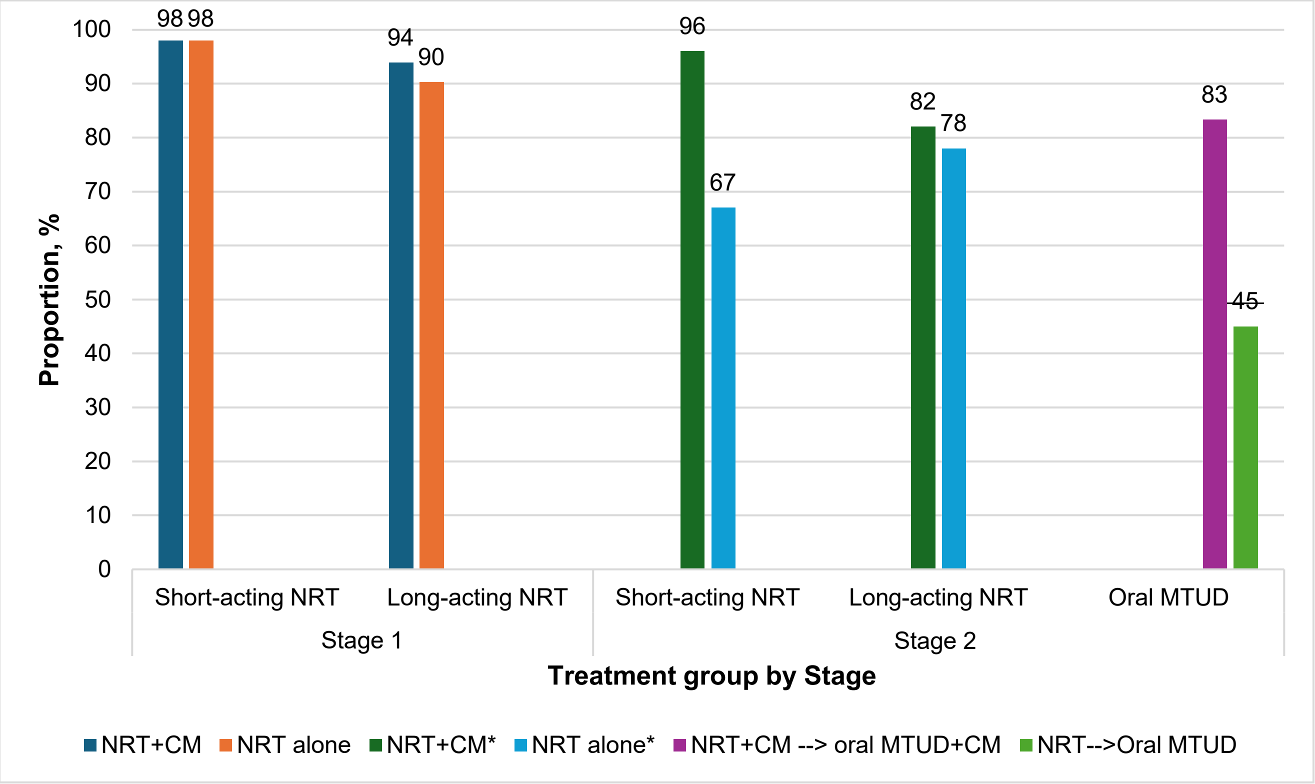

a. Includes participants with response to Stage 1 treatment

b. Among participants randomized to NRT+CM → oral MTUD+CM, 60% (6/10) were prescribed varenicline and 30% (3/10) were prescribed bupropion.

c. Among participants randomized to NRT alone → oral MTUD+CM, 77% (7/9) were prescribed varenicline and 11% (1/9) were prescribed bupropion.

(Note: data on type of medication prescribed were not specified for one participant randomized to NRT+CM at first visit of Stage 2 and for one participant randomized to NRT at first visit of Stage 2.)

© 2026 Edelman EJ et al. *JAMA Network Open*.
