# Supplementary material for: Clinical Pharmacists, Medications, and Contingency Management for Targeting Smoking in HIV Clinics: A Randomized Clinical Trial
Source: JAMA Netw Open. 2026 Feb 27;9(2):e2560593. doi: 10.1001/jamanetworkopen.2025.60593 (PMC12949440; doi:10.1001/jamanetworkopen.2025.60593)
Supplement: Supplement 3. — Data Sharing Statement [file jamanetwopen-e2560593-s003.pdf]

Data Sharing Statement

Edelman. Clinical Pharmacists, Medications, and Contingency Management for Targeting Smoking in HIV Clinics. *JAMA Netw Open*. Published February 27, 2026.
doi:10.1001/jamanetworkopen.2025.60593

Data
Additional Information: NCT04490057
Data available: Yes
Data types: Deidentified participant data, Data dictionary
How to access data: ejennifer.edelman@yale.edu
When available: With publication

Supporting Documents
Document types: Other (please specify)
Additional Information: We are happy to make documents available upon request.
How to access documents: ejennifer.edelman@yale.edu
When available: With publication

Additional Information
Who can access the data: Researchers whose proposed use of the data has been approved.
Types of analyses: Analyses for which we have relevant requested data.
Mechanisms of data availability: ejennifer.edelman@yale.edu
